# Supplementary material for: The Ebola virus VP40 matrix layer undergoes endosomal disassembly essential for membrane fusion
Source: EMBO J. 2023 Apr 21;42(11):e113578. doi: 10.15252/embj.2023113578 (PMC10233383; doi:10.15252/embj.2023113578)
Supplement: Supplementary file 1 — Appendix [file EMBJ-42-e113578-s002.pdf]

Appendix

The Ebola virus VP40 matrix layer undergoes endosomal disassembly essential for membrane fusion

Sophie L. Winter[1,2], Gonen Golani[2,3], Fabio Lolicato[4,5], Melina Vallbracht[1,2], Keerthihan Thiyagarajah[1,2], Samy Sid Ahmed[6], Christian Lüchtenborg[4], Oliver T. Fackler[6,7], Britta Brügger[4], Thomas Hoenen[8], Walter Nickel[4], Ulrich S. Schwarz[2,3], Petr Chlanda[1,2]*

[1] Schaller Research Groups, Department of Infectious Diseases, Virology, University Hospital Heidelberg, Heidelberg, Germany
[2] BioQuant-Centre for Quantitative Biology, Heidelberg University, Heidelberg, Germany
[3] Institute for Theoretical Physics, Heidelberg University, Heidelberg, Germany
[4] Heidelberg University Biochemistry Center, Heidelberg, Germany
[5] Department of Physics, University of Helsinki, Helsinki, Finland
[6] Department of Infectious Diseases, Integrative Virology, University Hospital Heidelberg, Heidelberg, Germany
[7] German Centre for Infection Research (DZIF), Partner Site Heidelberg, Germany
[8] Institute of Molecular Virology and Cell Biology, Friedrich-Loeffler-Insitut, Greifswald-Insel Riems, Germany

*Correspondence: chlanda@bioquant.uni-heidelberg.de

Table of Contents

Appendix Figure S1 2
Appendix Figure S2 3
Appendix Figure S3 4
Appendix Figure S4 5
Appendix Figure S5 6

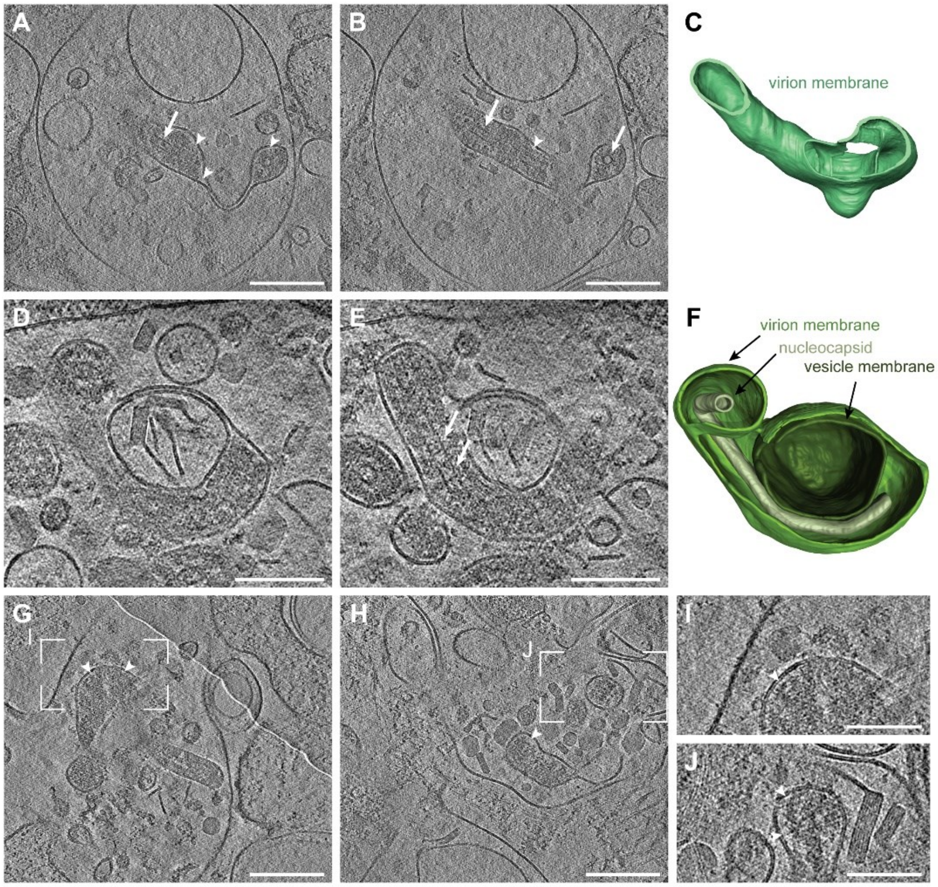

Appendix Figure S1

In situ cryo-ET of EBOV infecting Huh7 cells. Slices through tomograms showing Ebola virions inside late endosomal compartments. All virions display condensed nucleocapsids (white arrows) and disassembled VP40 layers, which have detached from the viral membrane as apparent from the gap adjacent to the inner lipid monolayer (white arrowheads). **(A-B)** Different slices through the same tomogram showing an internalized EBOV with a disassembled VP40 matrix and highly flexible membrane. The nucleocapsid is still condensed (white arrow). **(C)** 3D segmentation of the lipid envelope of the EBOV virion (green) shown in (A) and (B). **(D-E)** Different slices through the same tomogram showing an internalized EBOV virion with a disassembled VP40 matrix and condensed nucleocapsid. The virion had engulfed an intraluminal vesicle containing cholesterol ester crystals, indicating that this virus has undergone fusion. **(F)** 3D segmentation of EBOV shown in (D) and (E) showing the viral membrane (green), nucleocapsid (light green) and vesicle membrane (dark green). Scale bars: (A), (B): 200 nm, (D-J): 100 nm.

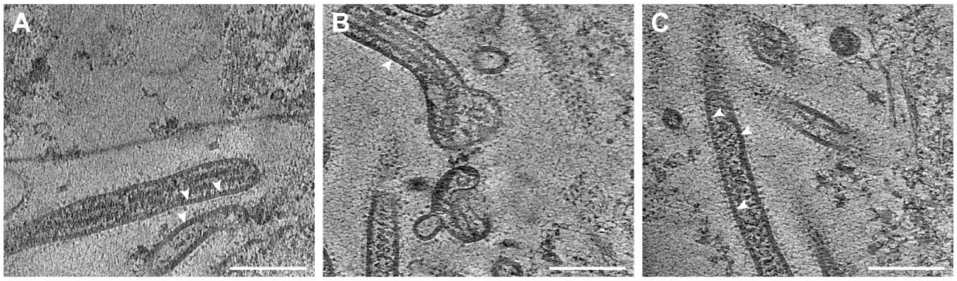

Appendix Figure S2

In situ cryo-ET of budding and released EBOV from infecting Huh7 cells. (A-C) Slices through tomograms showing Ebola virions adjacent to the plasma membrane of infected Huh7 cells. All virions contain assembled VP40 layers as apparent from the regular densities decorating the inner lipid monolayer at the luminal side (white arrowheads). Scale bars: 200 nm.

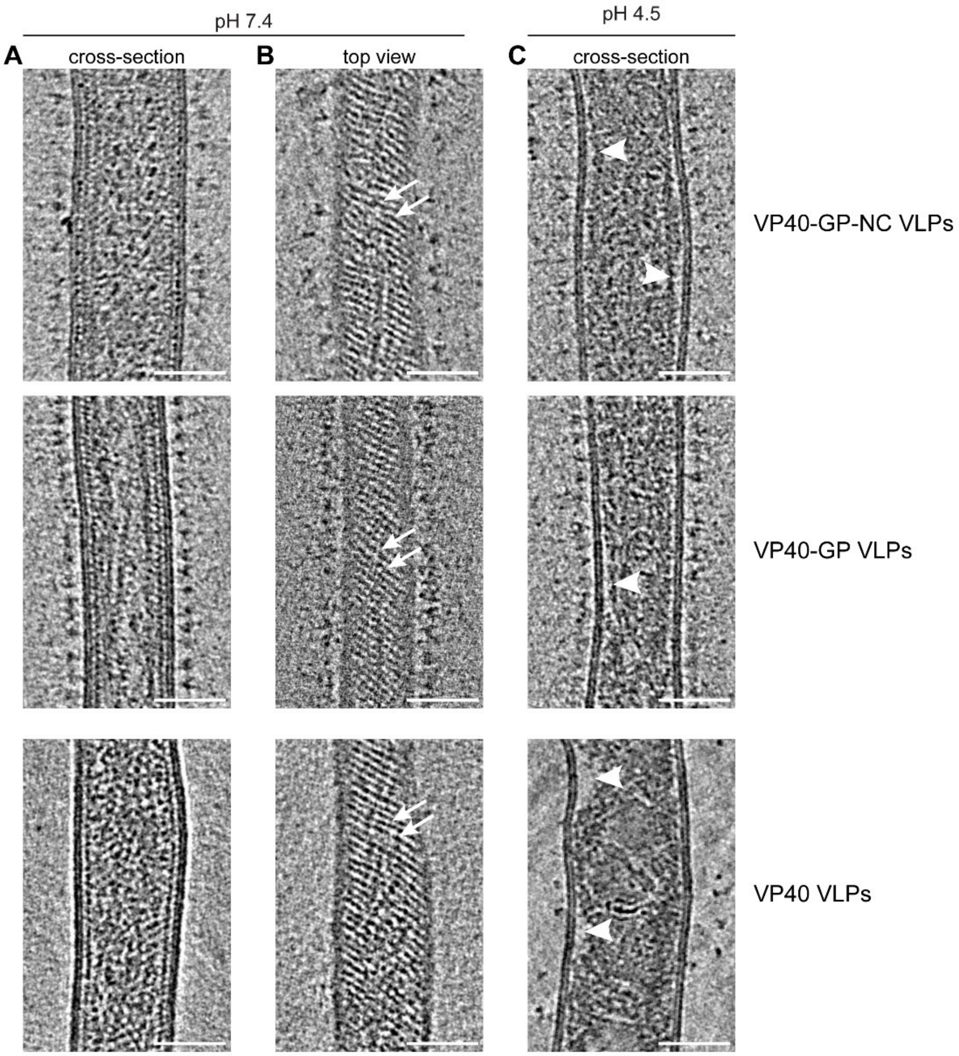

Appendix Figure S3

Structural characterization of unpurified EBOV VLPs of different protein composition. From top to bottom, the VLPs are composed of VP40, GP and the nucleocapsid (NC) proteins NP, VP24 and VP35; VP40 and GP; and VP40 alone. **(A)** Cross-sectional slices through tomograms showing filamentous VLPs at pH 7.4. **(B)** Near-to-surface slices of tomograms showing the top view of VLPs incubated at pH 7.4 and exhibiting the characteristic striations of the VP40 matrix (white arrows). **(C)** Cross-sectional slices through tomograms showing VLPs incubated at pH 4.5. No ordered VP40 matrix is visible adjacent to the VLP membrane as indicated by white arrowheads. Scalebars: 50 nm.

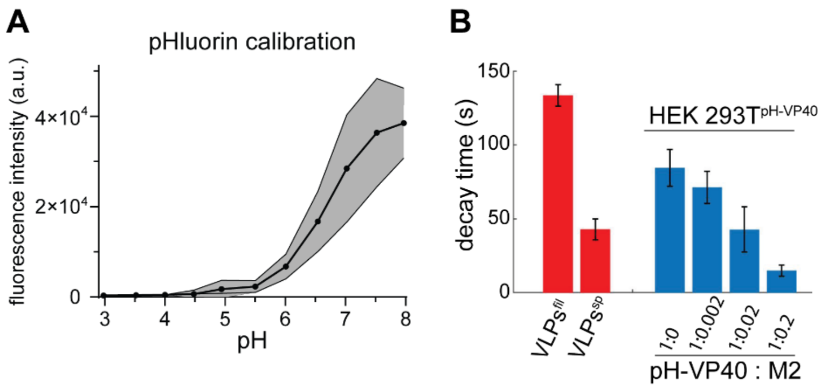

Appendix Figure S4

Calibration of pHluorin fluorescence and decay times. (A) Fluorescence intensity of HEK 293T cells expressing pHluorin-VP40 measured at 488 nm as a function of pH. Cells were grown in cell culture media before exchanging the media with HNE buffer (10 mM HEPES, 100 mM NaCl, 1 mM EDTA) at different pH. **(B)** pH characteristic decay times as found by fitting the pH levels to Eq. 3 of VLPs (red) and HEK 293T cells (blue) expressing pHluorin-VP40 and influenza M2 in increasing M2 levels. Filamentous VLPs 134±7 sec (n=154), spherical VLPs 43±7 sec (n=66), cells expressing VP40 only 84±12 sec (n=44), cells expressing VP40 and M2 at 1:0.002 molar ratio 71±11 sec (n=30), cells expressing VP40 and M2 at 1:0.02 molar ratio 43±15 sec (n=28), cells expressing VP40 and M2 at 1:0.2 molar ratio 15±4 sec (n=26).

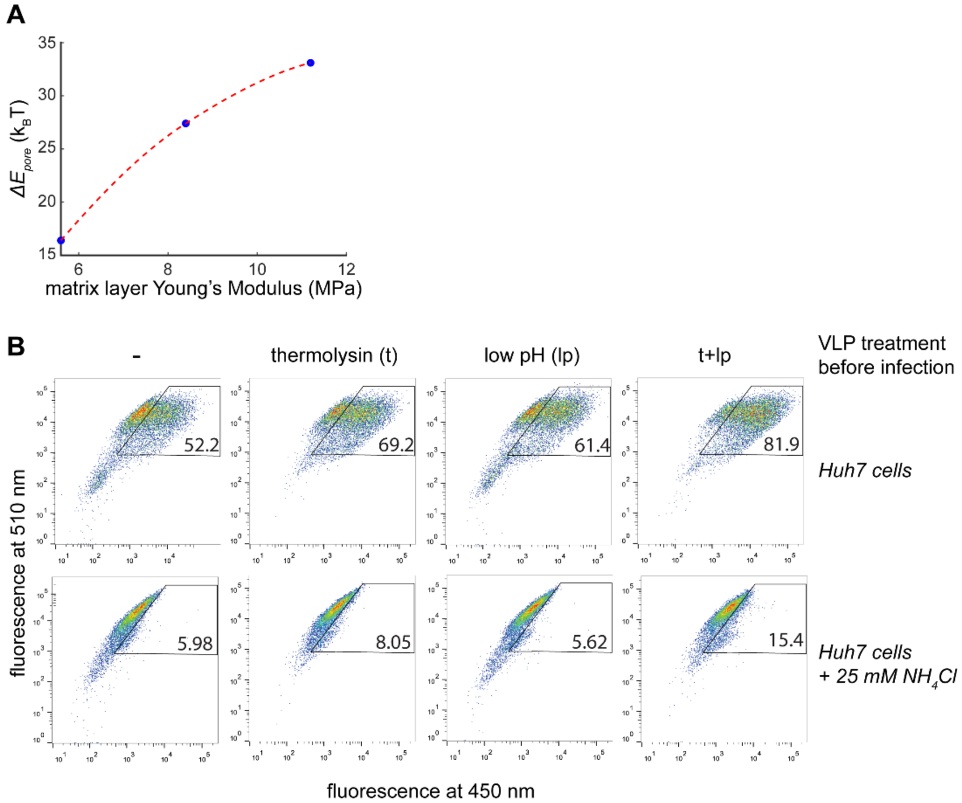

Appendix Figure S5

Entry of Ebola BlaM-VLPs into Huh7 cells. (A) Plot showing the dependence of the change in fusion pore formation energy between u_0=38 k_BT/nm^2 to u_0=0 (ΔE_pore) on the matrix layer Young's modulus. Dotted lines serve as a guide to the eye. **(B)** FACS plots showing virus entry as measured by a fluorescence shift of infected cells from emission at 510 nm (no entry) to 450 nm (entry). The top row indicates in vitro VLP treatments prior to infection including buffer control (-), thermolysin treatment, low pH treatment, and a combination of thermolysin and low pH. The first row of FACS data shows entry into Huh7 target cells, the second row shows entry into Huh7 cells treated with 25 mM ammonium chloride to neutralize acidic compartments and assess virus entry in the absence of acidification. FACS data are shown from one out of three repetitions, with 10000 cells measured per sample.
