# Supplementary material for: The Ebola virus VP40 matrix layer undergoes endosomal disassembly essential for membrane fusion
Source: EMBO J. 2023 Apr 21;42(11):e113578. doi: 10.15252/embj.2023113578 (PMC10233383; doi:10.15252/embj.2023113578)
Supplement: Supplementary file 2 — Expanded View Figures PDF [file EMBJ-42-e113578-s001.pdf]

Expanded View Figures

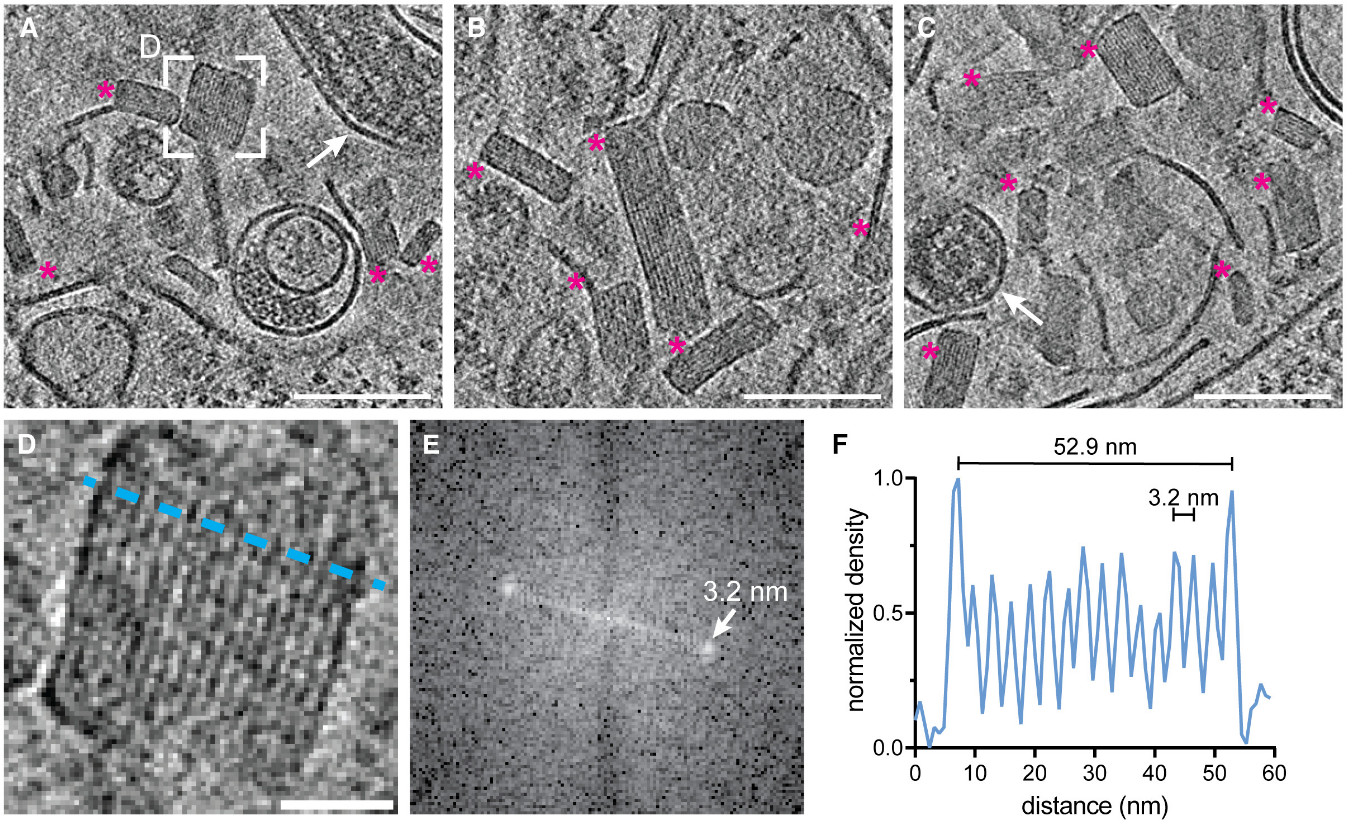

Figure EV1. Crystalline lipidic structures in endosomal compartments of EBOV-infected Huh7 cells.

A–C Slices through tomograms showing lumina of endosomal compartments crowded with crystalline lipidic structures (magenta asterisks). Two virions are highlighted with white arrows in (A) and (C).

D Magnified view of the area highlighted in (A) showing a cross-section through a crystalline lipidic structure. To determine the spacing between the stacked lipid monolayers, a line profile was determined (blue line).

E Fourier-transform analysis of the tomogram slice shown in (D) revealing a spacing of 3.2 nm.

F Line profile across the crystal shown in (D) showing the diameter of the structure along the short axis of 52.9 nm, and the regular 3.2 nm spacing of the lipid monolayers.

Data information: Scale bars: (A–C): 100 nm, (D): 20 nm.
Source data are available online for this figure.

© 2023 The Authors

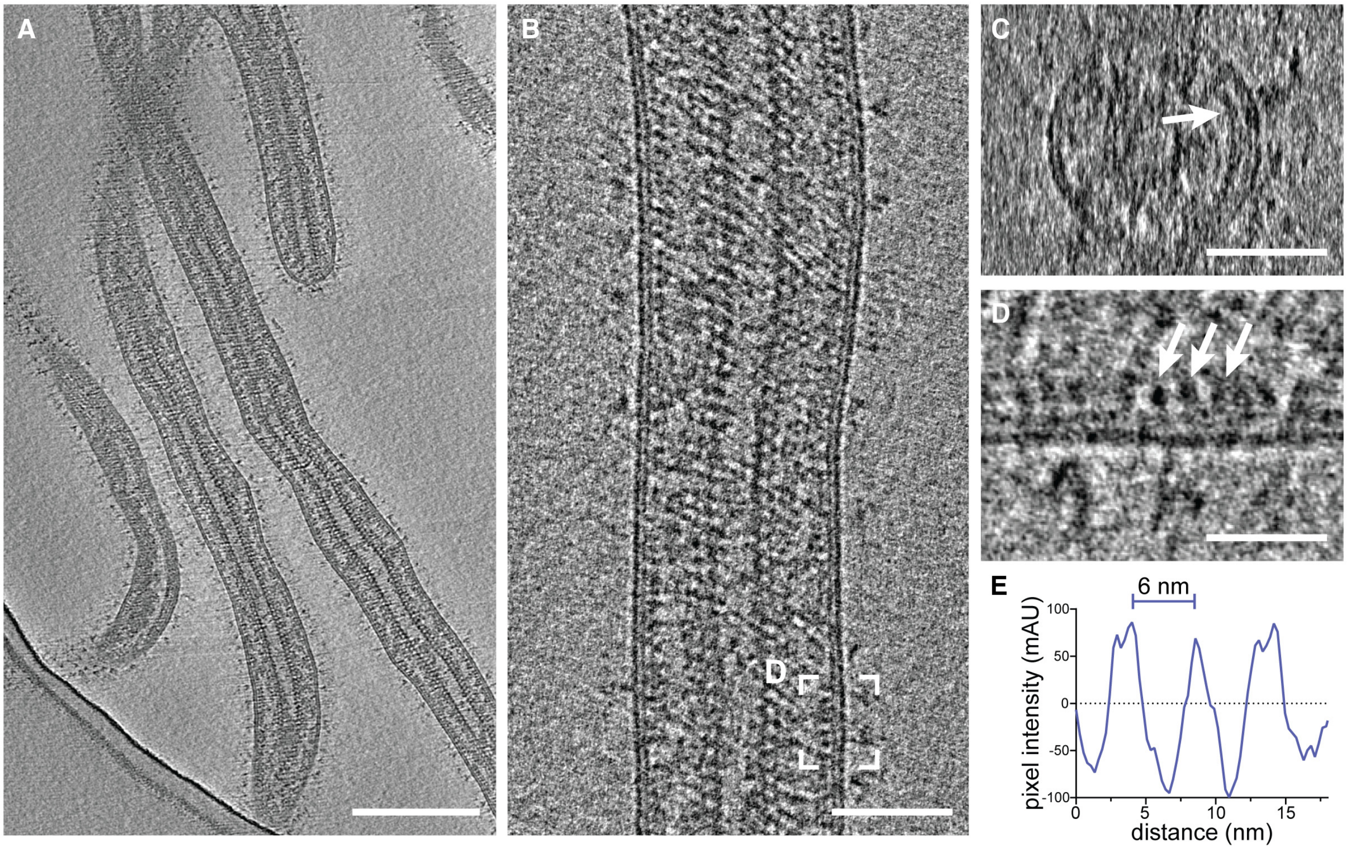

Figure EV2. Cryo-electron tomography of purified and chemically fixed EBOV.

A Slices through a tomogram showing an overview of filamentous virions. Condensed and decorated nucleocapsids span the length of each virion.
B, C Longitudinal and transverse cross-section, respectively, of a tomogram containing a filamentous EBOV.
C Transverse cross-section of the virion shown in (B). The VP40 matrix adjacent to the inner membrane monolayer is highlighted by a white arrow.
D Magnified area highlighted in (B) showing a longitudinal cross-section of the VP40 densities lining the inner membrane monolayer.
E Line profile determined adjacent to the inner monolayer of the virion shown in (B) showing the approximately 6 nm pitch of the VP40 matrix.

Data information: Scale bars: (A), (B): 200 nm, (C): 50 nm, (D): 20 nm.
Source data are available online for this figure.

 © 2023 The Authors

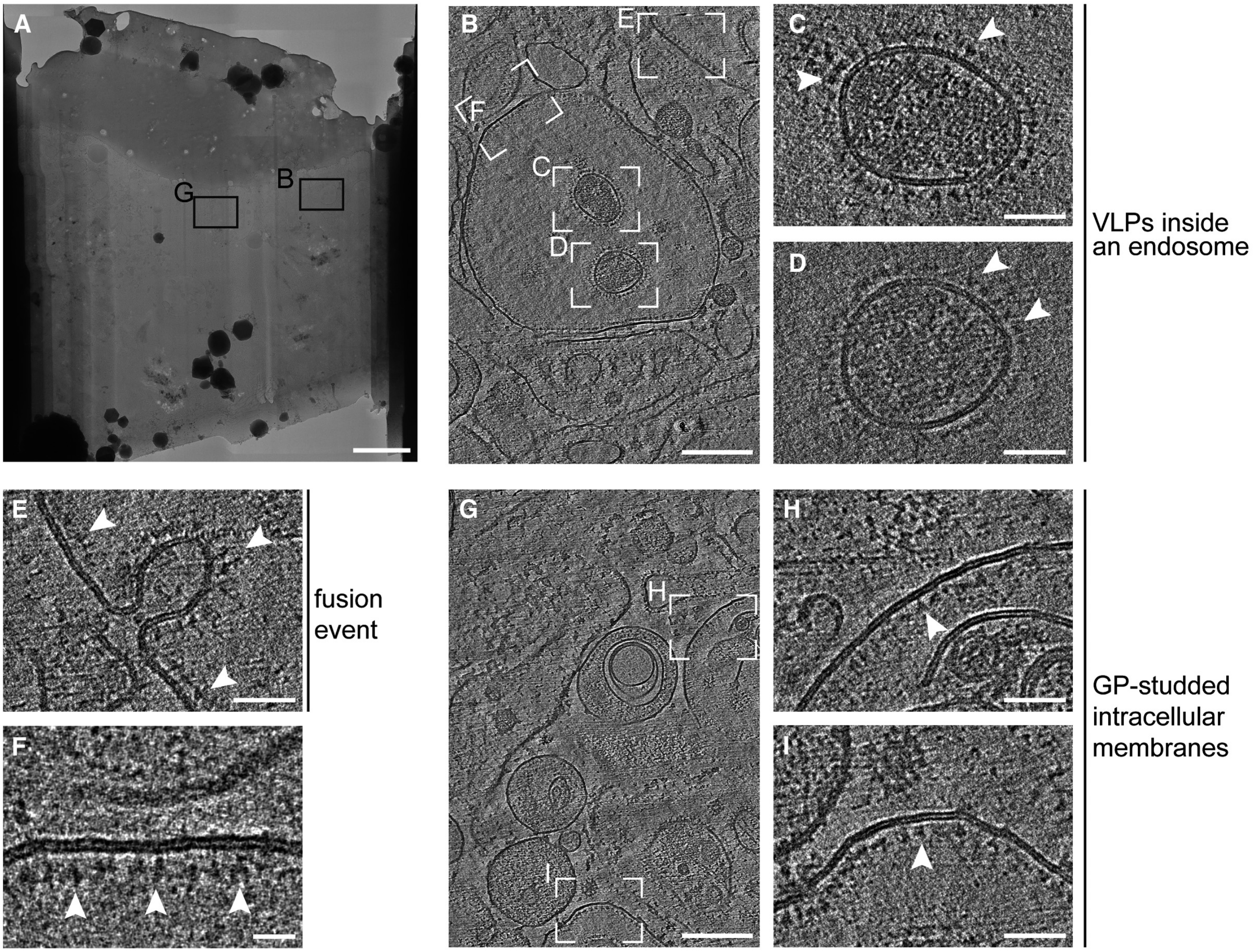

Figure EV3. ***In situ* cryo-ET of EBOV VLPs entering Huh7 cells.**

A Overview map of a lamella produced by cryo-FIB milling of an Huh7 cell incubated for 1 h with EBOV VLPs composed of VP40 and GP.
B Slices through a tomogram taken as indicated in (A).
C, D Areas highlighted in (B) showing cross-sections of VLPs inside a cellular compartment. The membranes surrounding the particles are studded with GPs (white arrowheads), while the lumina are crowded by disordered protein densities.
E Area highlighted in (B) showing an invagination studded with GPs (white arrowheads), likely representing a fusion event.
F Area highlighted in (B) showing a post-fusion event as indicated by the GPs facing the luminal side of the intracellular compartment.
G Slices through a tomogram taken as indicated in (A) showing post-fusion events characterized by intracellular membranes studded by GPs facing the luminal sites.
H, I Areas highlighted in (G) showing GP-decorated membranes (indicated by white arrowheads).

Data information: Scale bars: (A): 5 µm, (B), (G): 200 nm, (C–E), (H), (I): 50 nm, (F): 20 nm.
Source data are available online for this figure.

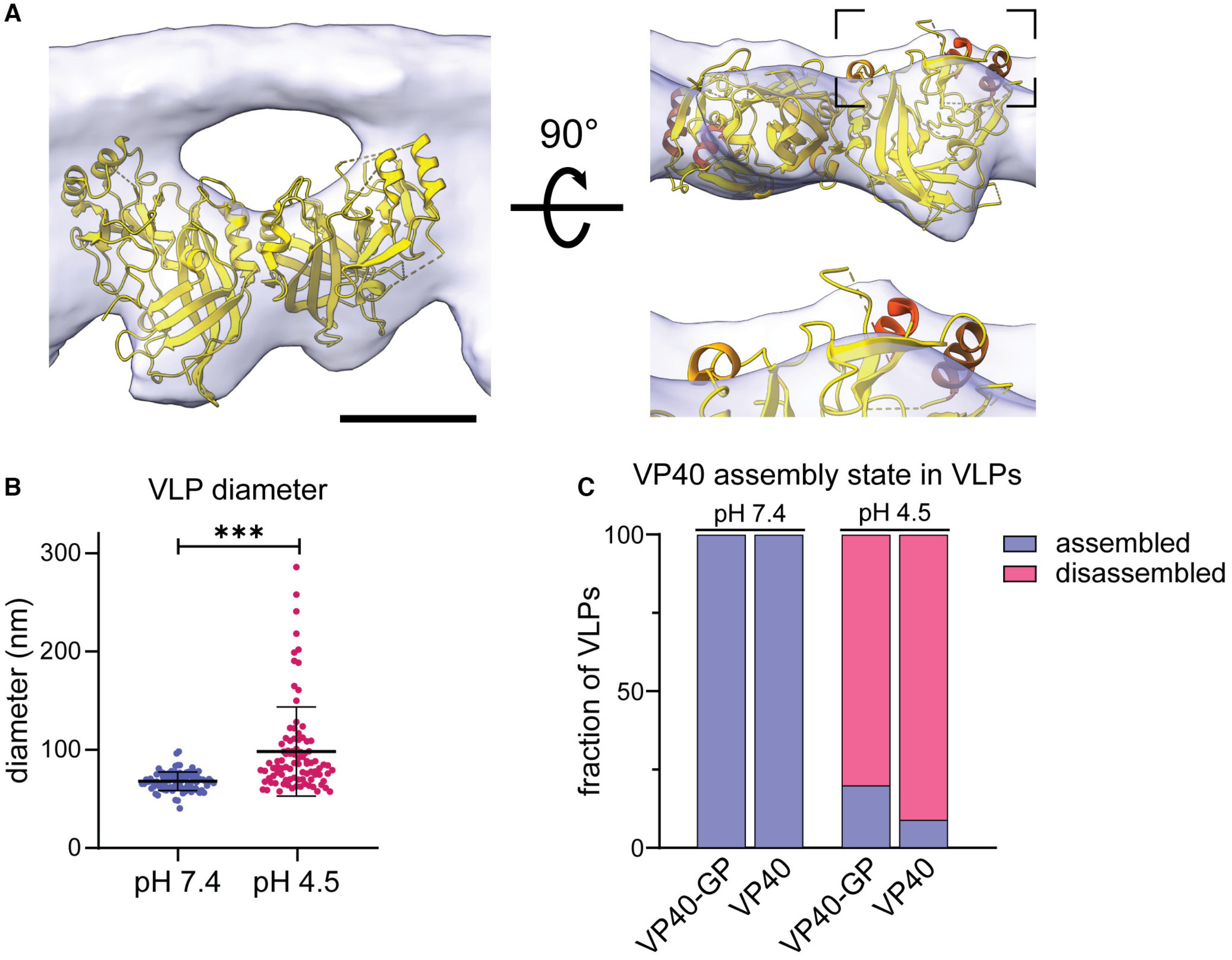

Figure EV4. Structural characterization of the VP40 matrix in VLPs by cryo-ET.

A VP40 dimer structure (pdb: 7jzj) fitted into the subtomogram average presented in Fig 2 from the side view including the density of the inner VLP monolayer and rotated by 90°. Helical segments protruding from the subtomogram average are highlighted in shades of orange.

B Diameter of VLPs composed of GP and VP40 measured from membrane-to-membrane after incubation at pH 7.4 and pH 4.5. Asterisks indicate statistical significance as judged by a two-tailed Welch's *t*-test, assuming unequal variance ($P < 0.0001$).

C Quantification of the VP40 assembly state of VLPs composed of VP40 and GP ($n = 37$ at pH 7.4 and 18 at pH 4.5); or VP40 alone ($n = 22$ at pH 7.4 and 8 at pH 4.5). The VP40 matrix was either assembled (blue), or disassembled (dark pink). VP40 assembly in VLPs subjected to neutral or low pH was assessed by cryo-ET.

Data information: Scale bars: (A) 2.5 nm.

 © 2023 The Authors

Figure EV5. Characterization of VP40 dimer angles and lipidomics.

A Superimposition of the crystallographic structure (pdb: 7jzj, yellow) with the membrane bound VP40 structure from the MD simulations (blue).
B The area highlighted in (A) shows the missing CDTs residues computationally modeled (green).
C Representation of the rotation angle of VP40 monomers along the NTD-dimerization domain.
D The dihedral angle between VP40 monomers is defined as the angle between the plane containing the vector connecting alpha carbon atoms of $L75^{monomer1}$ and $T112^{monomer1}$ and the vector connecting atoms $T112^{monomer1}$ and $T112^{monomer2}$ and the plane containing this second vector and the vector connecting atoms T112monomer2 and L75monomer2.
E Dihedral angle distribution shows that, regardless of pH, VP40 monomers within the dimer are flexible with a rotation angle oscillating around 1° (SD 9.5) in water, which is 17° smaller than the one measured for the crystallographic structure (pdb: 7jzj). VP40 dimer flexibility is not constrained upon binding to the membrane. However, after binding to the bilayer, the angle distribution was significantly ($P \leq 0.0001$) shifted to a value of 3.7° and 4.5° at pH 7.4 and 4.5, respectively. Unpaired *t*-tests were performed to evaluate the significance of differences in angle distributions.
F VP40-membrane internal energy calculated as the sum of short-range Coulomb and Lennard-Jonson interactions at neutral (orange) and low (red) pH. The internal energies have been calculated over the last 100 ns of the biased MD simulation windows centered at the membrane distance where the free energy minima were reconstructed (3.0 nm and 2.7 nm for neutral and low pHs, respectively). The error was estimated using the block averages over 5 blocks.
G Abundance of low-abundant lipids in the envelope of Ebola VLPs composed of GP, VP40, NP, VP24 and VP35. The mean abundance in mol% and values for each experiment ($n = 3$ biological replicates) are plotted together with the standard error of the mean (red). phosphatidylinositol (PI), phosphatidylglycerol (PG), phosphatidic acid (PA), ceramide (Cer), hexosylceramide (HexCer), cholesterol ester (CE) diacylglycerol (DAG), triacylglycerol (TAG). Prefix “a” indicates acyl-linked glycerophospholipids, prefix “e” indicates ether-linked (plasmanyl) or the presence of one odd and one even chain fatty acyl.
H Sequence alignment of VP40 proteins (amino acids 223–280) from all EBOV species (EBO) and Marburg virus (MAV). For each sequence, the uniprot identifiers are given on the left and the Ebolavirus species are indicated as ZM: Zaira Mayinga EBOV, SU: Sudan EBOV, RR: Reston EBOV, BB: Bundibugyo EBOV, TF: Tai Forest EBOV, B: Bombali virus. Conserved hydrophobic and hydrophilic amino acids are highlighted according to the color scheme of (Kyte & Doolittle, 1982). The hydrophilic amino acids K224, K225, K274 and K275 (indicated by arrows) are highly conserved among the EBOV species.

© 2023 The Authors

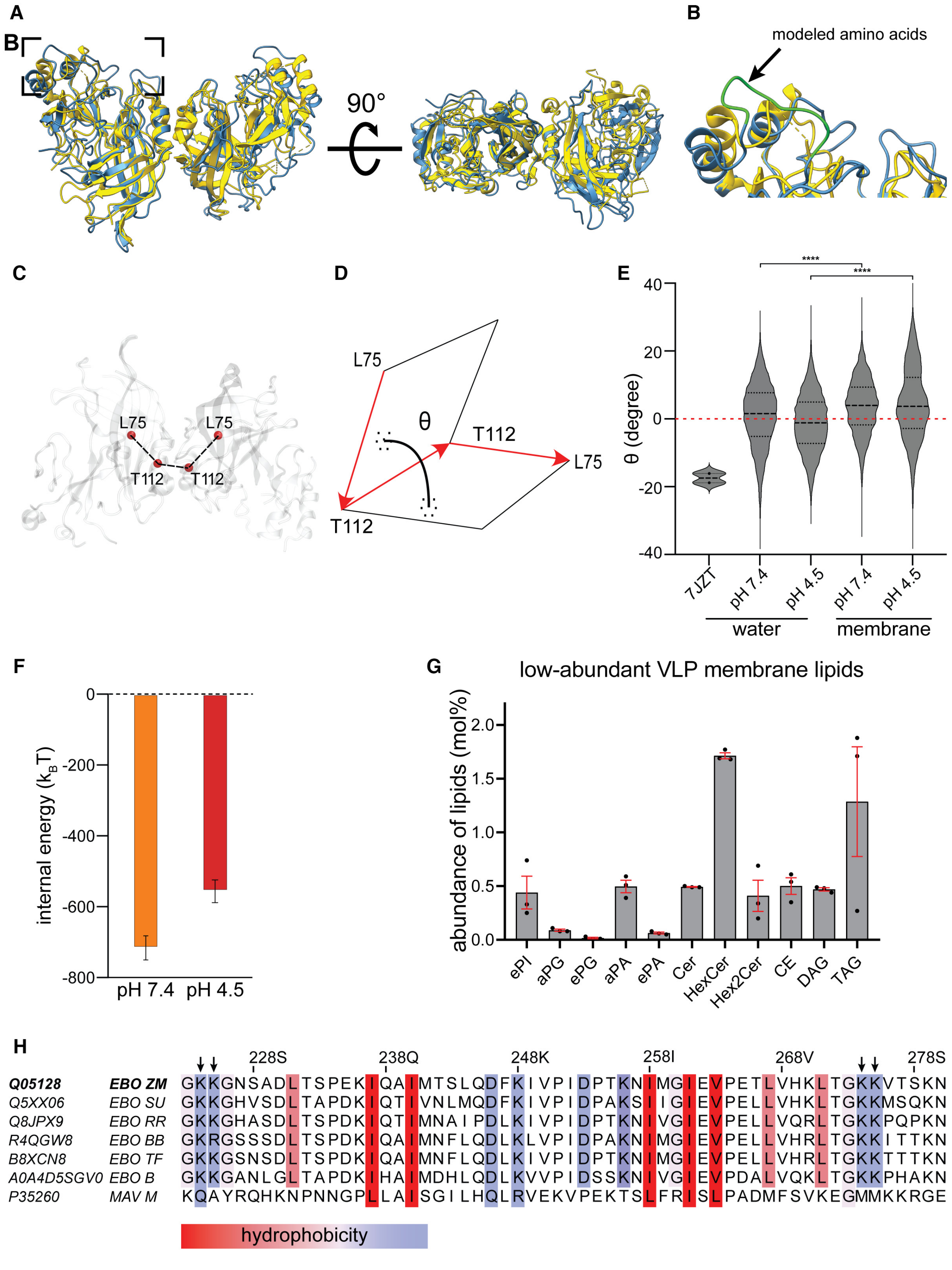

Figure EV5.

© 2023 The Authors
