# Supplementary material for: The Ebola virus VP40 matrix layer undergoes endosomal disassembly essential for membrane fusion
Source: EMBO J. 2023 Apr 21;42(11):e113578. doi: 10.15252/embj.2023113578 (PMC10233383; doi:10.15252/embj.2023113578)
Supplement: Supplementary file 5 — Movie EV1 [file EMBJ-42-e113578-s003.zip › Movie 1/Movie EV 1-legend.docx]

**Movie EV 1: *In situ* cryo-electron tomography of EBOV virions localized in endosomes of an infected cell**. Infected cells were grown on electron microscopy grids and chemical fixation using 4% PFA and 0.1% GA for biosafety reasons before removal from BSL4. Vitrification was performed prior to cell thinning by cryo-FIB milling and imaging by cryo-ET. Movie of a tomogram showing EBOV virions inside a late endosomal compartment. 3D segmentation of the delimiting endosomal membrane (yellow), cholesterol ester crystals (pink), viral membranes (shades of green) of three EBOV virions, and nucleocapsids (shades of light green) for visualization.
