# Supplementary figures and images for: The Ebola virus VP40 matrix layer undergoes endosomal disassembly essential for membrane fusion
Source: EMBO J. 2023 Apr 21;42(11):e113578. doi: 10.15252/embj.2023113578 (PMC10233383; doi:10.15252/embj.2023113578)

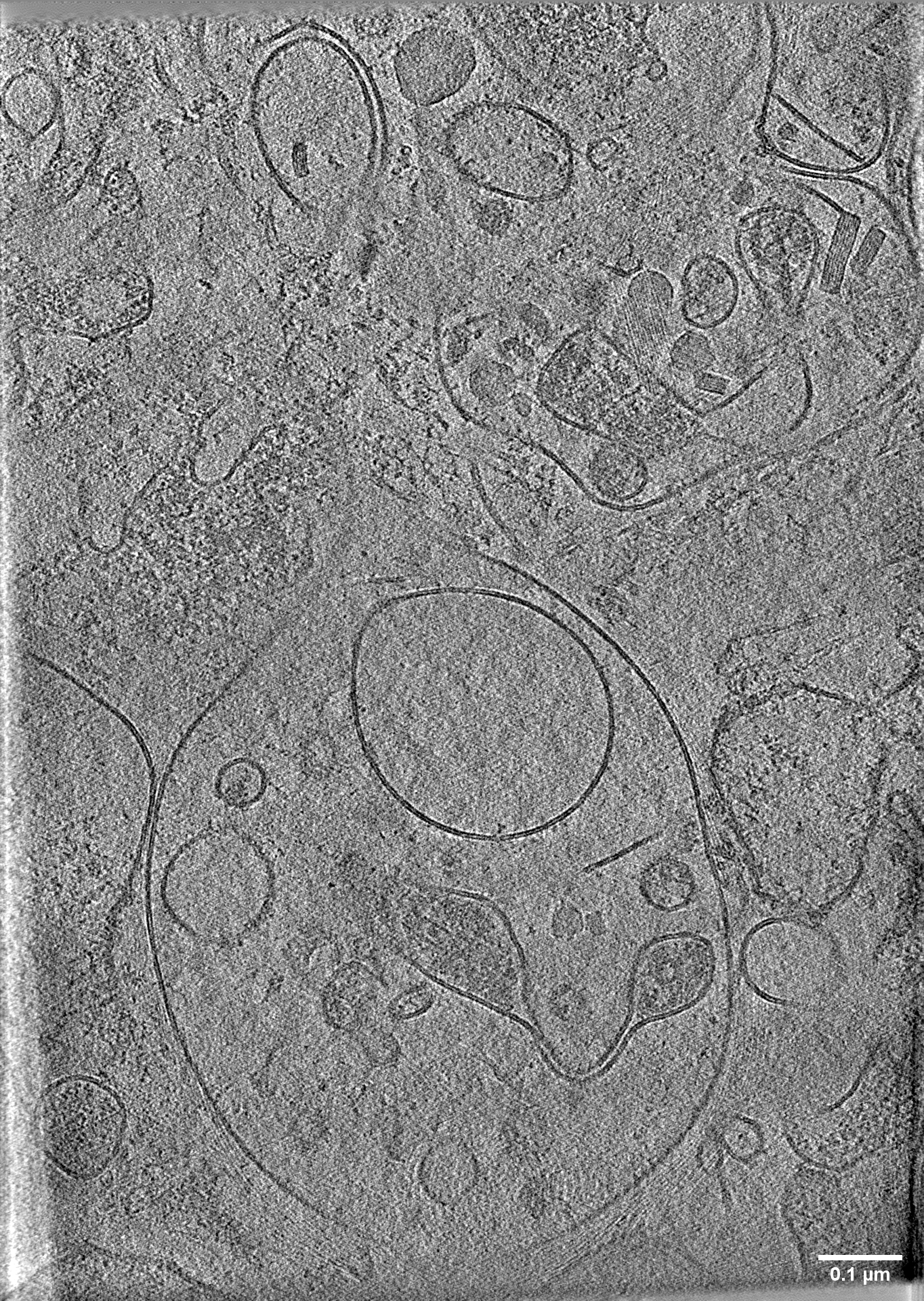

Supplement: Supplementary file 6 — Source Data for Expanded View and Appendix [file EMBJ-42-e113578-s006.zip › SupplementaryFigures_SourceData/AppendixFigureS1/PanelA/AVG10_TS_07_bin3_bandpass1_2.png]

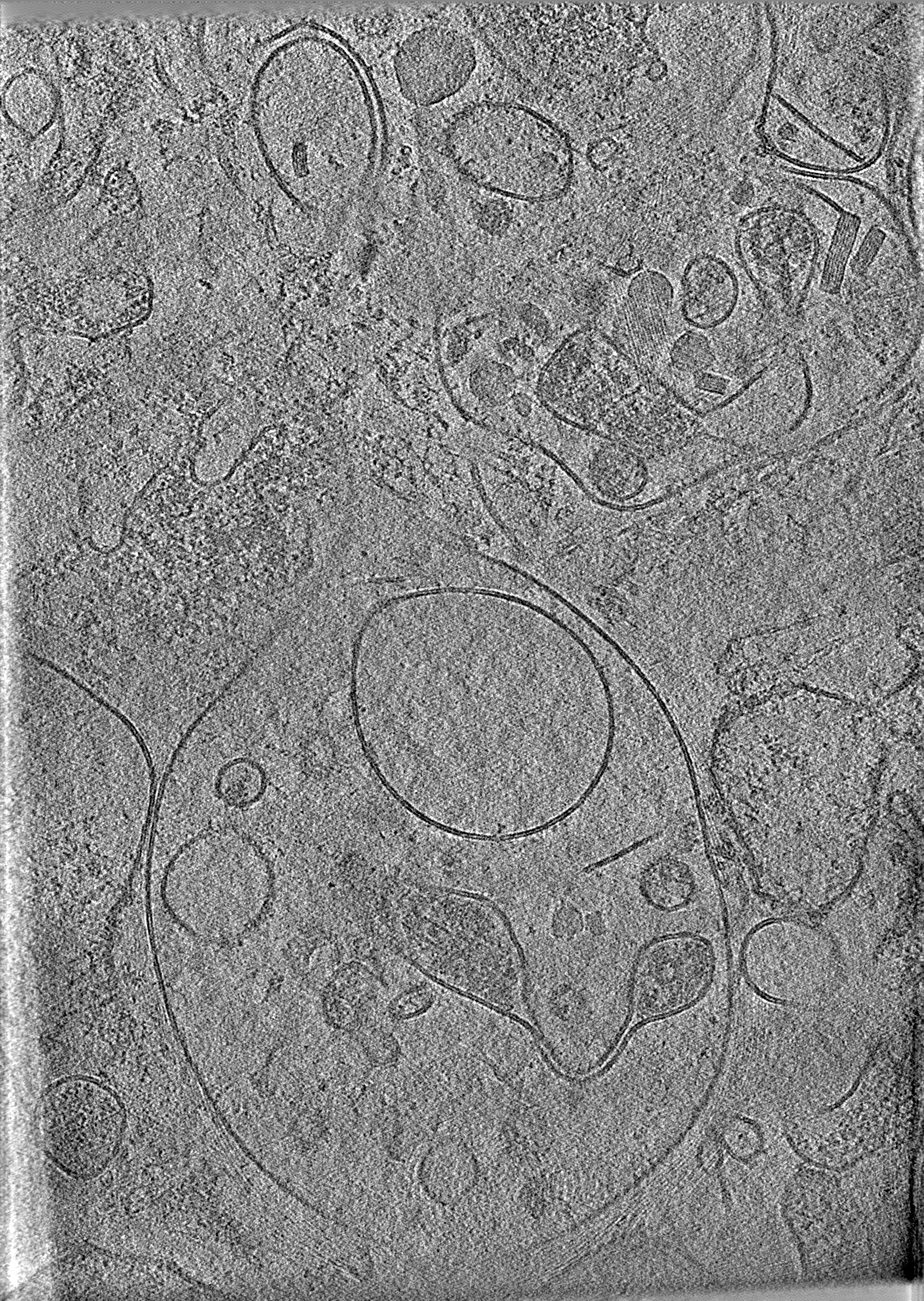

Supplement: Supplementary file 6 — Source Data for Expanded View and Appendix [file EMBJ-42-e113578-s006.zip › SupplementaryFigures_SourceData/AppendixFigureS1/PanelA/AVG10_TS_07_bin3_bandpass1_2.tif]

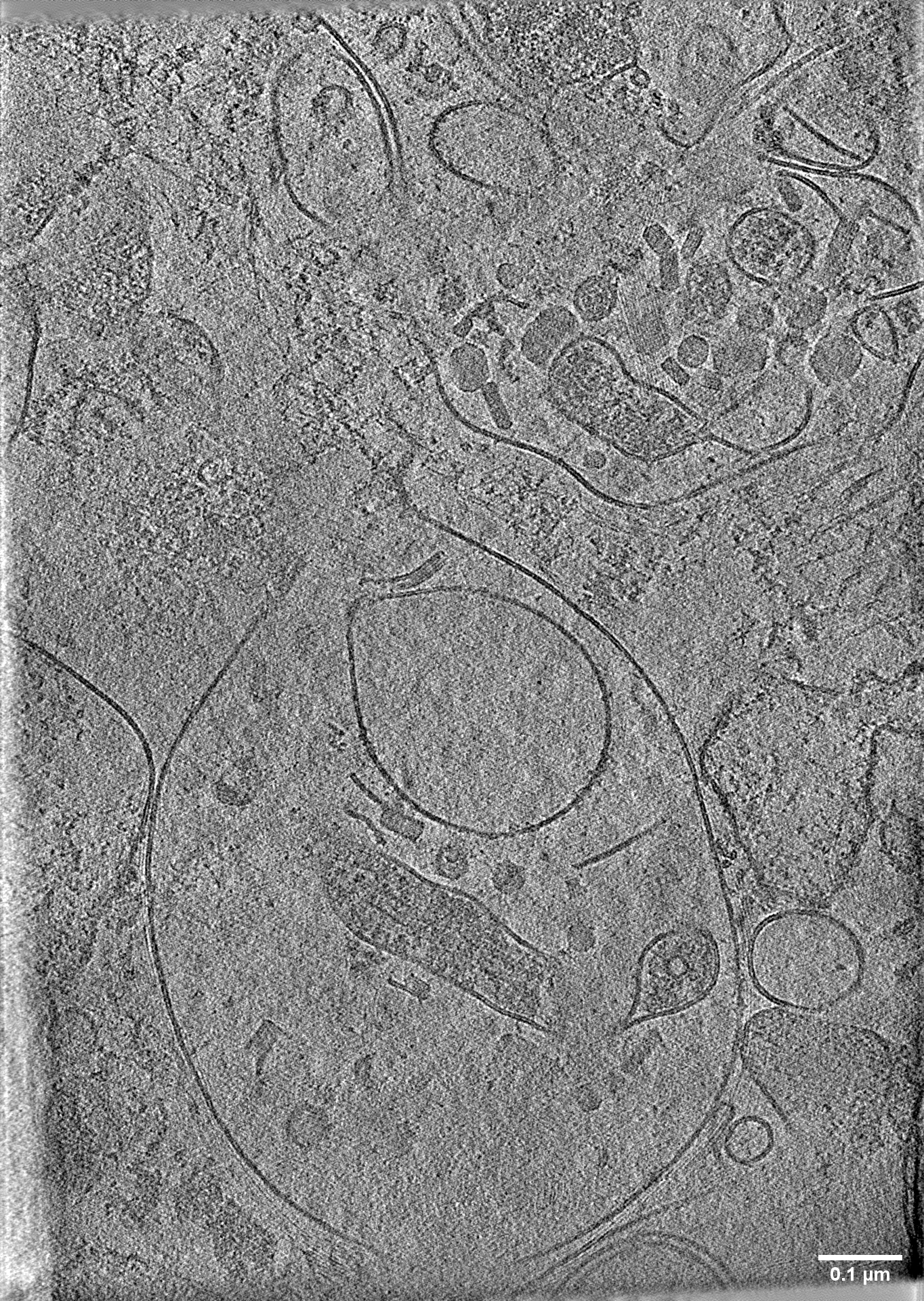

Supplement: Supplementary file 6 — Source Data for Expanded View and Appendix [file EMBJ-42-e113578-s006.zip › SupplementaryFigures_SourceData/AppendixFigureS1/PanelB/AVG10_TS_07_bin3_bandpass1.png]

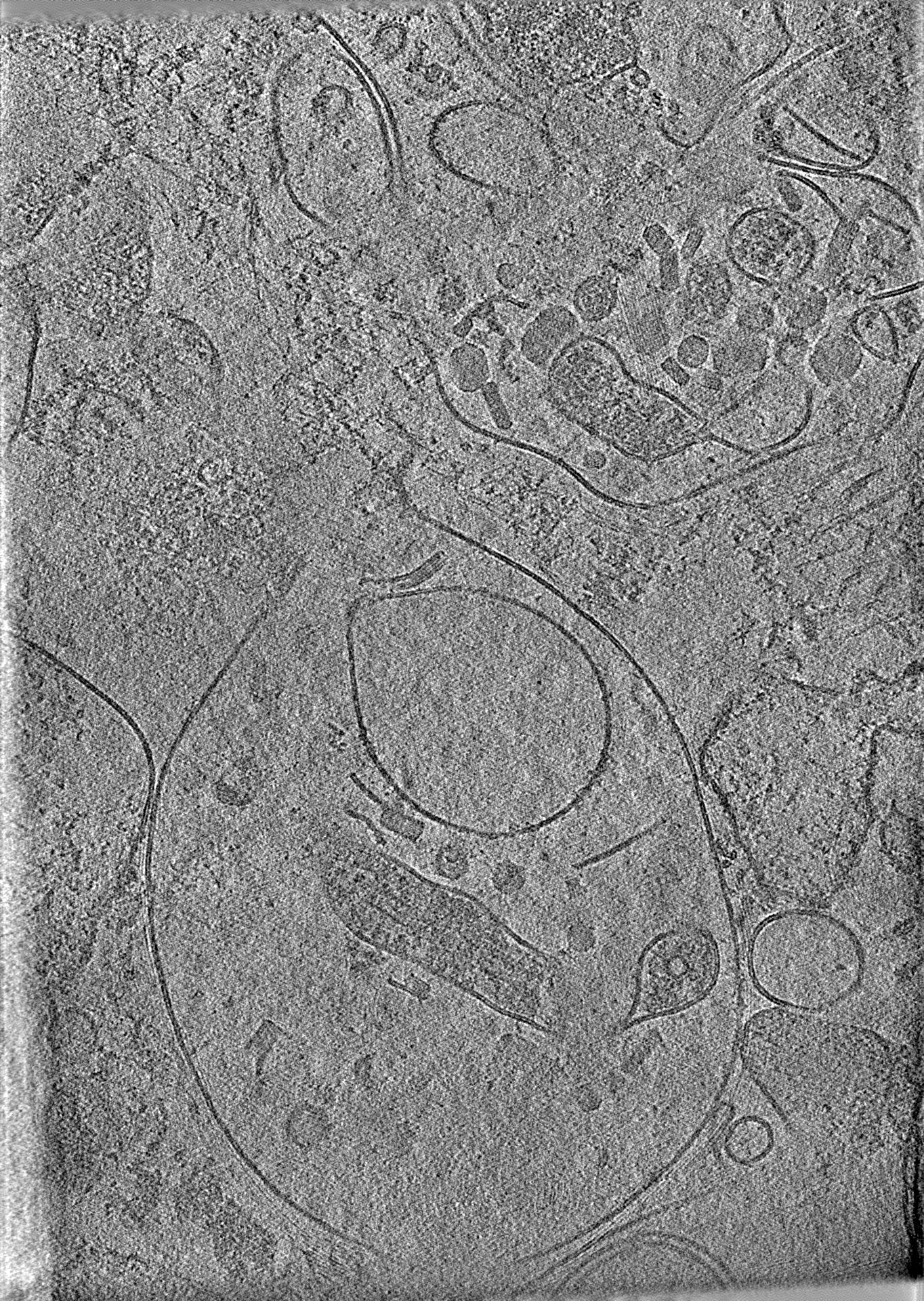

Supplement: Supplementary file 6 — Source Data for Expanded View and Appendix [file EMBJ-42-e113578-s006.zip › SupplementaryFigures_SourceData/AppendixFigureS1/PanelB/AVG10_TS_07_bin3_bandpass1.tif]

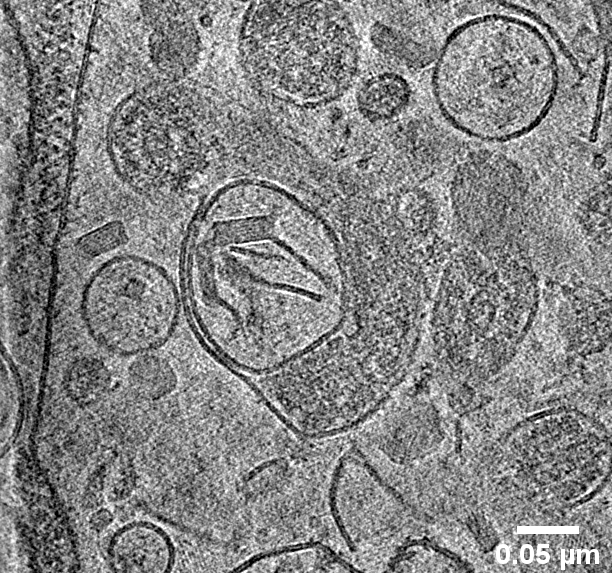

Supplement: Supplementary file 6 — Source Data for Expanded View and Appendix [file EMBJ-42-e113578-s006.zip › SupplementaryFigures_SourceData/AppendixFigureS1/PanelD/AVG10_TS_02_bin3_bandpass1.png]

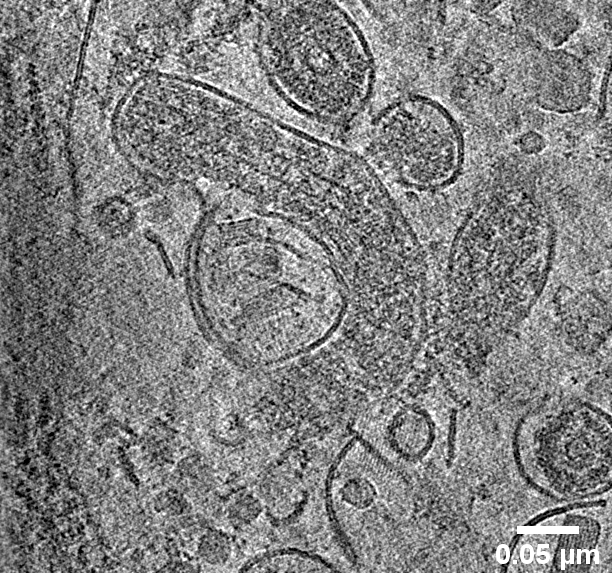

Supplement: Supplementary file 6 — Source Data for Expanded View and Appendix [file EMBJ-42-e113578-s006.zip › SupplementaryFigures_SourceData/AppendixFigureS1/PanelE/AVG10_TS_02_bin3_bandpass1_2.png]

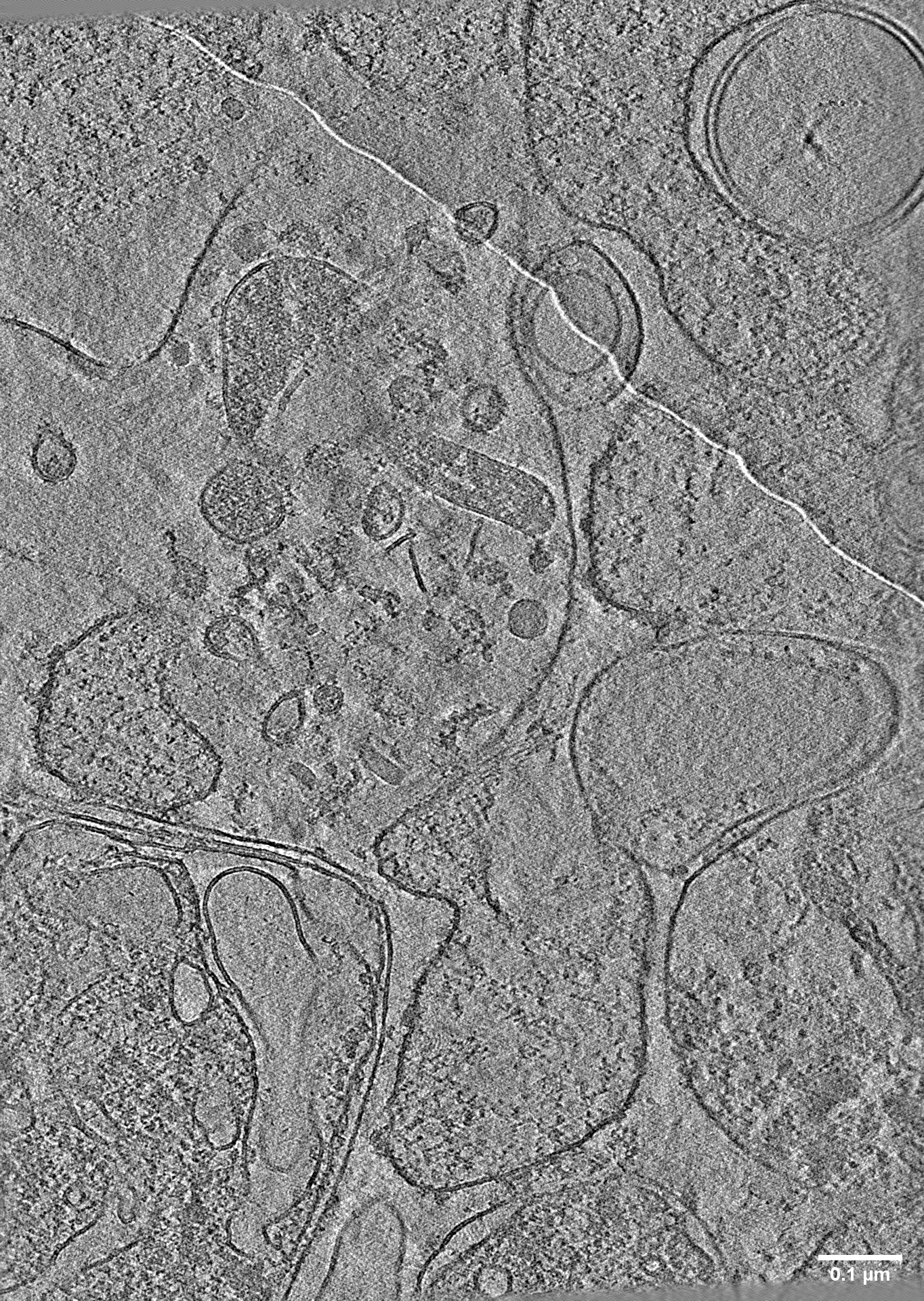

Supplement: Supplementary file 6 — Source Data for Expanded View and Appendix [file EMBJ-42-e113578-s006.zip › SupplementaryFigures_SourceData/AppendixFigureS1/PanelG/AVG10_TS_04_bin3_bandpass1.png]

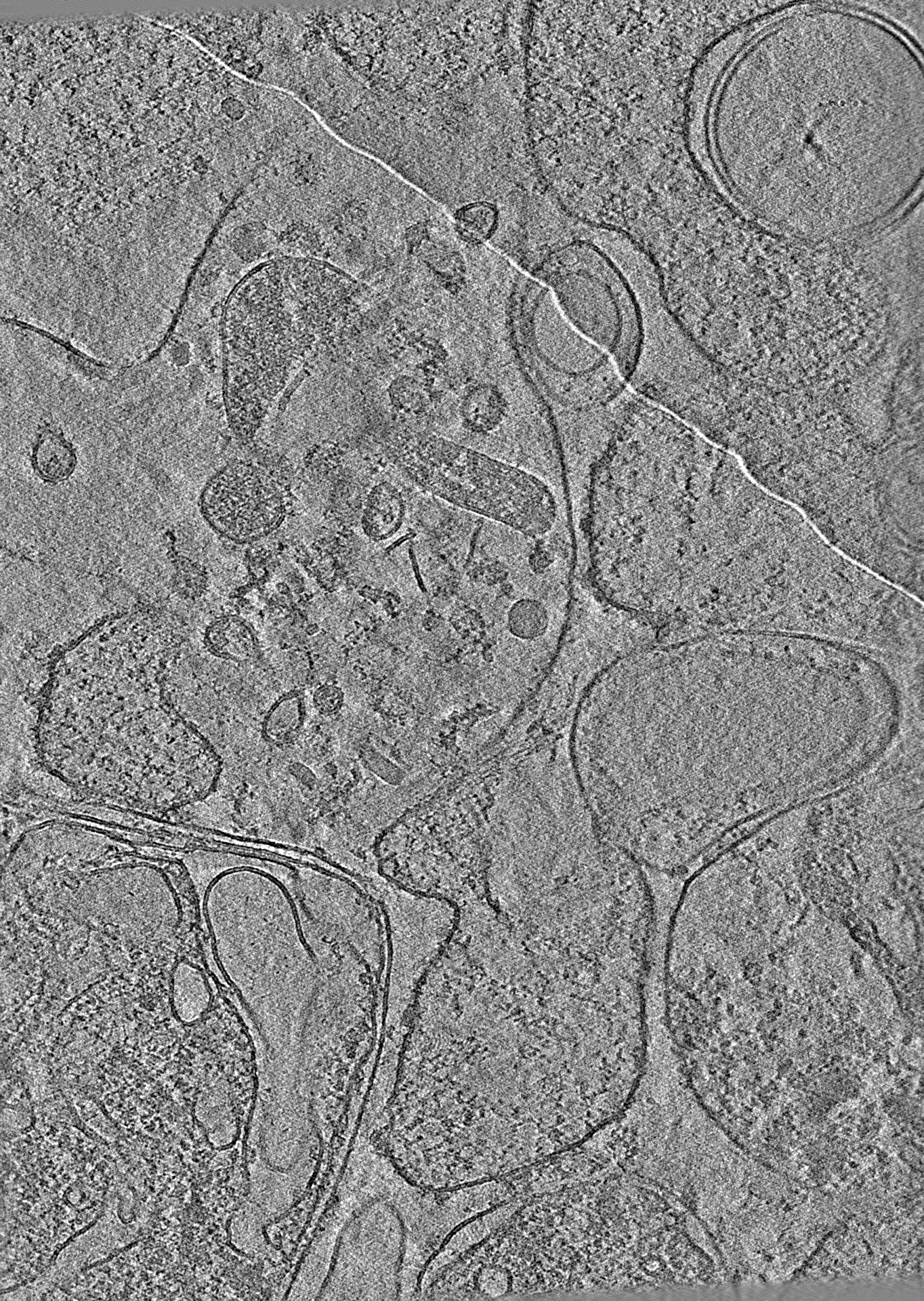

Supplement: Supplementary file 6 — Source Data for Expanded View and Appendix [file EMBJ-42-e113578-s006.zip › SupplementaryFigures_SourceData/AppendixFigureS1/PanelG/AVG10_TS_04_bin3_bandpass1.tif]

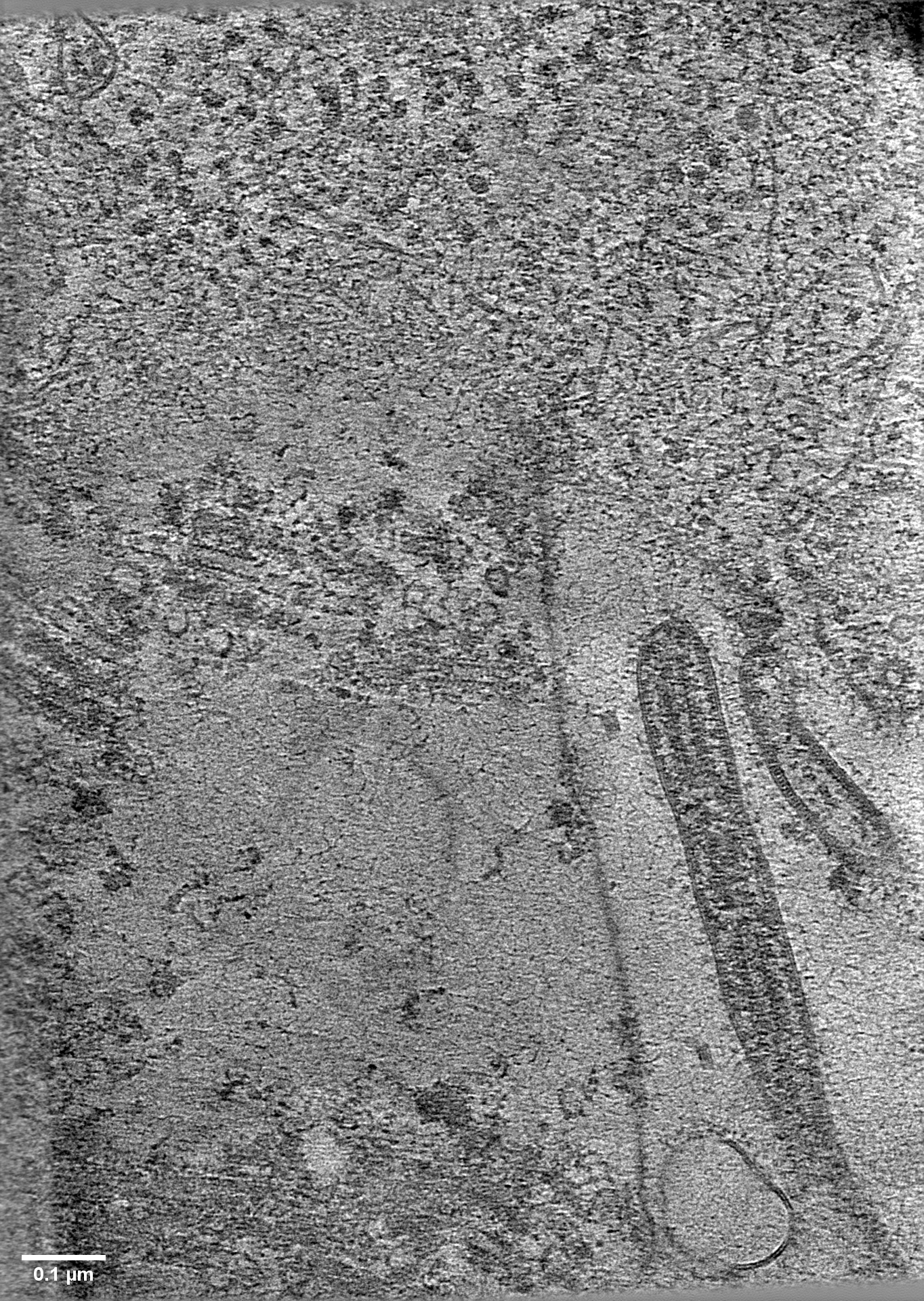

Supplement: Supplementary file 6 — Source Data for Expanded View and Appendix [file EMBJ-42-e113578-s006.zip › SupplementaryFigures_SourceData/AppendixFigureS2/PanelA/AVG10_TS_14_binX_bandpass1.png]

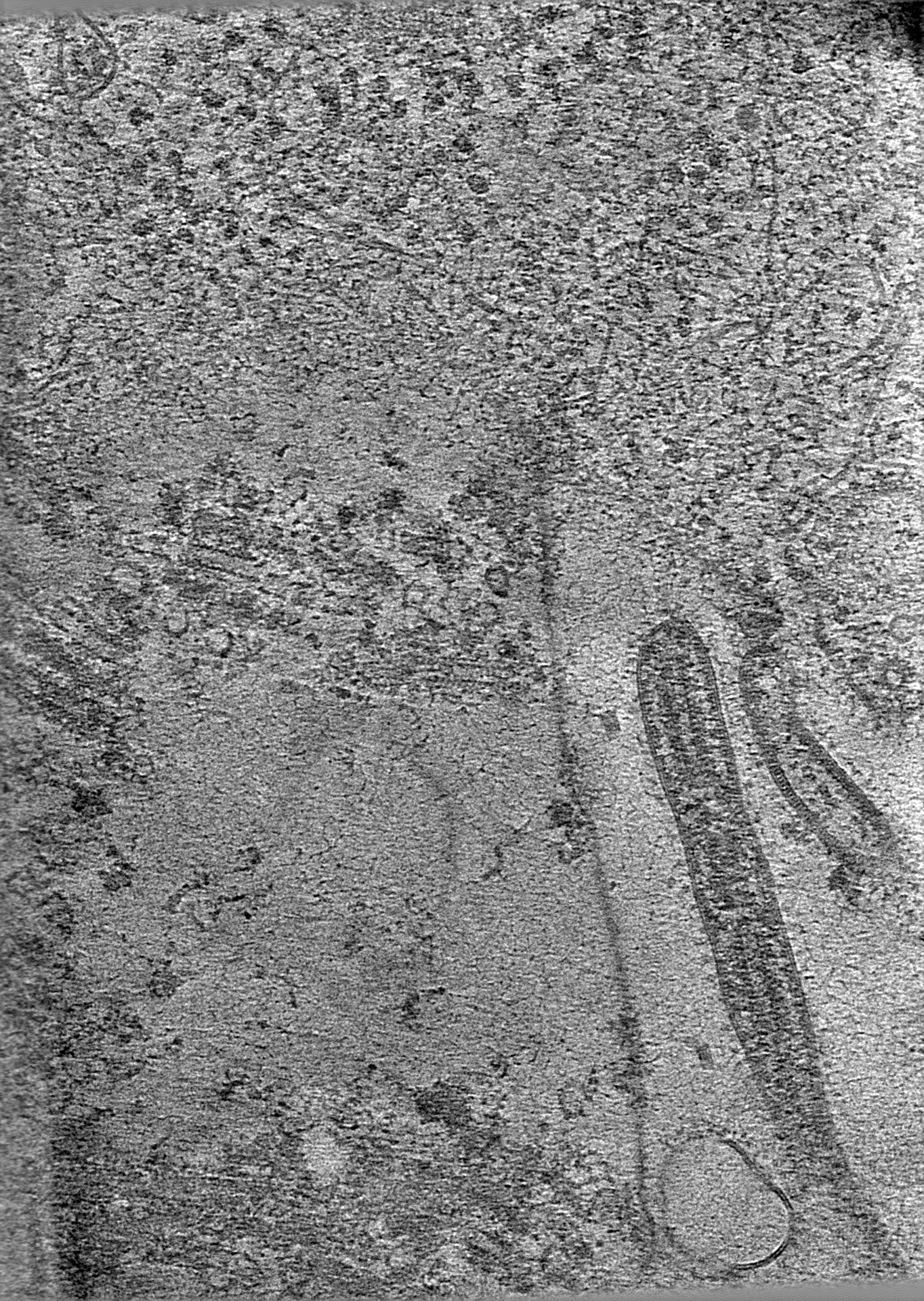

Supplement: Supplementary file 6 — Source Data for Expanded View and Appendix [file EMBJ-42-e113578-s006.zip › SupplementaryFigures_SourceData/AppendixFigureS2/PanelA/AVG10_TS_14_binX_bandpass1.tif]

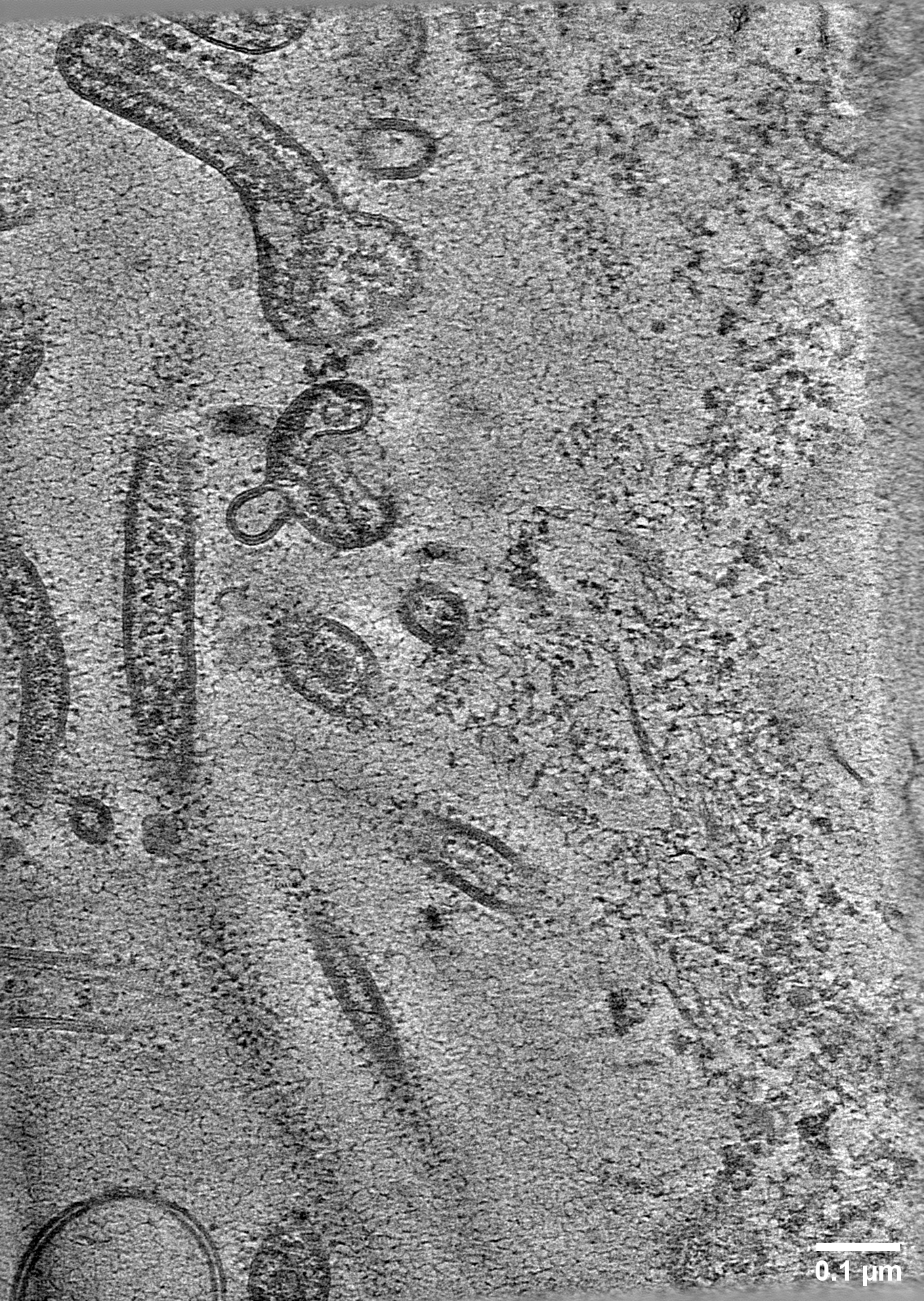

Supplement: Supplementary file 6 — Source Data for Expanded View and Appendix [file EMBJ-42-e113578-s006.zip › SupplementaryFigures_SourceData/AppendixFigureS2/PanelB/AVG10_TS_15_binX_bandpass1.png]

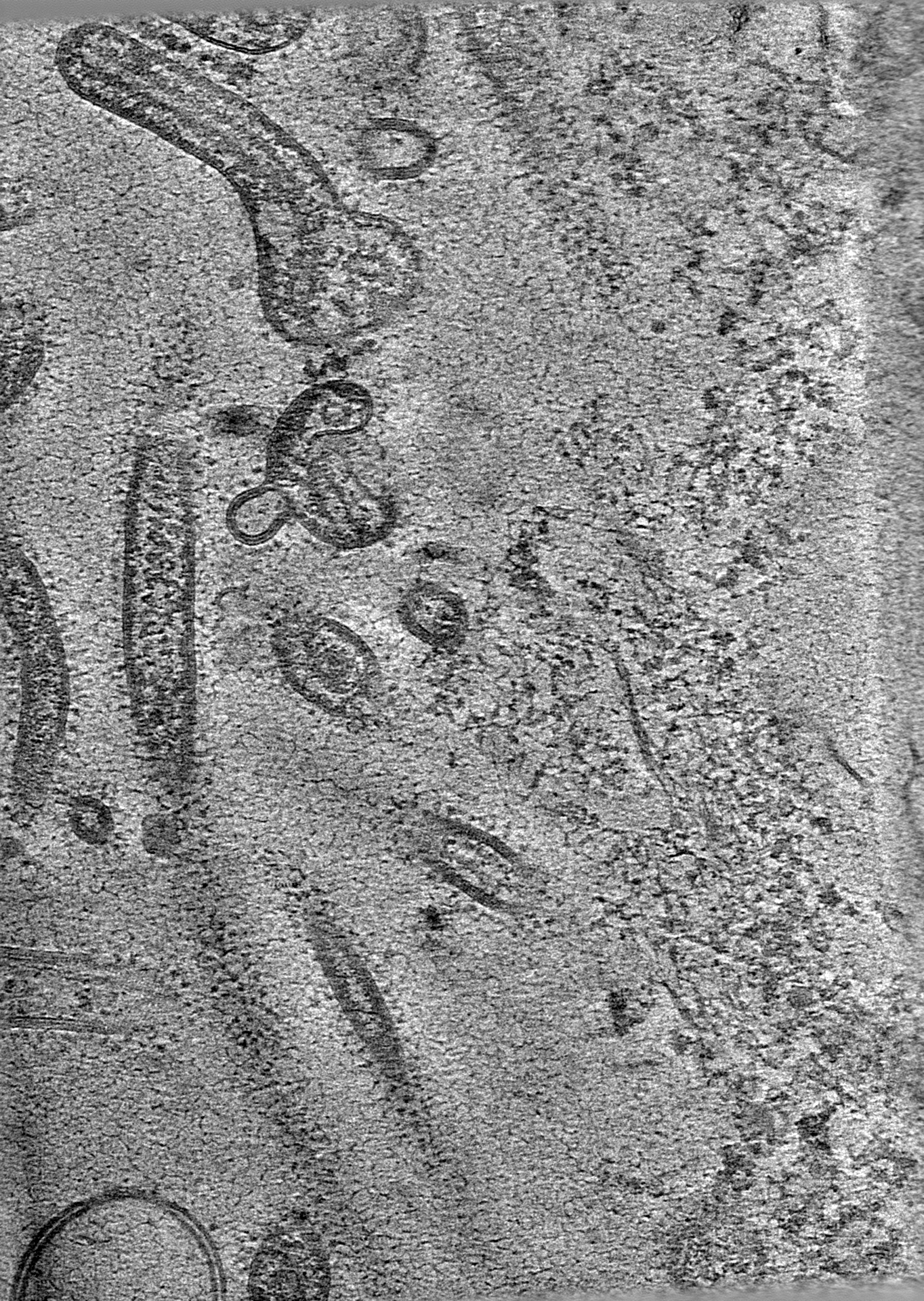

Supplement: Supplementary file 6 — Source Data for Expanded View and Appendix [file EMBJ-42-e113578-s006.zip › SupplementaryFigures_SourceData/AppendixFigureS2/PanelB/AVG10_TS_15_binX_bandpass1.tif]

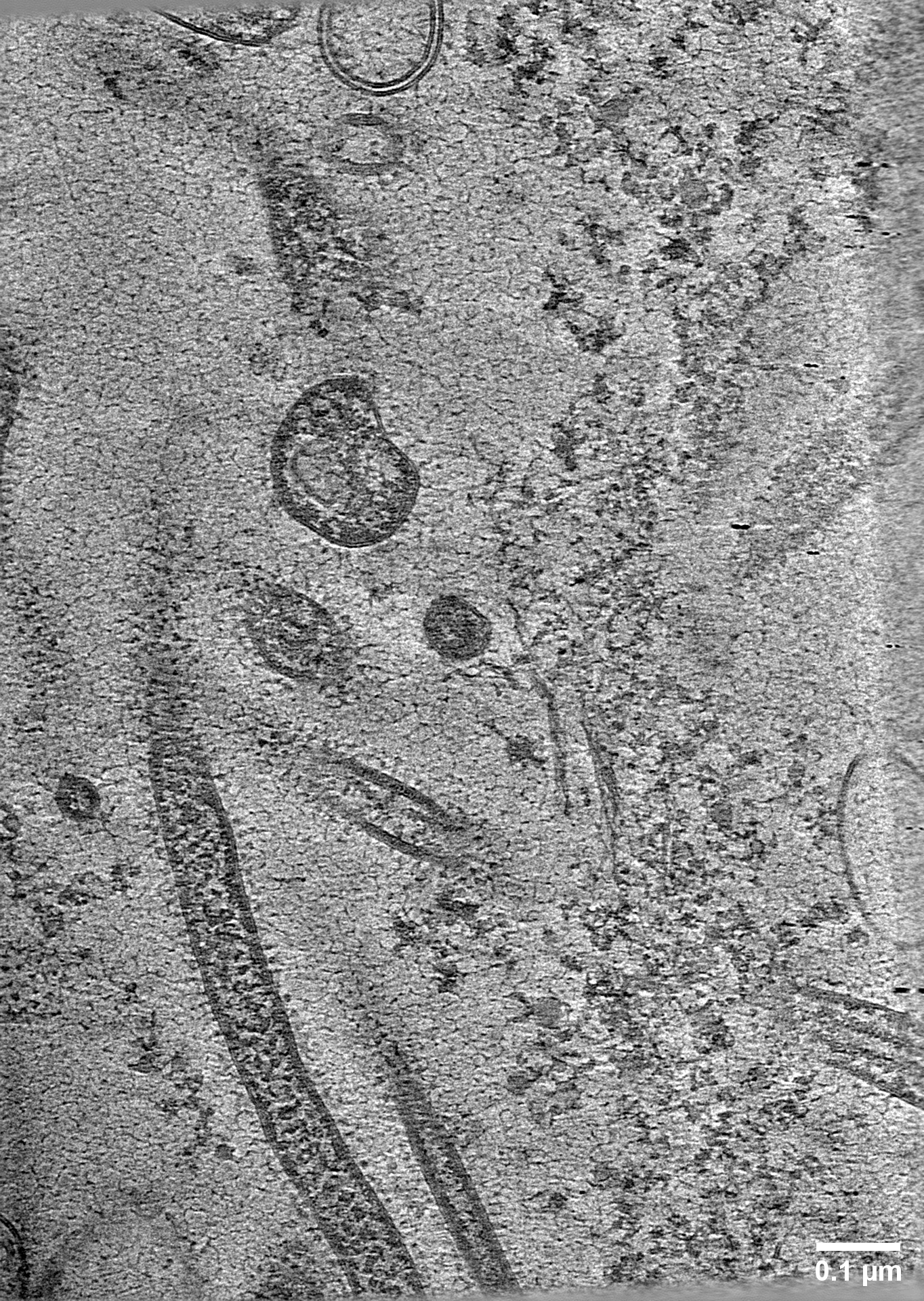

Supplement: Supplementary file 6 — Source Data for Expanded View and Appendix [file EMBJ-42-e113578-s006.zip › SupplementaryFigures_SourceData/AppendixFigureS2/PanelC/AVG10_TS_15_binX_bandpass1_2.png]

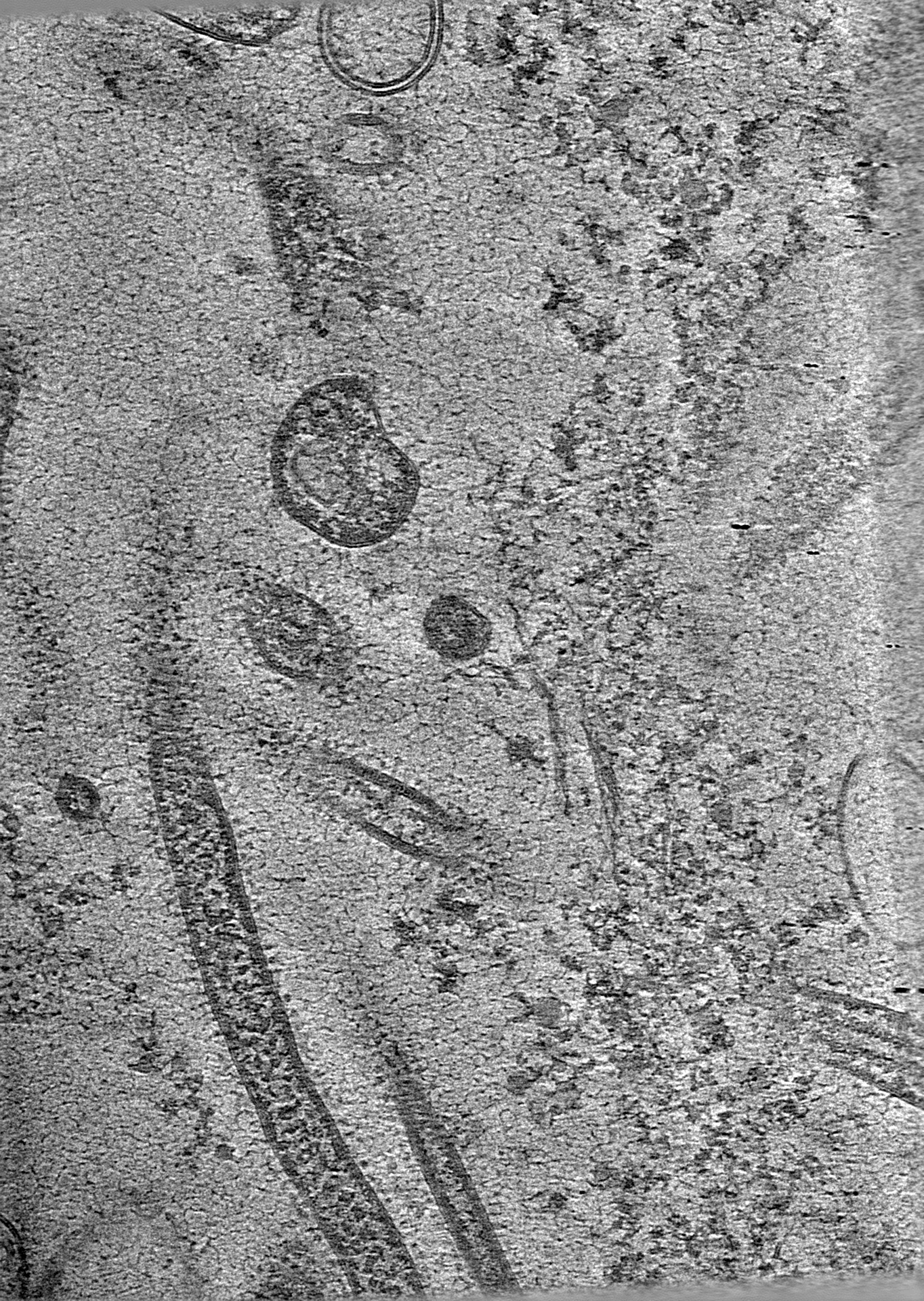

Supplement: Supplementary file 6 — Source Data for Expanded View and Appendix [file EMBJ-42-e113578-s006.zip › SupplementaryFigures_SourceData/AppendixFigureS2/PanelC/AVG10_TS_15_binX_bandpass1_2.tif]

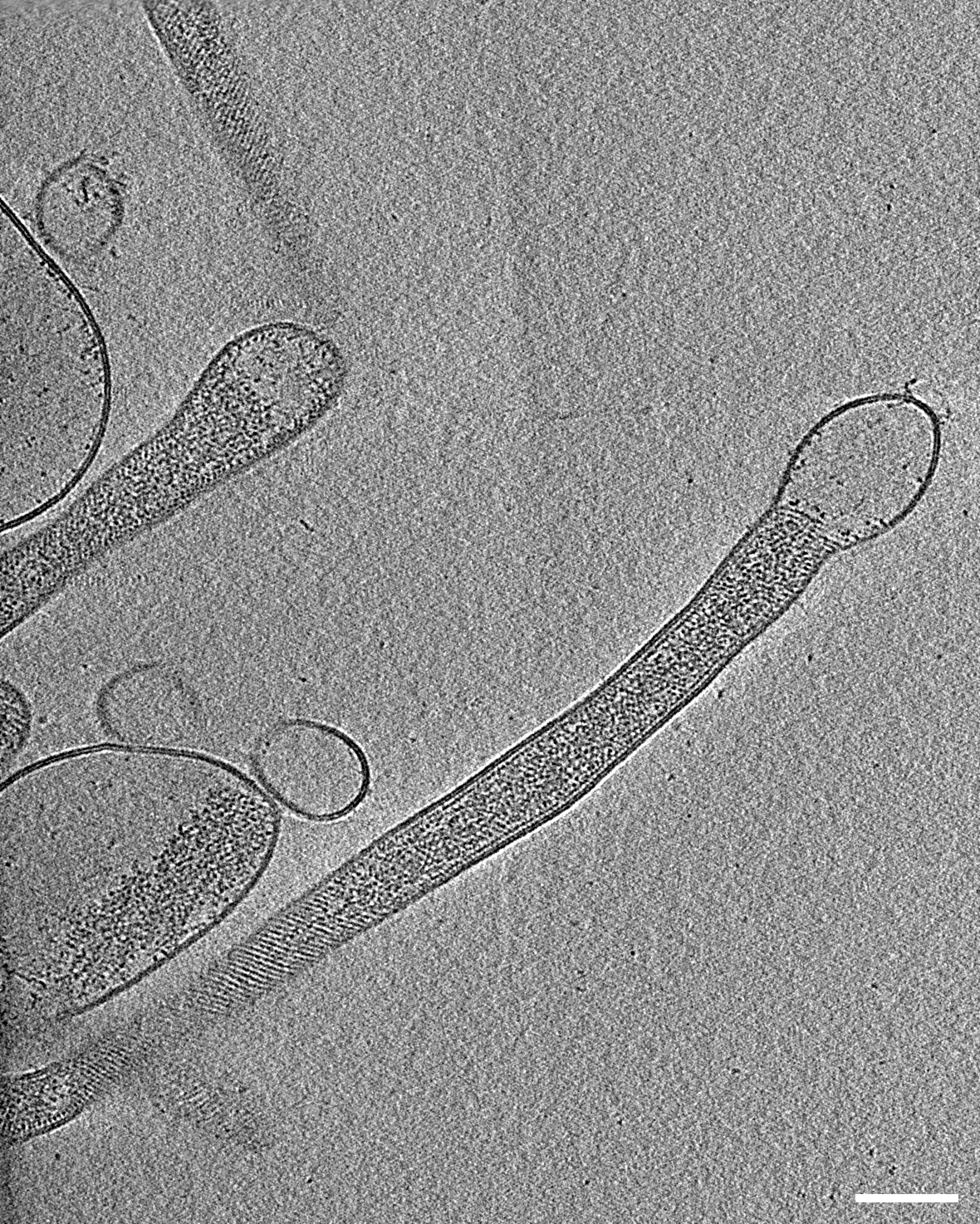

Supplement: Supplementary file 6 — Source Data for Expanded View and Appendix [file EMBJ-42-e113578-s006.zip › SupplementaryFigures_SourceData/AppendixFigureS3/PanelA/VP40/AVG10_MMM10_TS02_bin3_bandpass1_100nm.png]

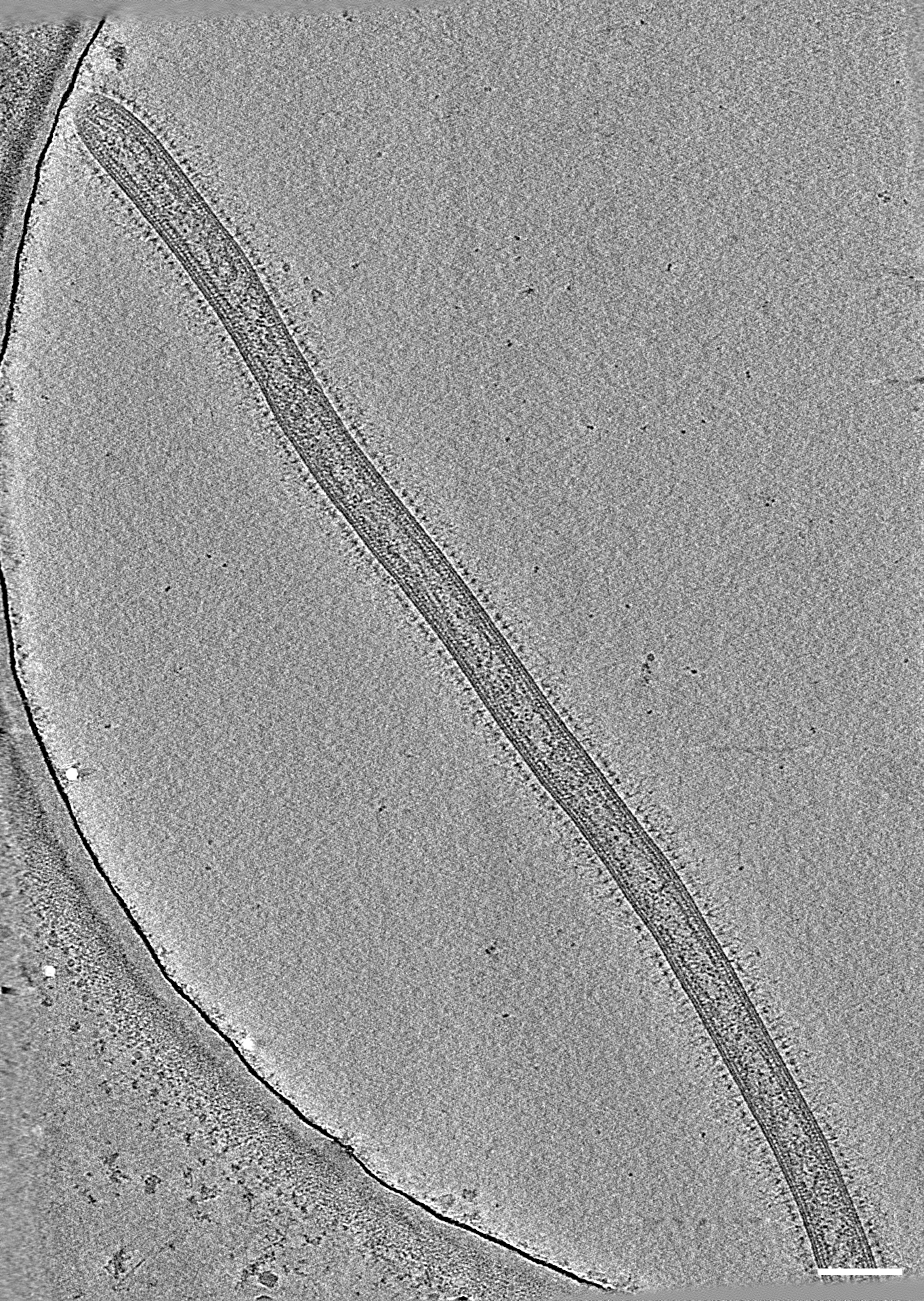

Supplement: Supplementary file 6 — Source Data for Expanded View and Appendix [file EMBJ-42-e113578-s006.zip › SupplementaryFigures_SourceData/AppendixFigureS3/PanelA/VP40-GP/AVG10_TS01_bin3._bandpass1_100nmpng.png]

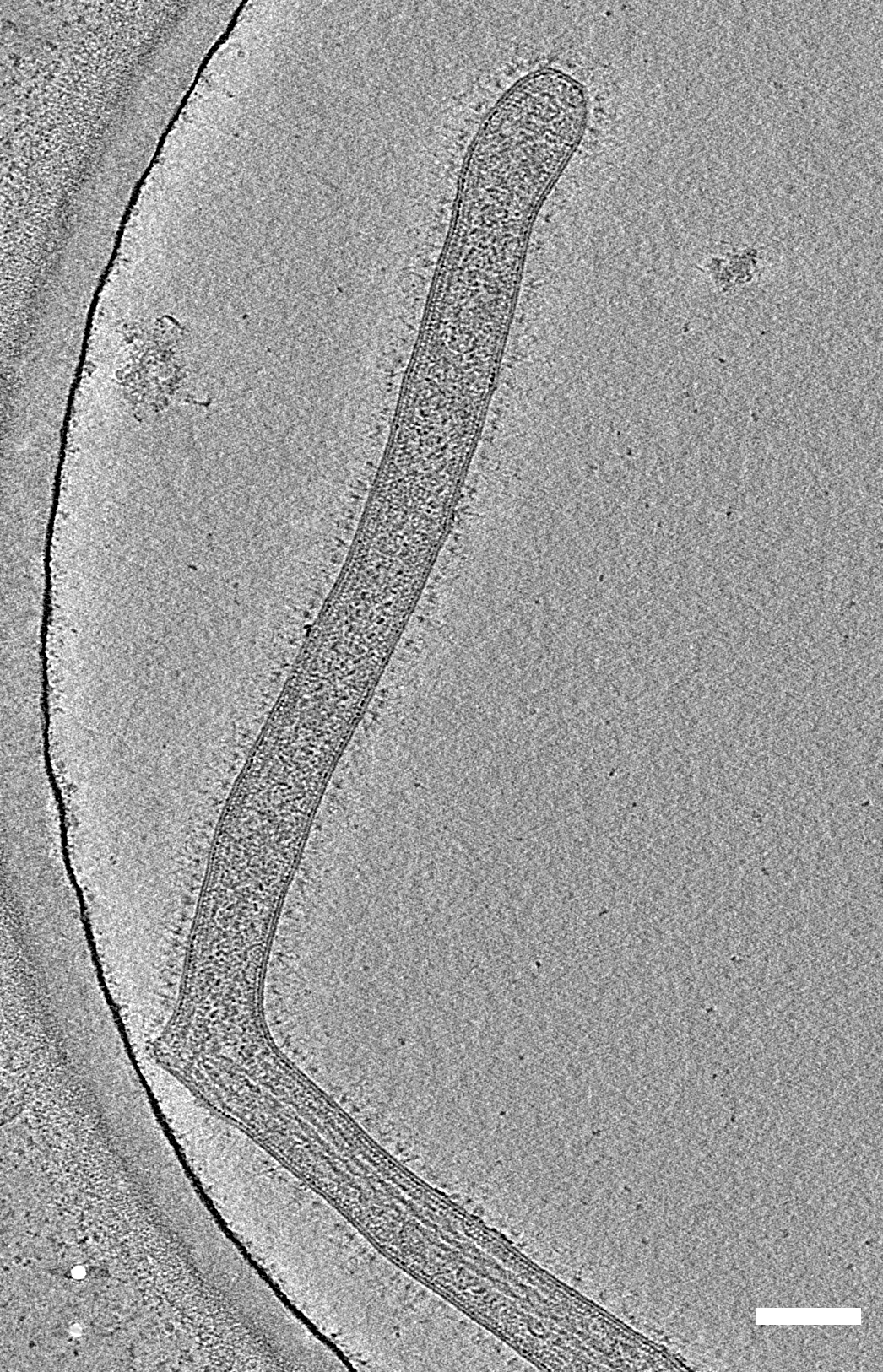

Supplement: Supplementary file 6 — Source Data for Expanded View and Appendix [file EMBJ-42-e113578-s006.zip › SupplementaryFigures_SourceData/AppendixFigureS3/PanelA/VP40-GP-NC/AVG10_TS_01_bin3_bandpass1_100nm.png]

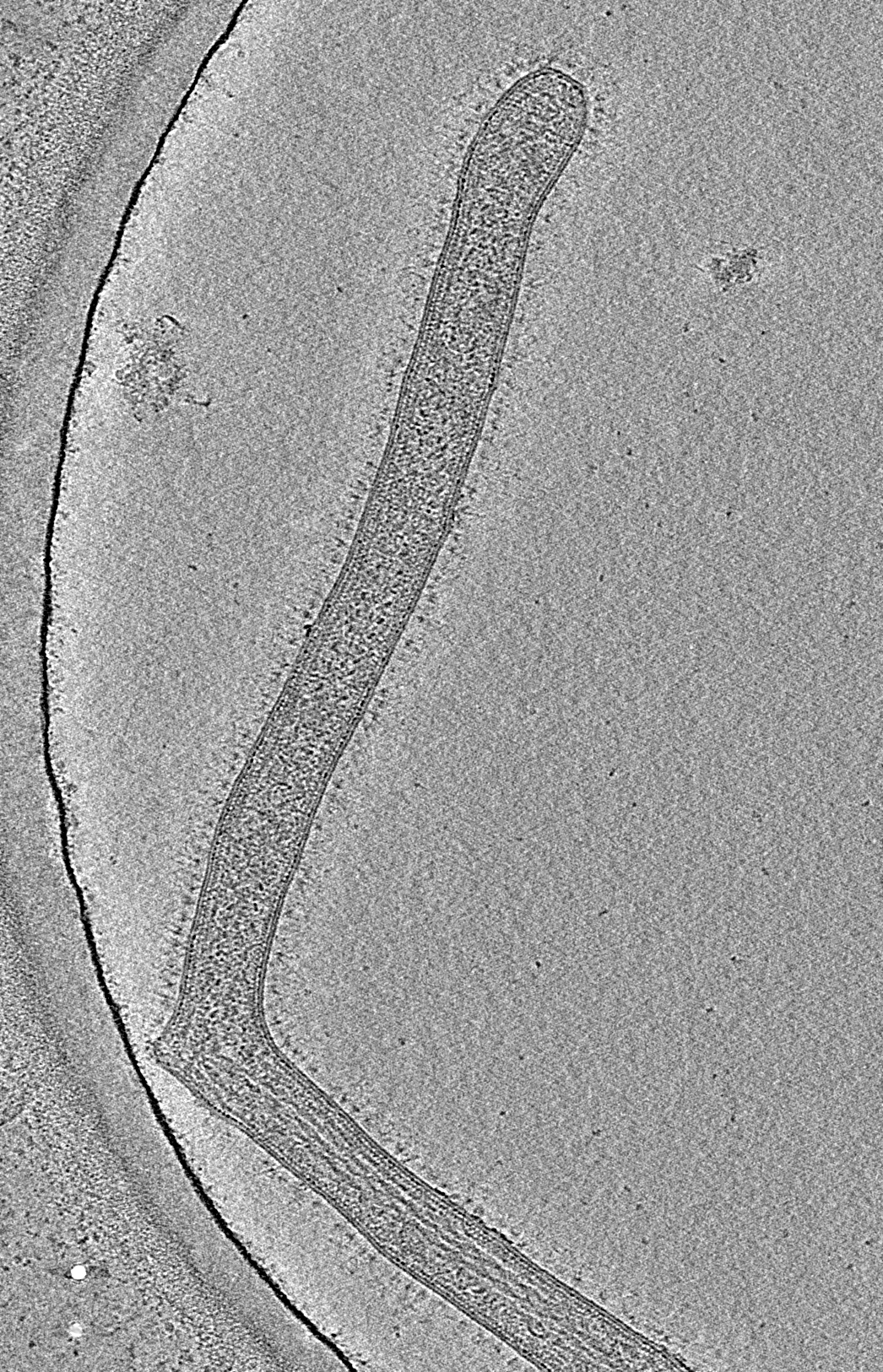

Supplement: Supplementary file 6 — Source Data for Expanded View and Appendix [file EMBJ-42-e113578-s006.zip › SupplementaryFigures_SourceData/AppendixFigureS3/PanelA/VP40-GP-NC/AVG10_TS_01_bin3_bandpass1_100nm.tif]

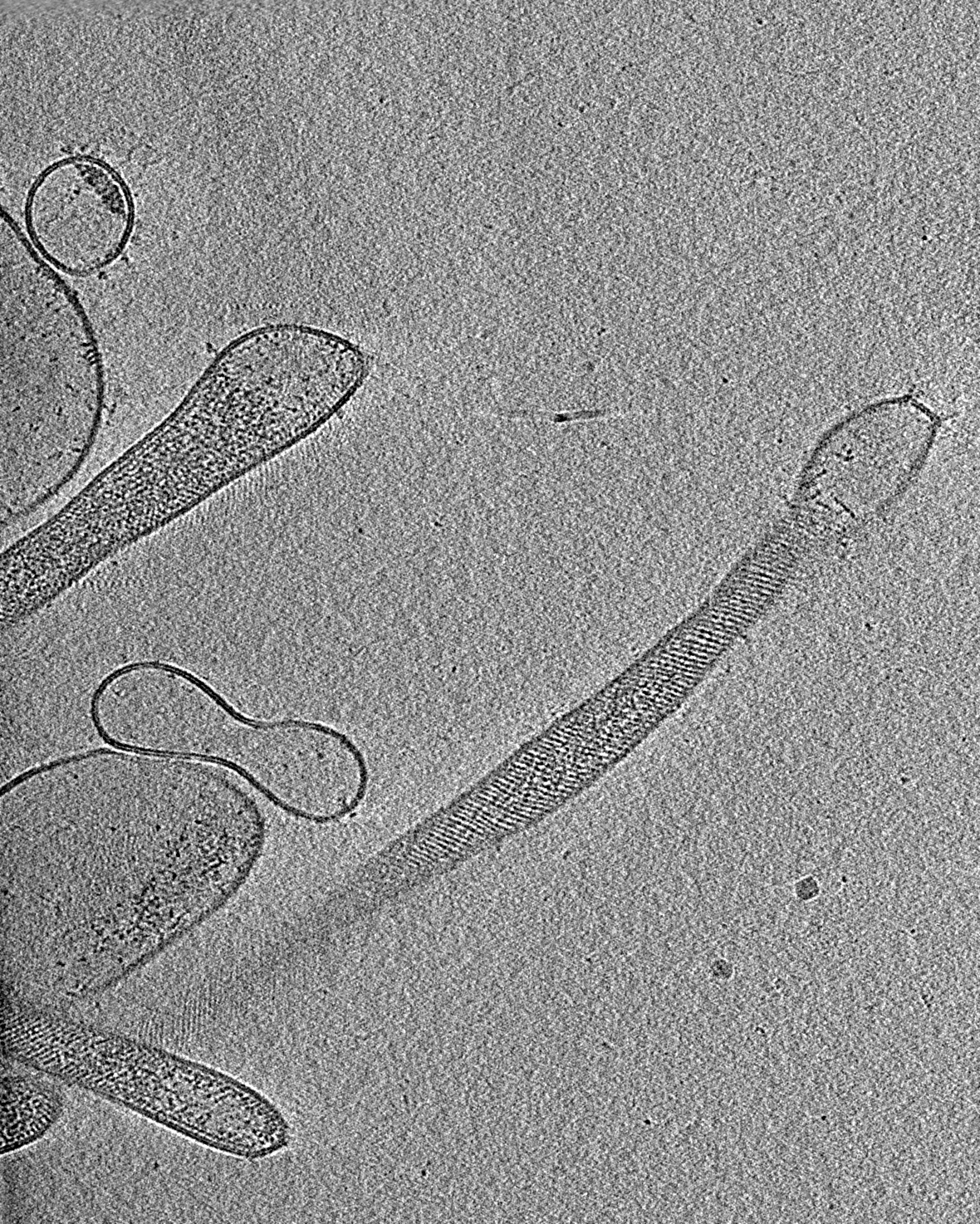

Supplement: Supplementary file 6 — Source Data for Expanded View and Appendix [file EMBJ-42-e113578-s006.zip › SupplementaryFigures_SourceData/AppendixFigureS3/PanelB/VP40/AVG10_MMM10_TS02_bin3_bandpass1_top.png]

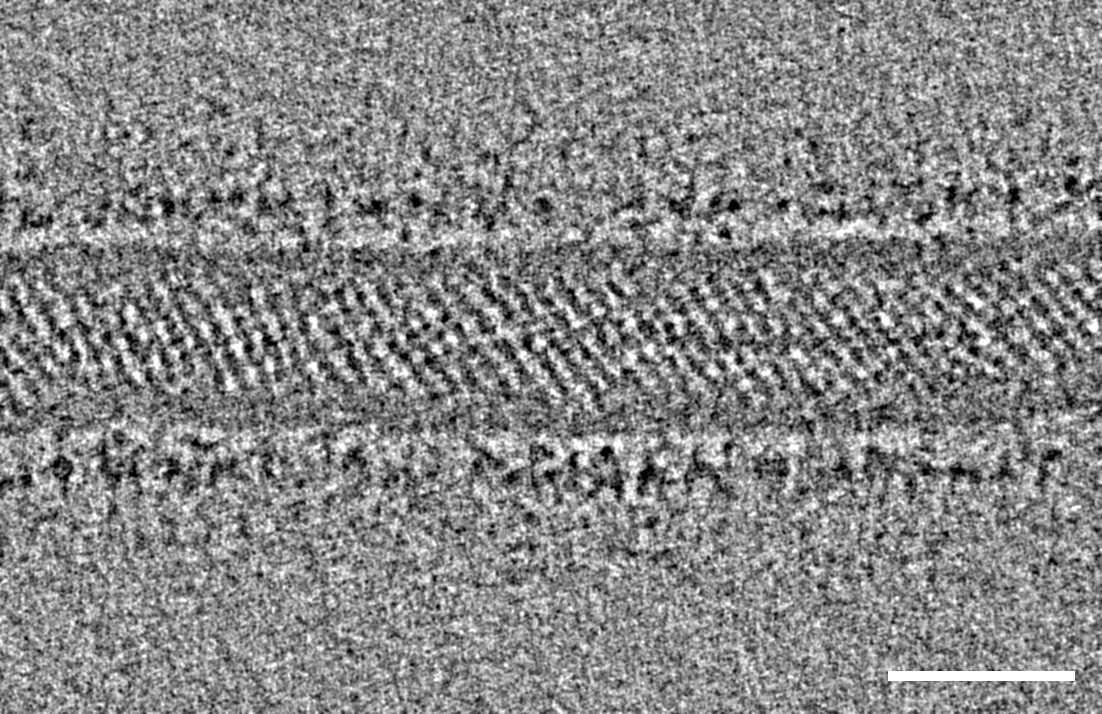

Supplement: Supplementary file 6 — Source Data for Expanded View and Appendix [file EMBJ-42-e113578-s006.zip › SupplementaryFigures_SourceData/AppendixFigureS3/PanelB/VP40-GP/AVG10_TS01_bin1_bandpass2_50nm_top.png]

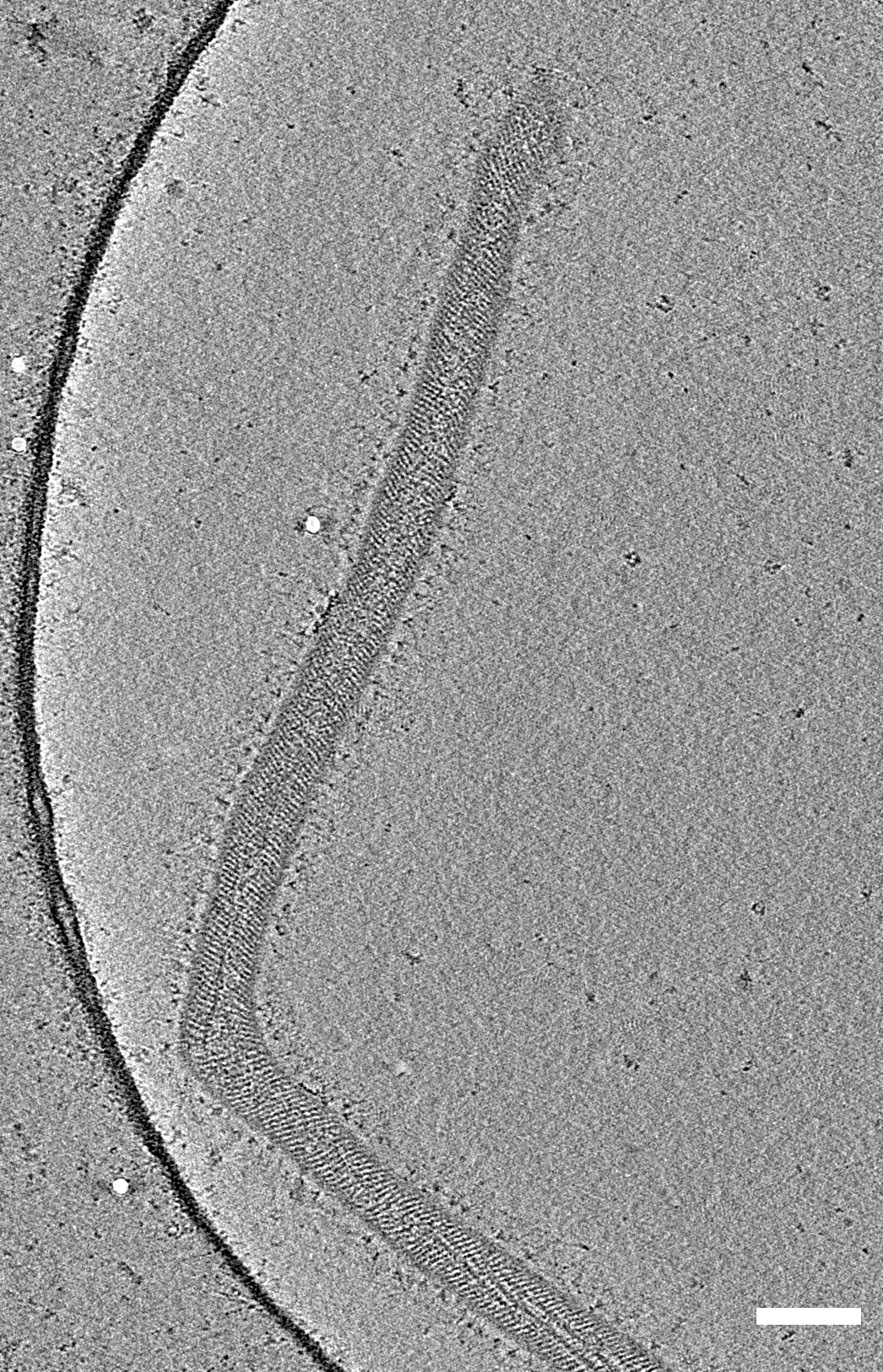

Supplement: Supplementary file 6 — Source Data for Expanded View and Appendix [file EMBJ-42-e113578-s006.zip › SupplementaryFigures_SourceData/AppendixFigureS3/PanelB/VP40-GP-NC/AVG10_TS_01_bin3_bandpass1_100nm_top.png]

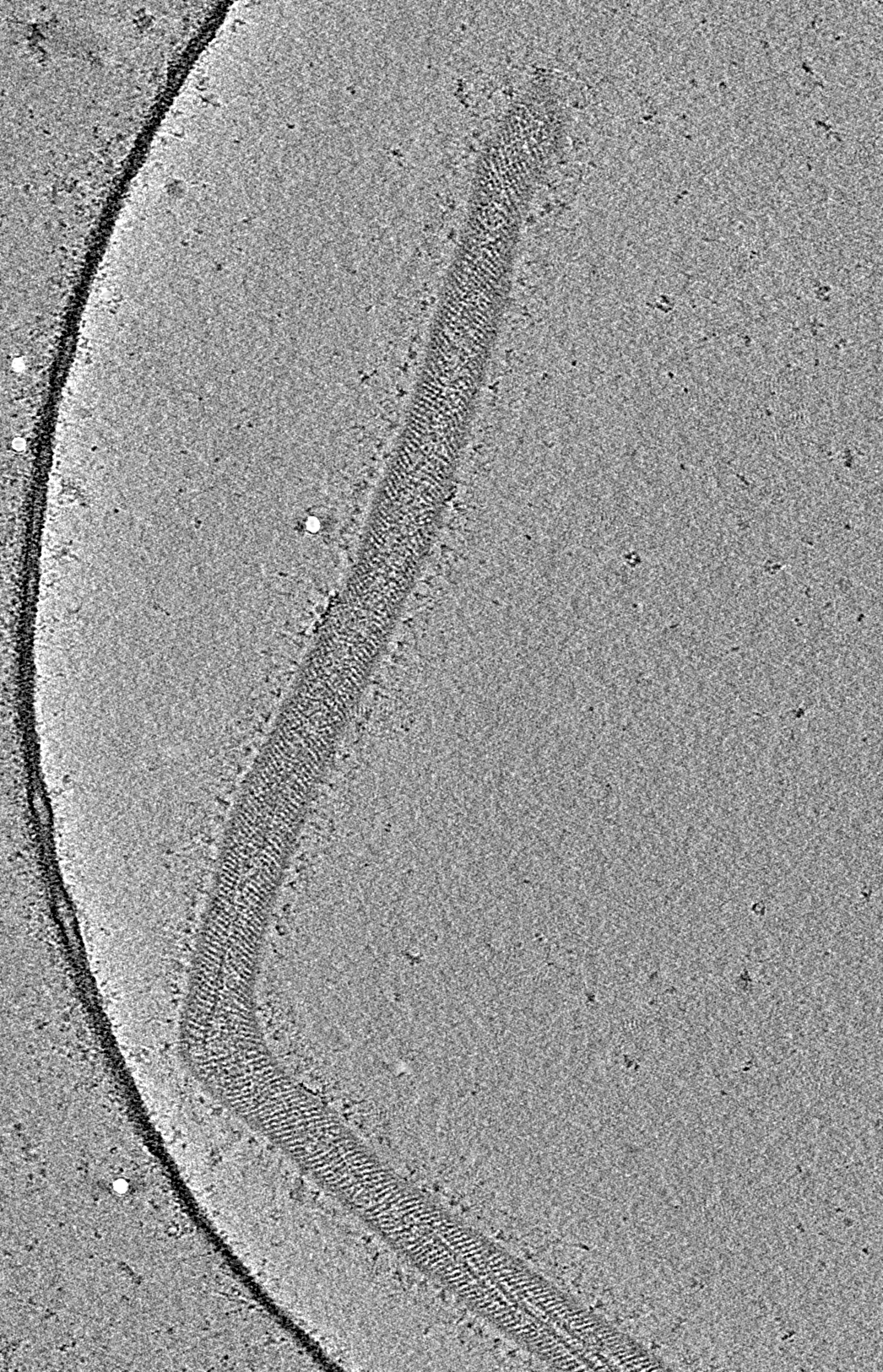

Supplement: Supplementary file 6 — Source Data for Expanded View and Appendix [file EMBJ-42-e113578-s006.zip › SupplementaryFigures_SourceData/AppendixFigureS3/PanelB/VP40-GP-NC/AVG10_TS_01_bin3_bandpass1_100nm_top.tif]

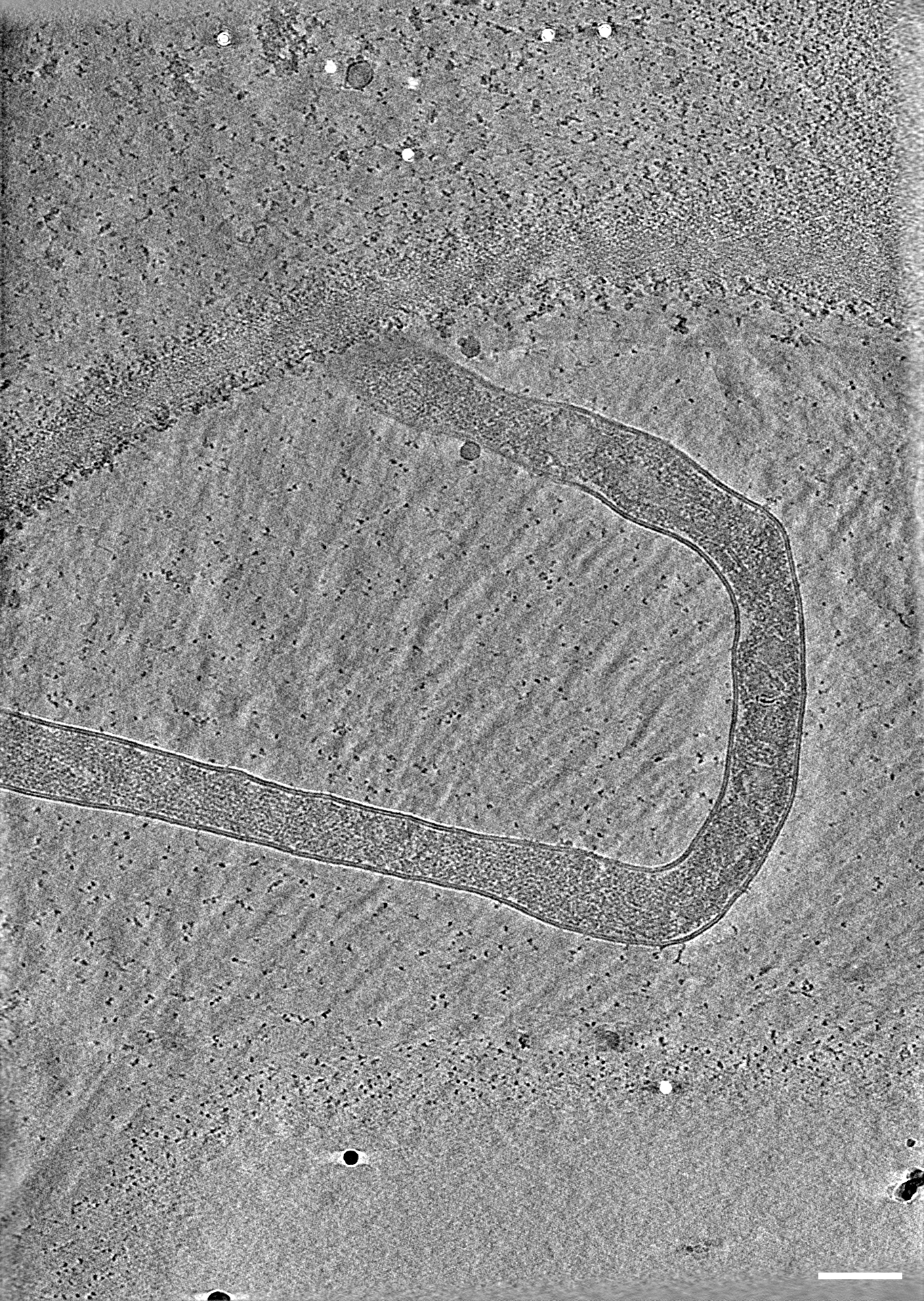

Supplement: Supplementary file 6 — Source Data for Expanded View and Appendix [file EMBJ-42-e113578-s006.zip › SupplementaryFigures_SourceData/AppendixFigureS3/PanelC/VP40/AVG10_M5_TS02_bin3_bandpass1_100nm_2.png]

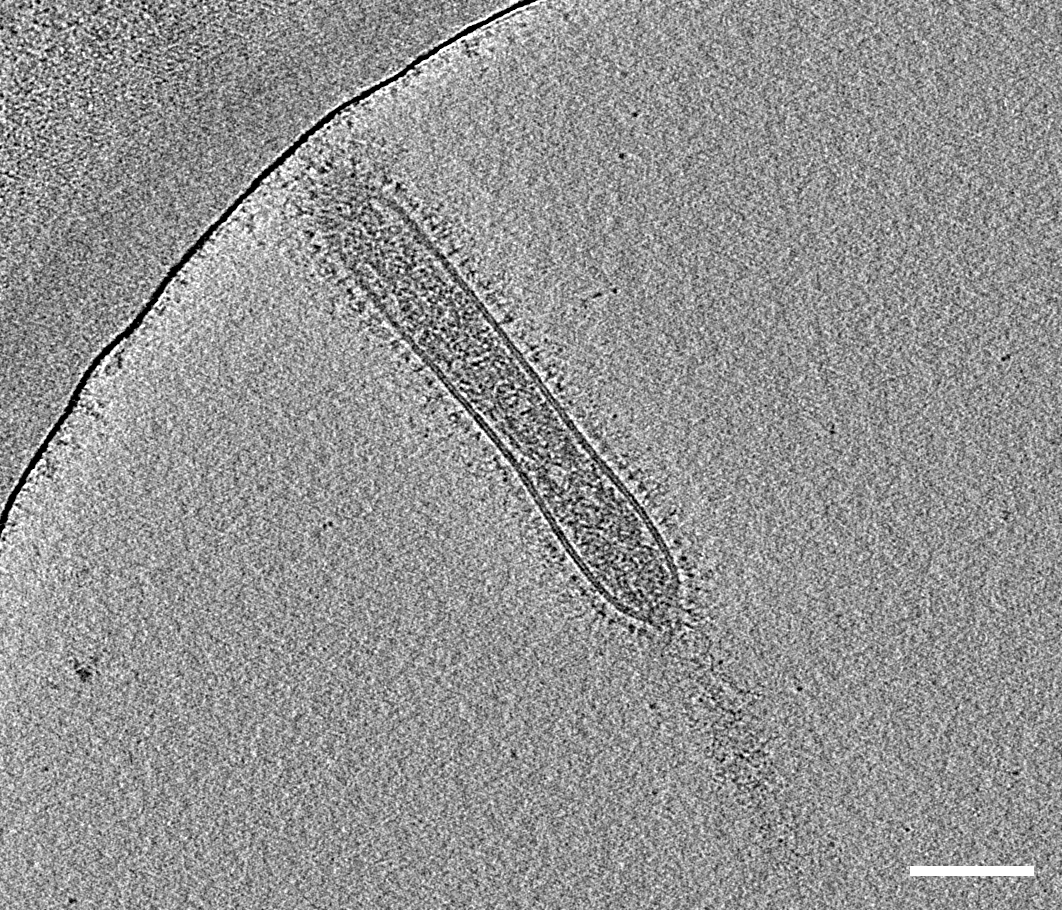

Supplement: Supplementary file 6 — Source Data for Expanded View and Appendix [file EMBJ-42-e113578-s006.zip › SupplementaryFigures_SourceData/AppendixFigureS3/PanelC/VP40-GP/AVG10_TS05_bin3_bandpass1_100nm.png]

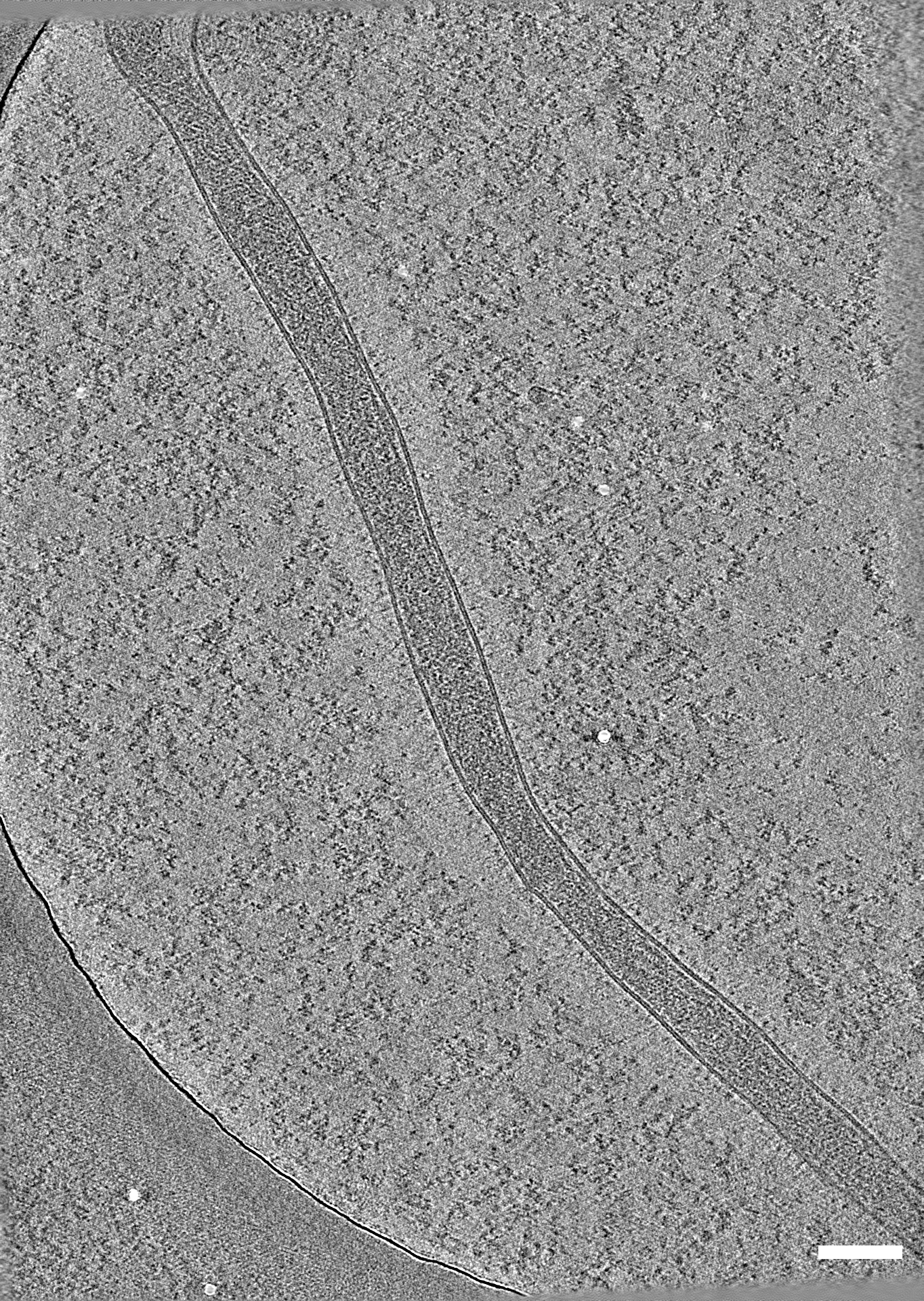

Supplement: Supplementary file 6 — Source Data for Expanded View and Appendix [file EMBJ-42-e113578-s006.zip › SupplementaryFigures_SourceData/AppendixFigureS3/PanelC/VP40-GP-NC/AVG10_TS_07_bin3_bandpass1_100nm.png]

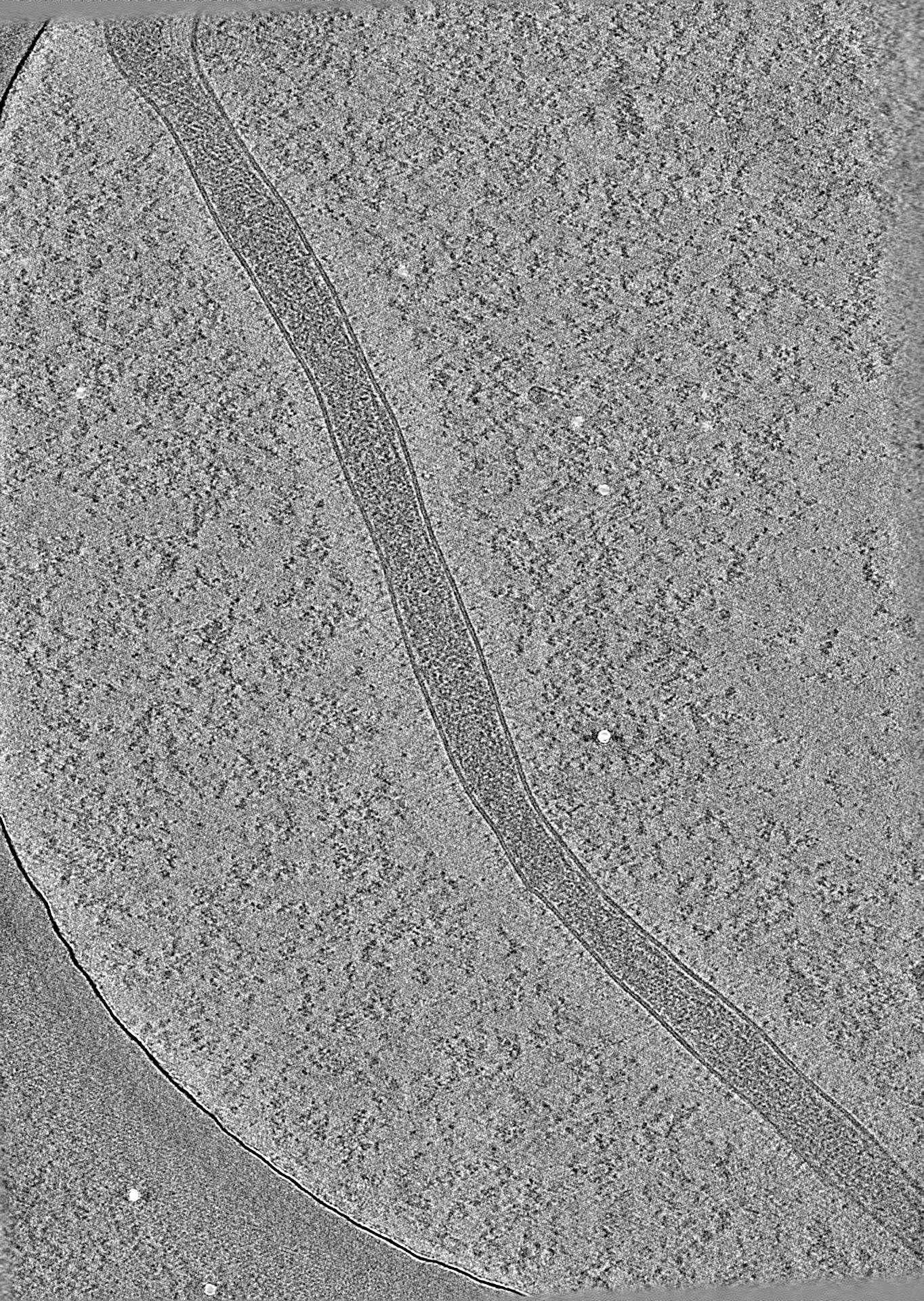

Supplement: Supplementary file 6 — Source Data for Expanded View and Appendix [file EMBJ-42-e113578-s006.zip › SupplementaryFigures_SourceData/AppendixFigureS3/PanelC/VP40-GP-NC/AVG10_TS_07_bin3_bandpass1_100nm.tif]

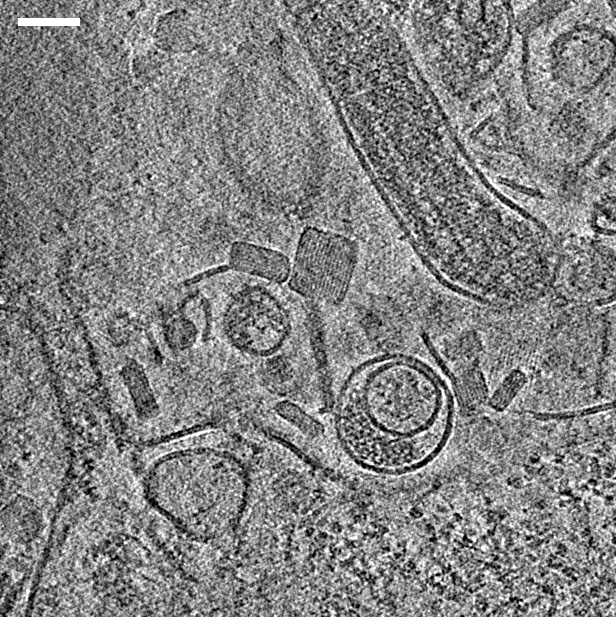

Supplement: Supplementary file 6 — Source Data for Expanded View and Appendix [file EMBJ-42-e113578-s006.zip › SupplementaryFigures_SourceData/FigureEV1/PanelA/AVG10_TS_02_bin3_50nm_overview2.png]

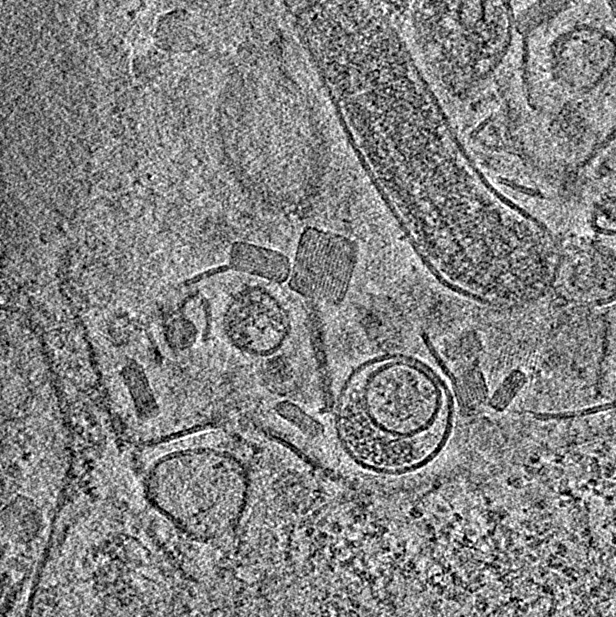

Supplement: Supplementary file 6 — Source Data for Expanded View and Appendix [file EMBJ-42-e113578-s006.zip › SupplementaryFigures_SourceData/FigureEV1/PanelA/AVG10_TS_02_bin3_50nm_overview2.tif]

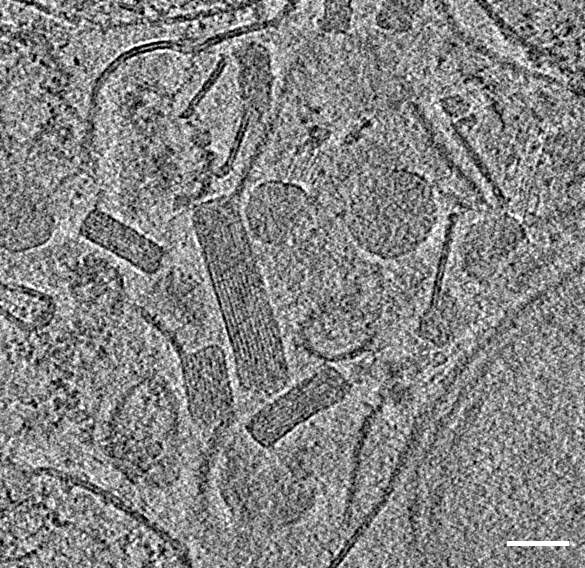

Supplement: Supplementary file 6 — Source Data for Expanded View and Appendix [file EMBJ-42-e113578-s006.zip › SupplementaryFigures_SourceData/FigureEV1/PanelB/AVG10_TS07_bin3_bandpass1_50nm_chol-crystals.png]

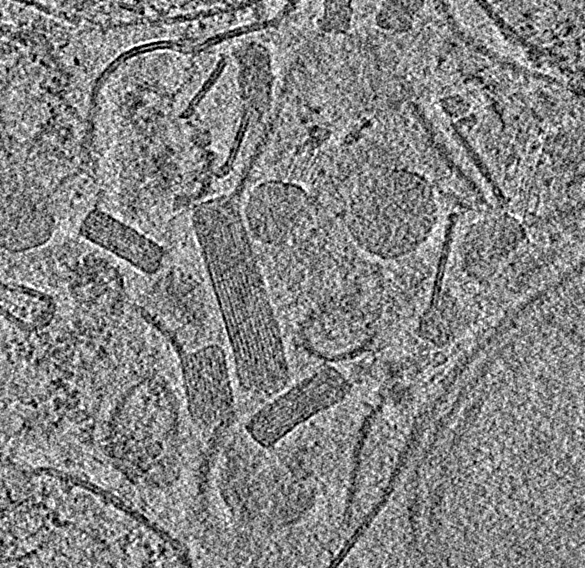

Supplement: Supplementary file 6 — Source Data for Expanded View and Appendix [file EMBJ-42-e113578-s006.zip › SupplementaryFigures_SourceData/FigureEV1/PanelB/AVG10_TS07_bin3_bandpass1_50nm_chol-crystals.tif]

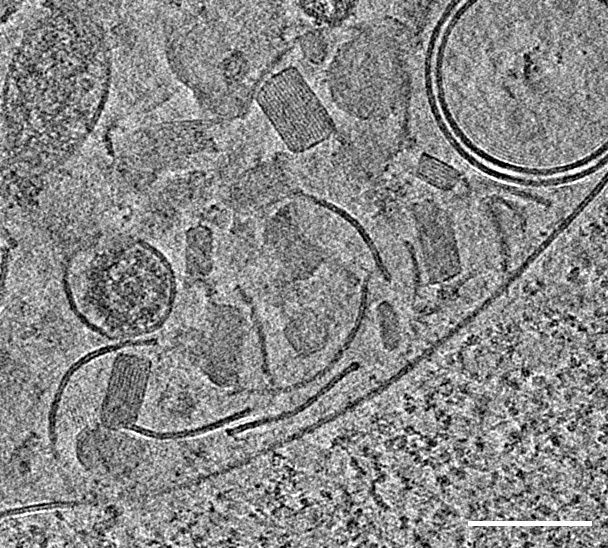

Supplement: Supplementary file 6 — Source Data for Expanded View and Appendix [file EMBJ-42-e113578-s006.zip › SupplementaryFigures_SourceData/FigureEV1/PanelC/AVG10_TS_02_bin3_100nm_overview.png]

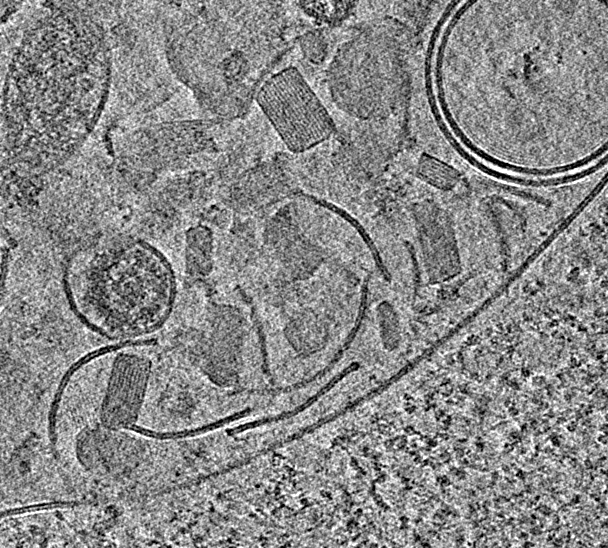

Supplement: Supplementary file 6 — Source Data for Expanded View and Appendix [file EMBJ-42-e113578-s006.zip › SupplementaryFigures_SourceData/FigureEV1/PanelC/AVG10_TS_02_bin3_100nm_overview.tif]

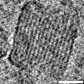

Supplement: Supplementary file 6 — Source Data for Expanded View and Appendix [file EMBJ-42-e113578-s006.zip › SupplementaryFigures_SourceData/FigureEV1/PanelD/AVG10_TS_02_bin3_20nm.png]

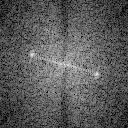

Supplement: Supplementary file 6 — Source Data for Expanded View and Appendix [file EMBJ-42-e113578-s006.zip › SupplementaryFigures_SourceData/FigureEV1/PanelE/FFT of AVG10_TS_02_bin3.png]

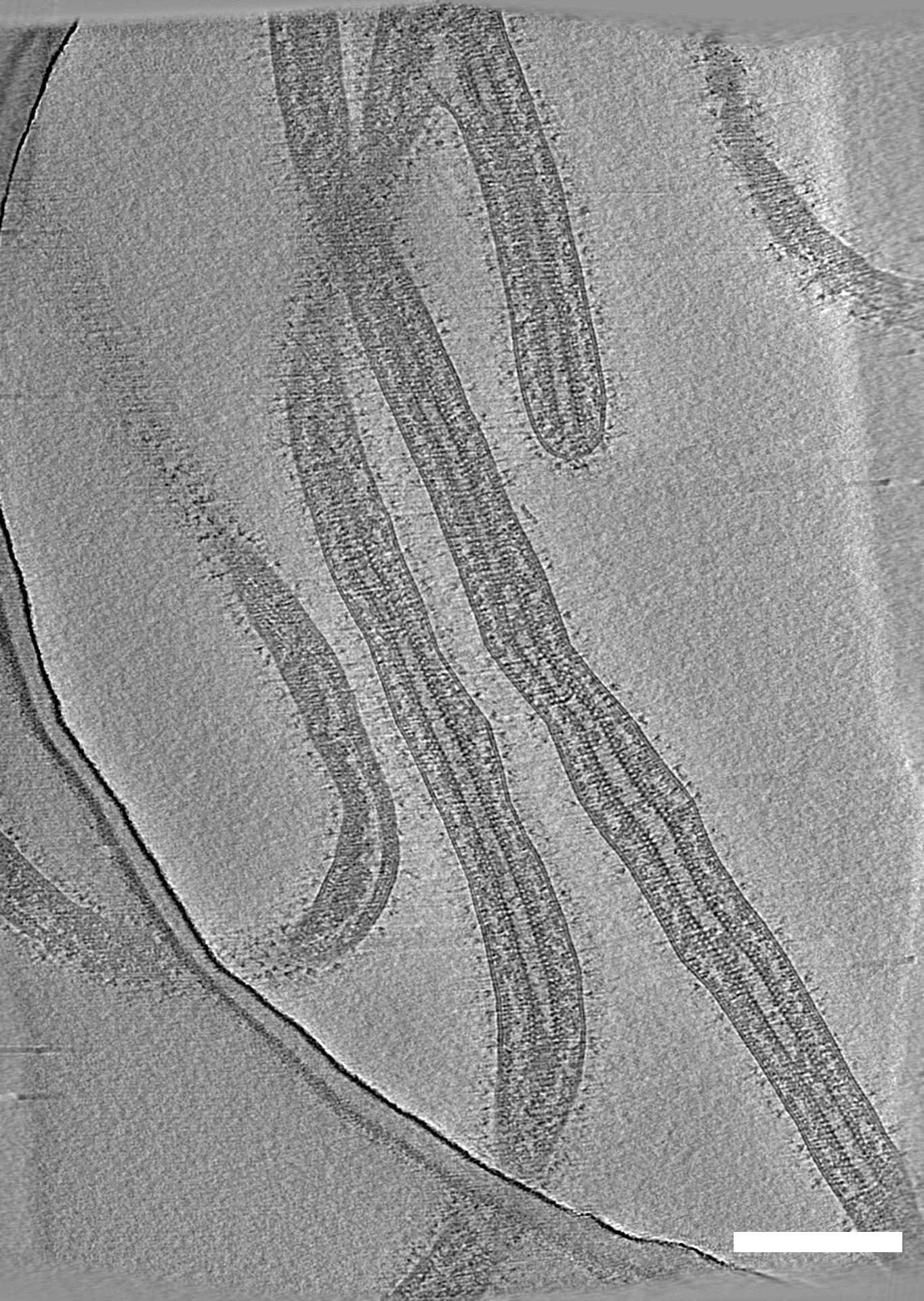

Supplement: Supplementary file 6 — Source Data for Expanded View and Appendix [file EMBJ-42-e113578-s006.zip › SupplementaryFigures_SourceData/FigureEV2/PanelA/AVG_TS_03o_200nm.png]

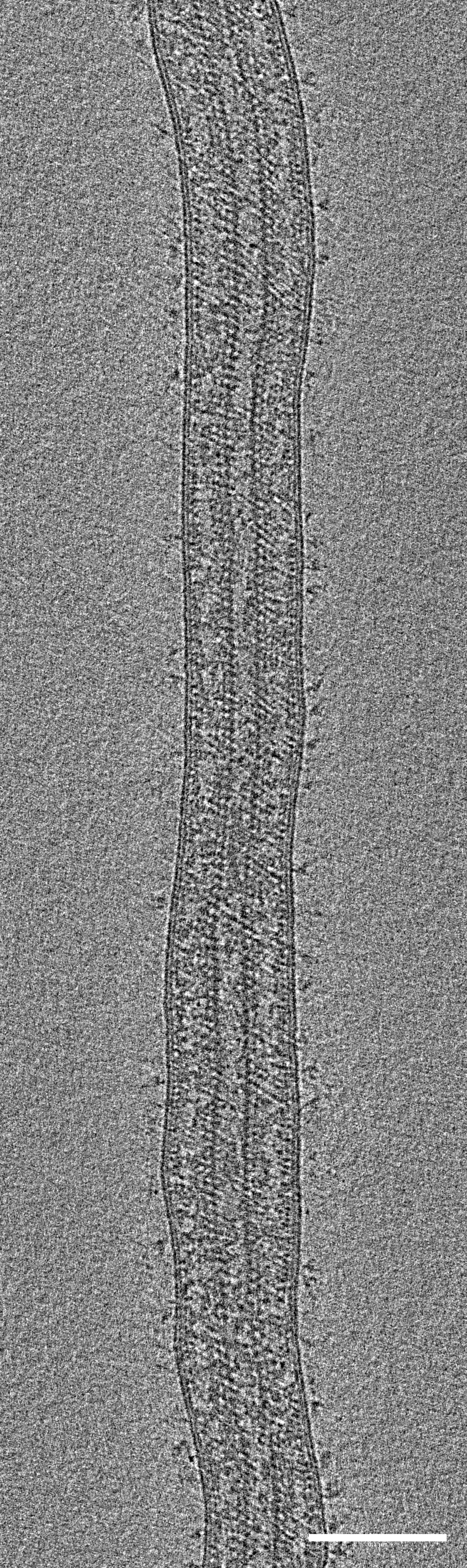

Supplement: Supplementary file 6 — Source Data for Expanded View and Appendix [file EMBJ-42-e113578-s006.zip › SupplementaryFigures_SourceData/FigureEV2/PanelB/MAX10_TS_04o_bin1_bandpass1_overview.png]

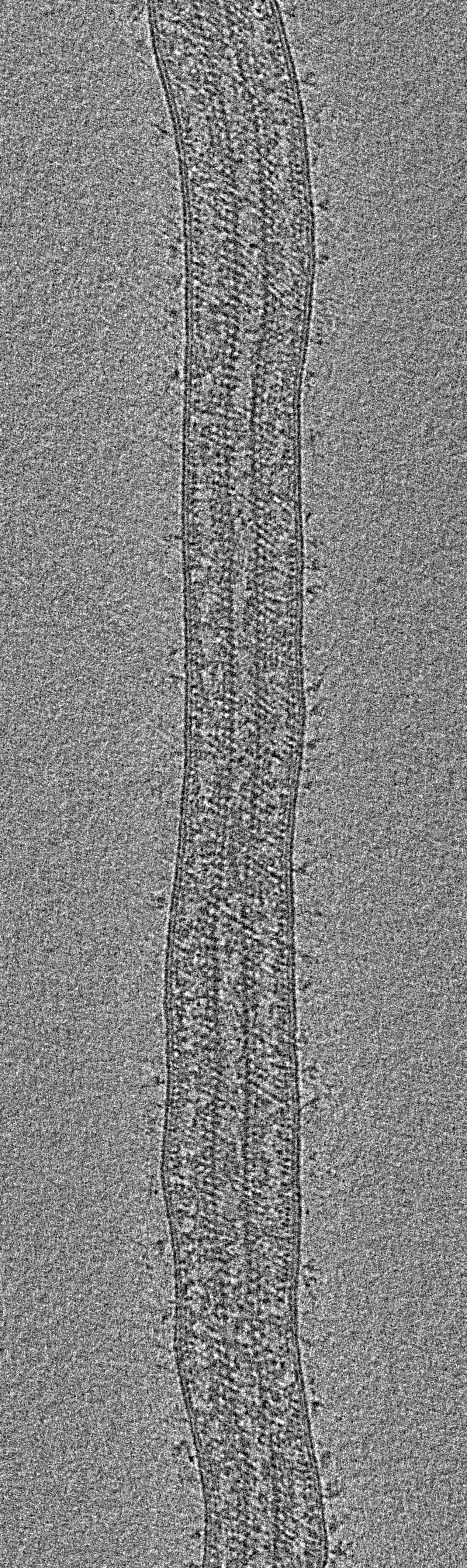

Supplement: Supplementary file 6 — Source Data for Expanded View and Appendix [file EMBJ-42-e113578-s006.zip › SupplementaryFigures_SourceData/FigureEV2/PanelB/MAX10_TS_04o_bin1_bandpass1_overview.tif]

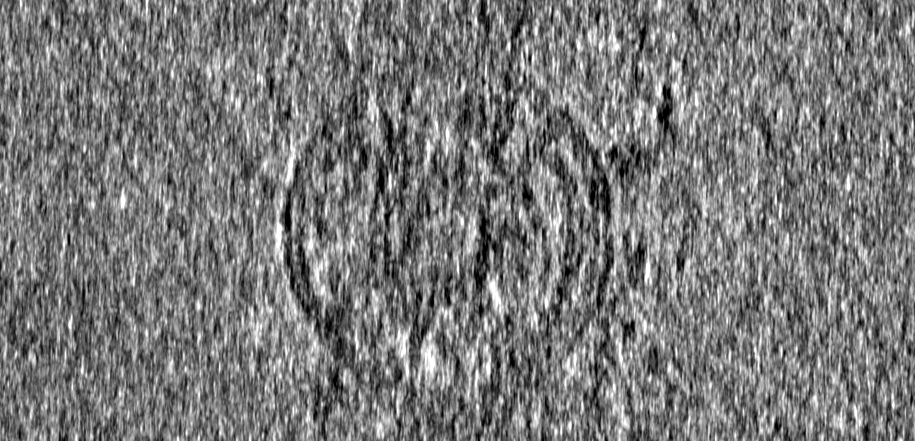

Supplement: Supplementary file 6 — Source Data for Expanded View and Appendix [file EMBJ-42-e113578-s006.zip › SupplementaryFigures_SourceData/FigureEV2/PanelC/MAX17_TS_04o_bandpass1.png]

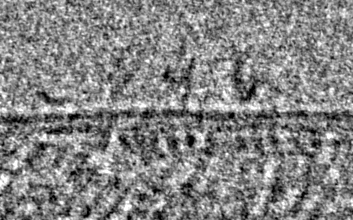

Supplement: Supplementary file 6 — Source Data for Expanded View and Appendix [file EMBJ-42-e113578-s006.zip › SupplementaryFigures_SourceData/FigureEV2/PanelD/MAX10_TS_04o_bandpass1.png]

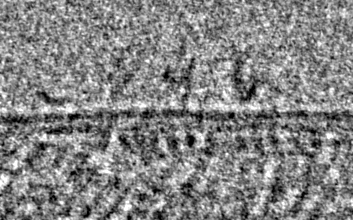

Supplement: Supplementary file 6 — Source Data for Expanded View and Appendix [file EMBJ-42-e113578-s006.zip › SupplementaryFigures_SourceData/FigureEV2/PanelD/MAX10_TS_04o_bandpass1_20nm.tif]

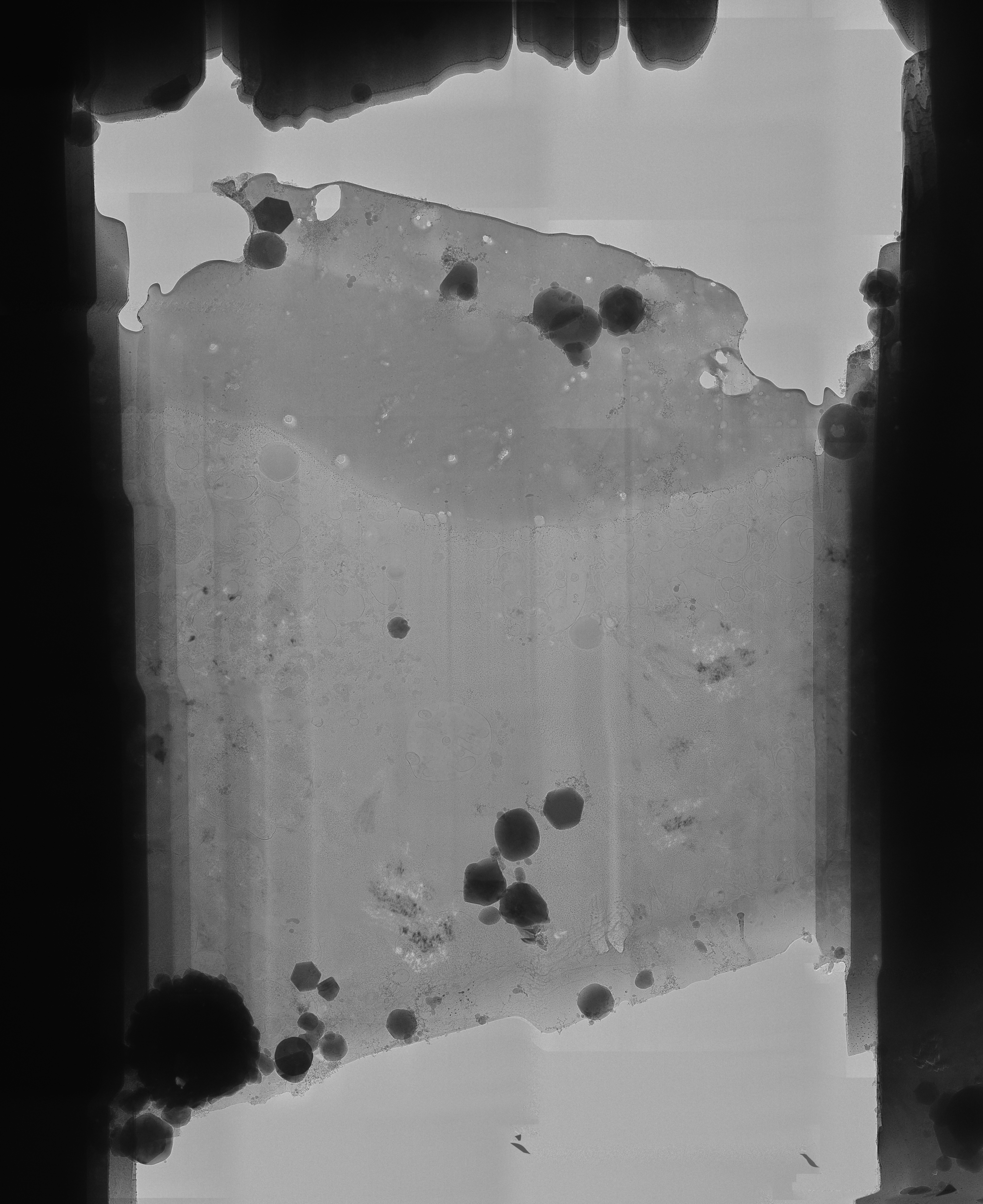

Supplement: Supplementary file 6 — Source Data for Expanded View and Appendix [file EMBJ-42-e113578-s006.zip › SupplementaryFigures_SourceData/FigureEV3/PanelA/MMM7_stitched_gauss3_bin2.png]

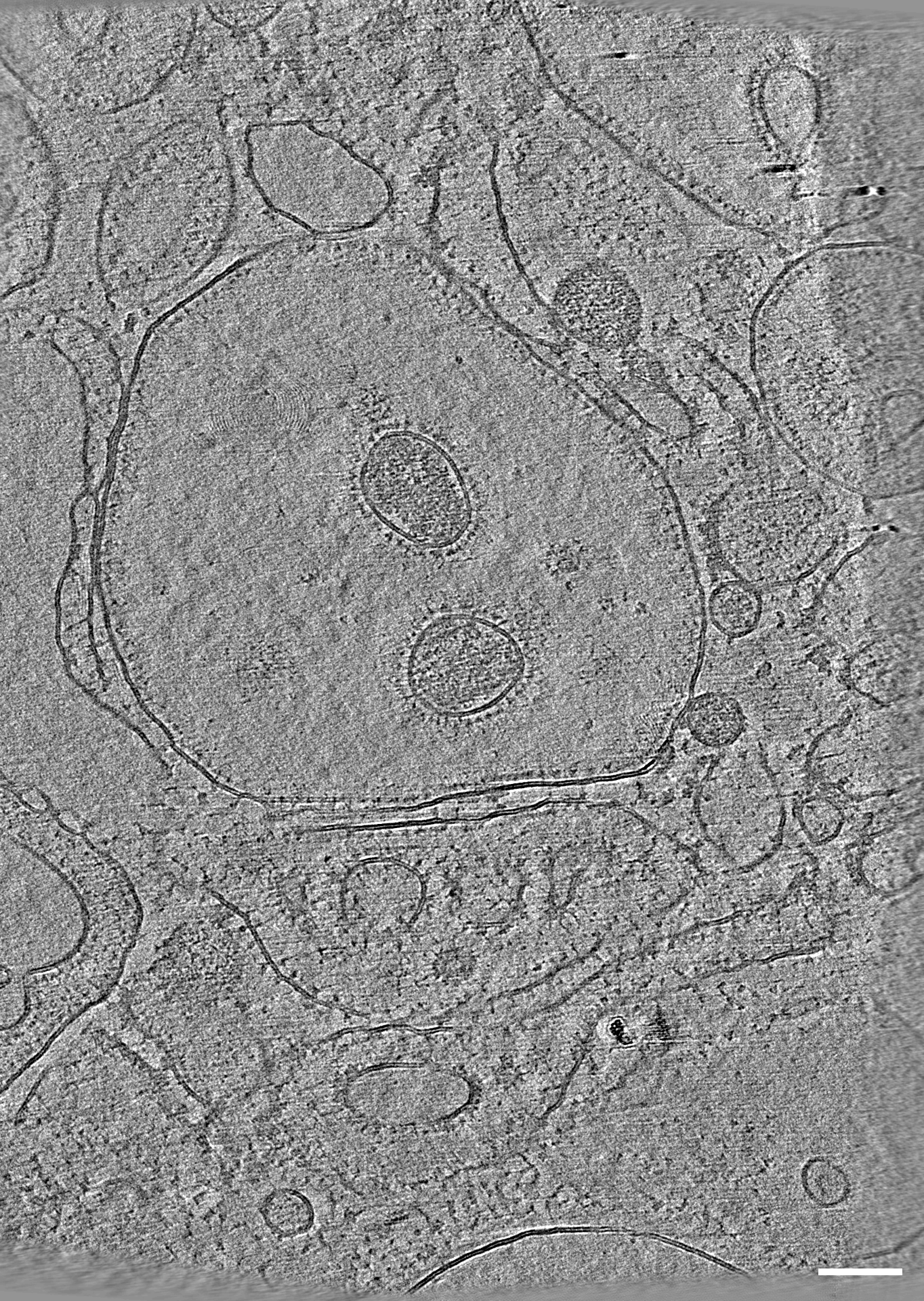

Supplement: Supplementary file 6 — Source Data for Expanded View and Appendix [file EMBJ-42-e113578-s006.zip › SupplementaryFigures_SourceData/FigureEV3/PanelB/AVG10_TS15o_bin3_bandpass1_100nm.png]

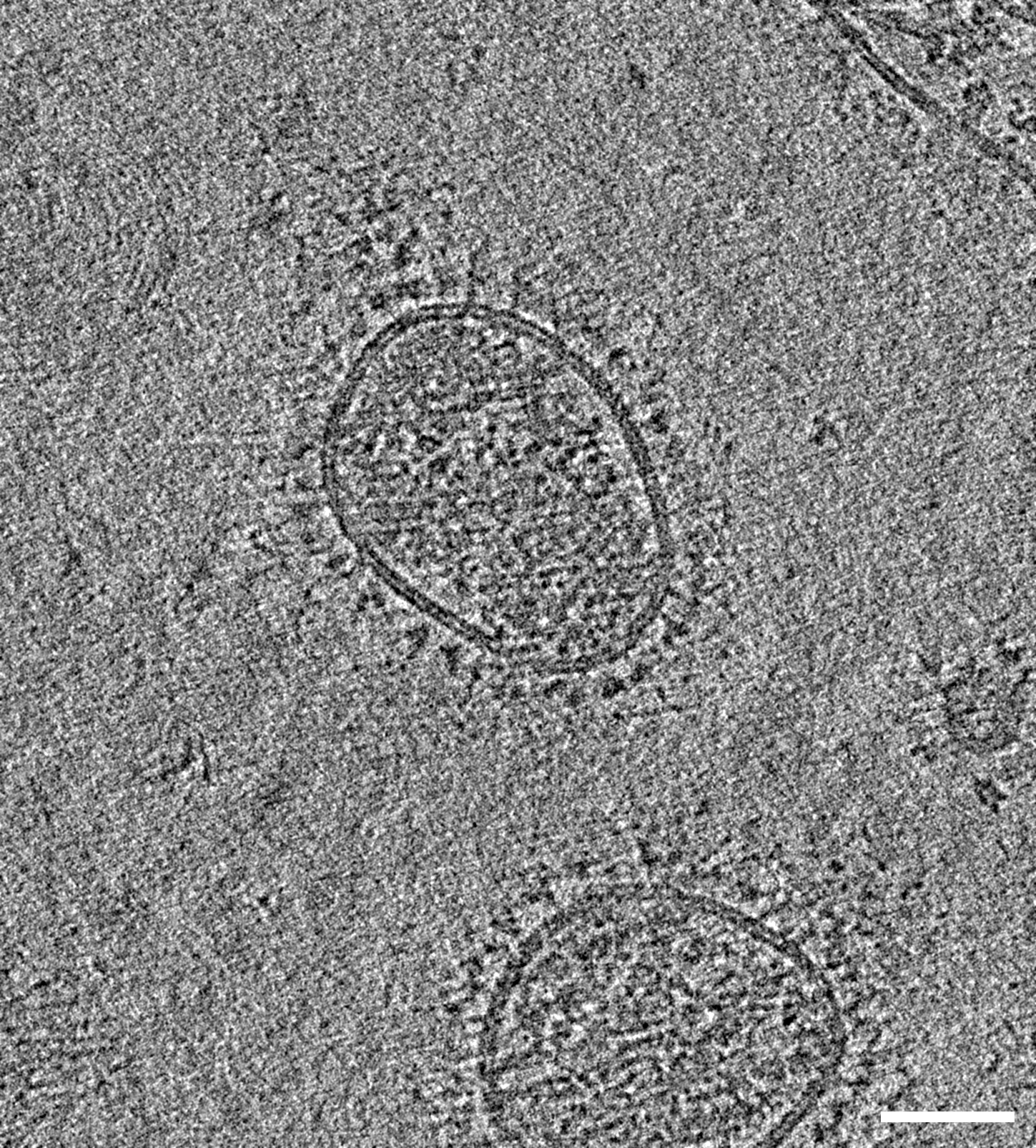

Supplement: Supplementary file 6 — Source Data for Expanded View and Appendix [file EMBJ-42-e113578-s006.zip › SupplementaryFigures_SourceData/FigureEV3/PanelC/AVG10_TS15o_bin1_bandpass2_50nm.png]

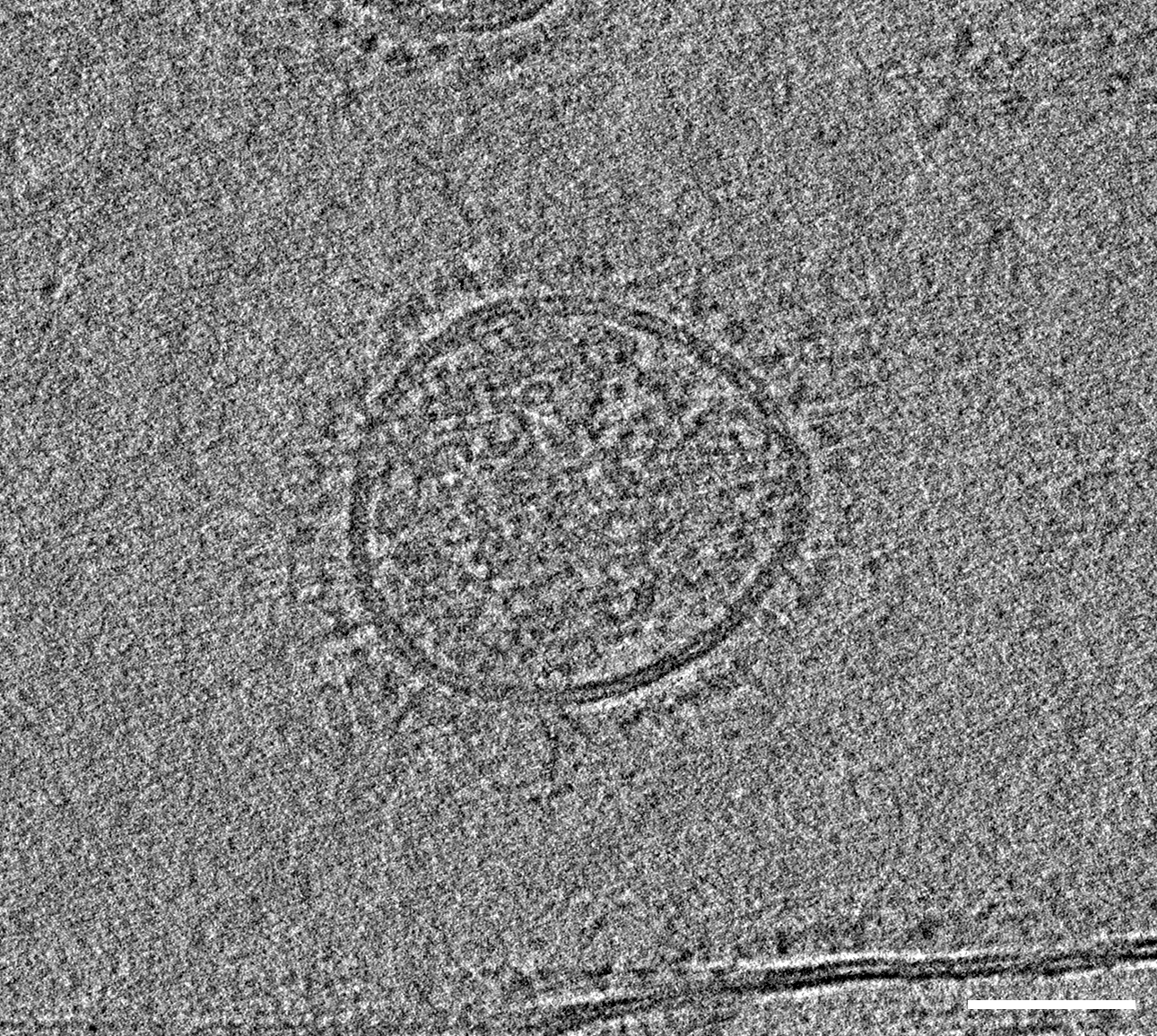

Supplement: Supplementary file 6 — Source Data for Expanded View and Appendix [file EMBJ-42-e113578-s006.zip › SupplementaryFigures_SourceData/FigureEV3/PanelD/AVG10_TS15o_bin1_bandpass2_50nm_4.png]

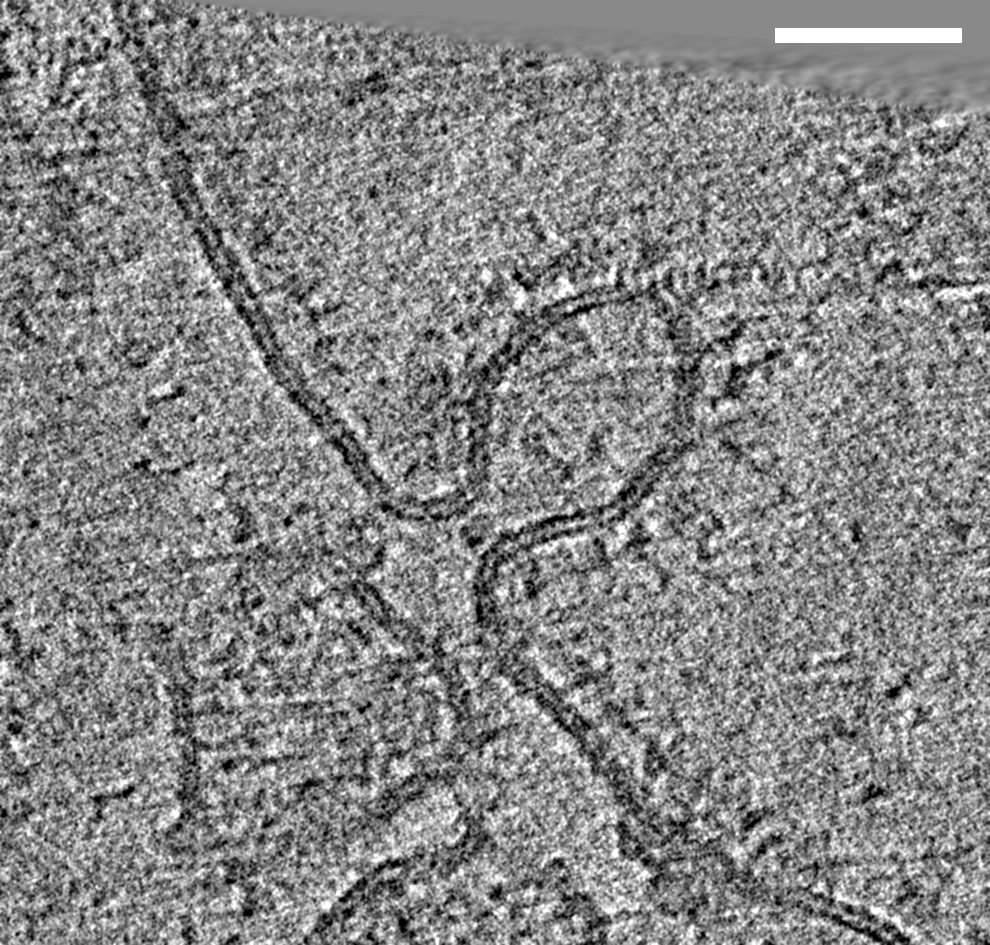

Supplement: Supplementary file 6 — Source Data for Expanded View and Appendix [file EMBJ-42-e113578-s006.zip › SupplementaryFigures_SourceData/FigureEV3/PanelE/AVG10_TS15o_bin1_bandpass2_50nm_2.png]

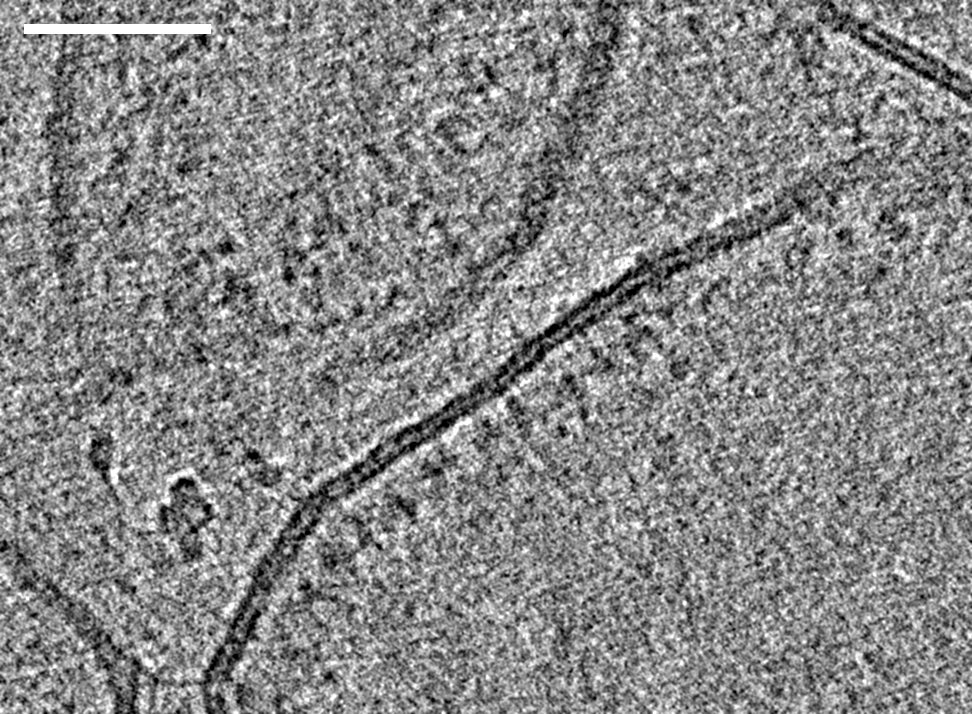

Supplement: Supplementary file 6 — Source Data for Expanded View and Appendix [file EMBJ-42-e113578-s006.zip › SupplementaryFigures_SourceData/FigureEV3/PanelF/AVG10_TS15o_bin1_bandpass2_50nm_3.png]

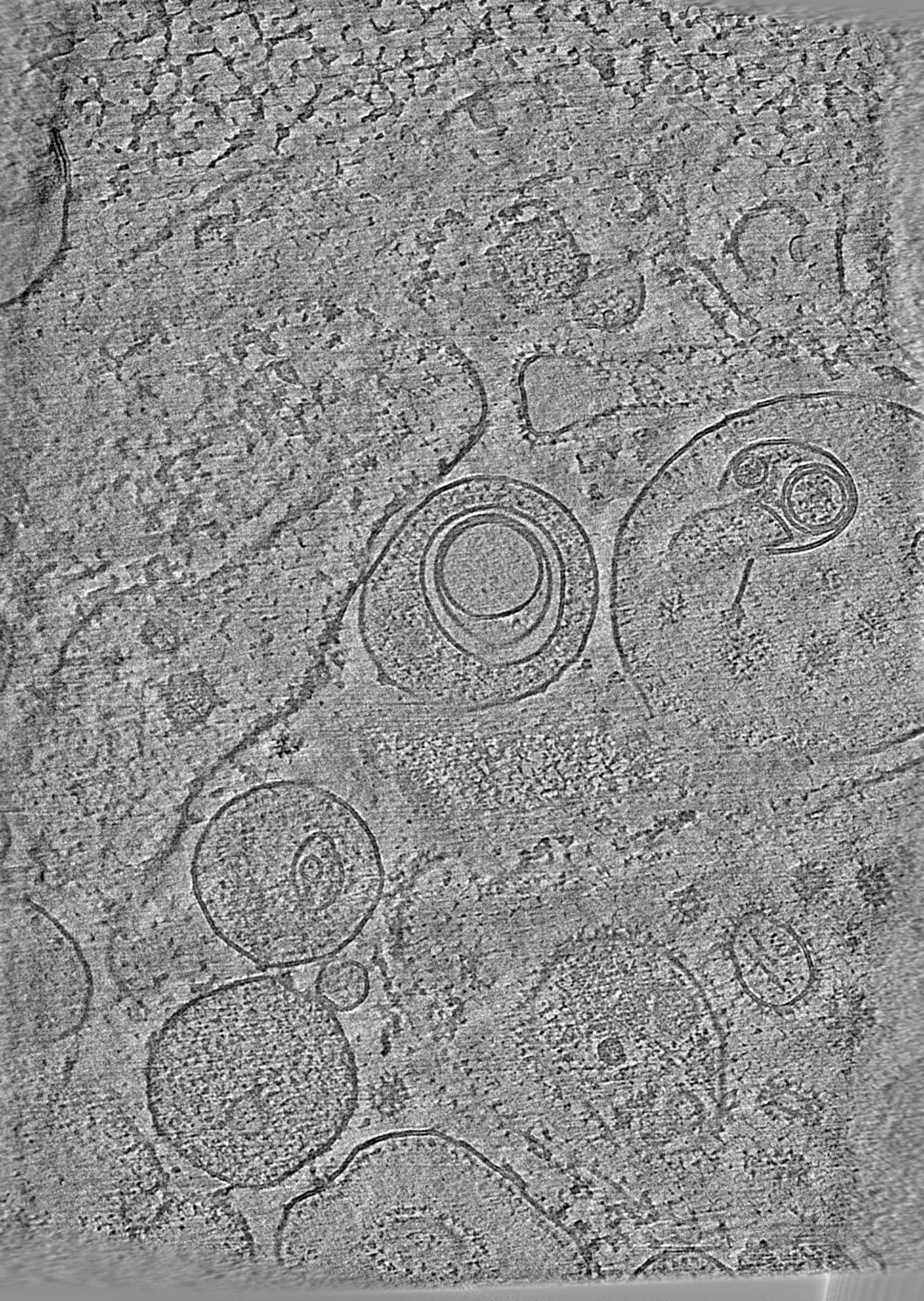

Supplement: Supplementary file 6 — Source Data for Expanded View and Appendix [file EMBJ-42-e113578-s006.zip › SupplementaryFigures_SourceData/FigureEV3/PanelG/AVG10_TS2o_bin3_bandpass1_100nm_2.png]

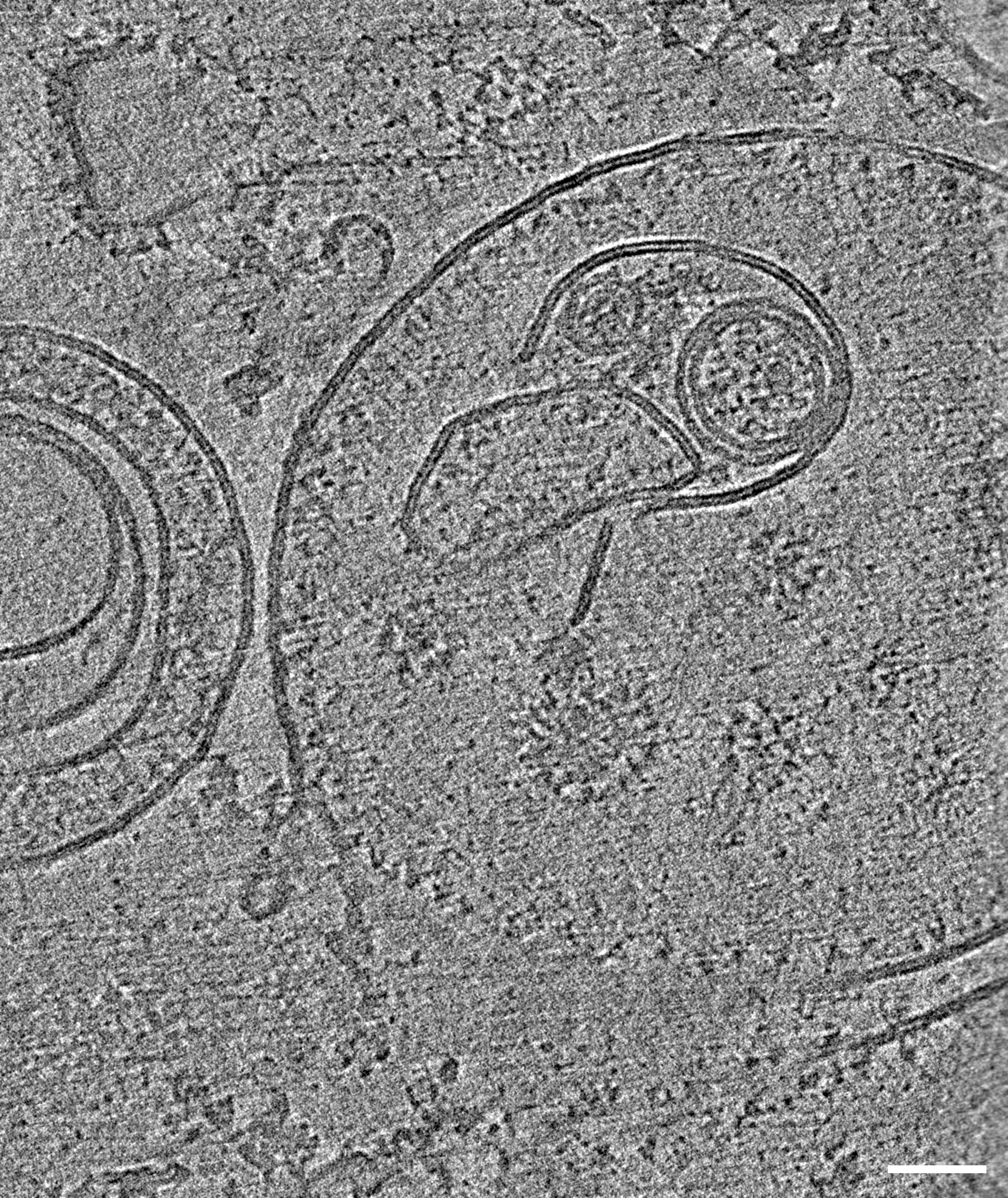

Supplement: Supplementary file 6 — Source Data for Expanded View and Appendix [file EMBJ-42-e113578-s006.zip › SupplementaryFigures_SourceData/FigureEV3/PanelH/AVG10_TS2o_bin1_bandpass2_50nm.png]

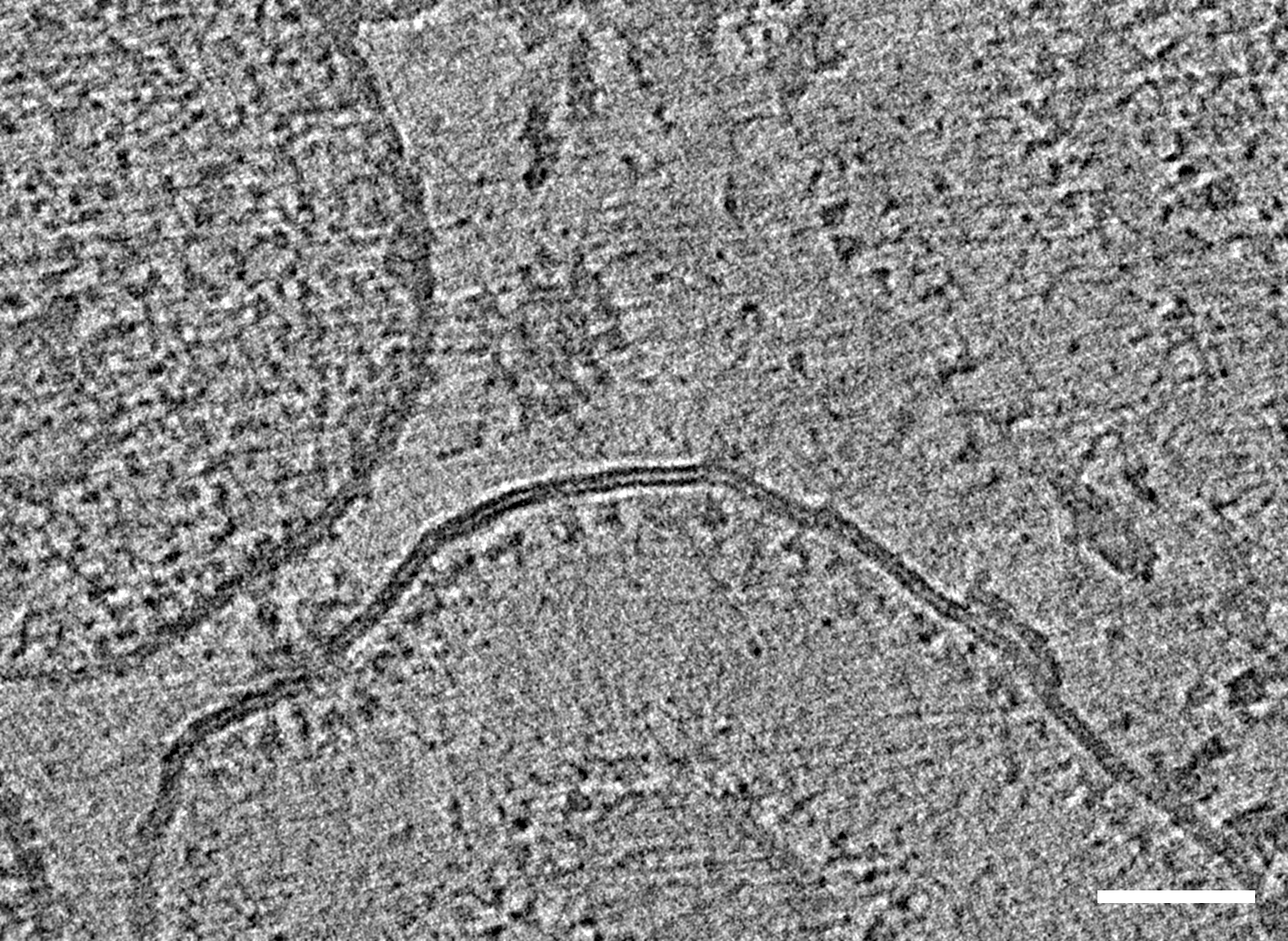

Supplement: Supplementary file 6 — Source Data for Expanded View and Appendix [file EMBJ-42-e113578-s006.zip › SupplementaryFigures_SourceData/FigureEV3/PanelI/AVG10_TS2o_bin1_bandpass2_50nm_2.png]

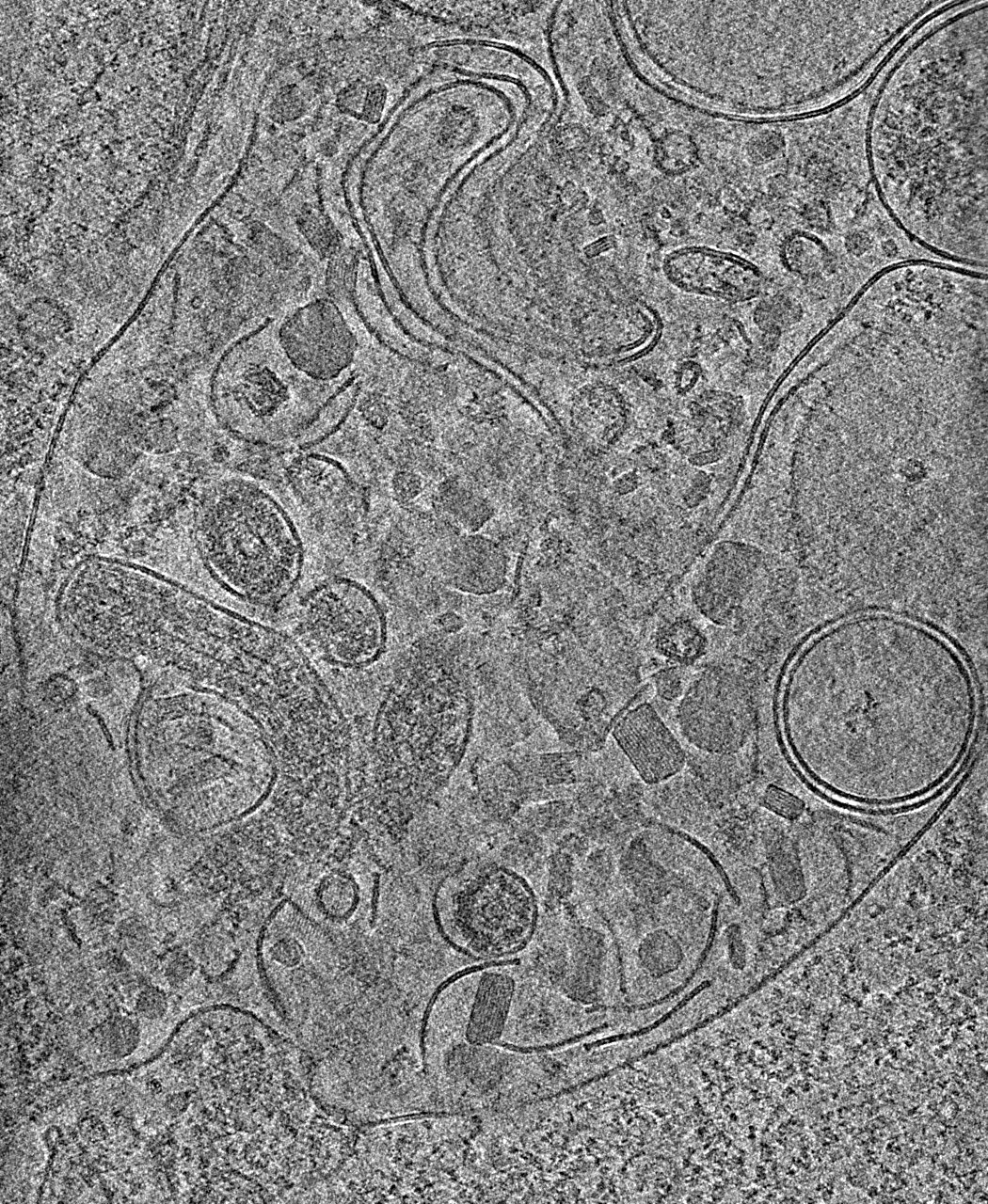

Supplement: Supplementary file 8 — Source Data for Figure 1 [file EMBJ-42-e113578-s009.zip › Figure1/PanelB/MAX10_TS_02_bin3_bandpass1_2.tif]

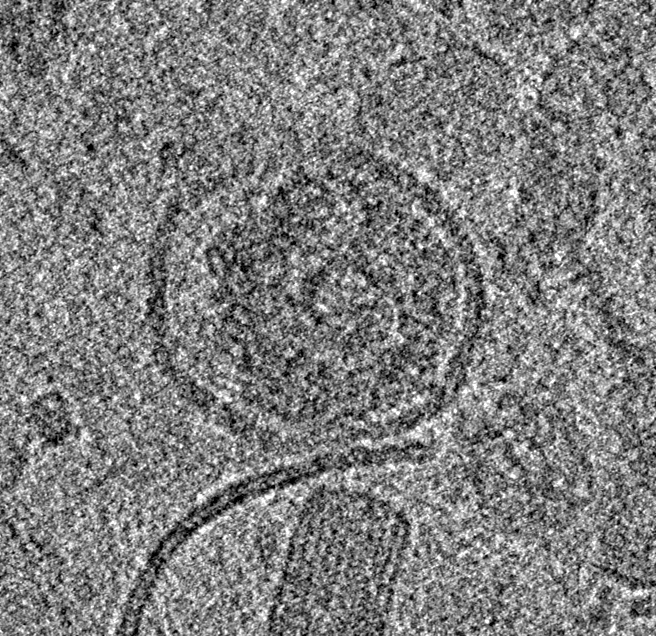

Supplement: Supplementary file 8 — Source Data for Figure 1 [file EMBJ-42-e113578-s009.zip › Figure1/PanelD/MAX10_TS_02_bin1__bandpass1.tif]

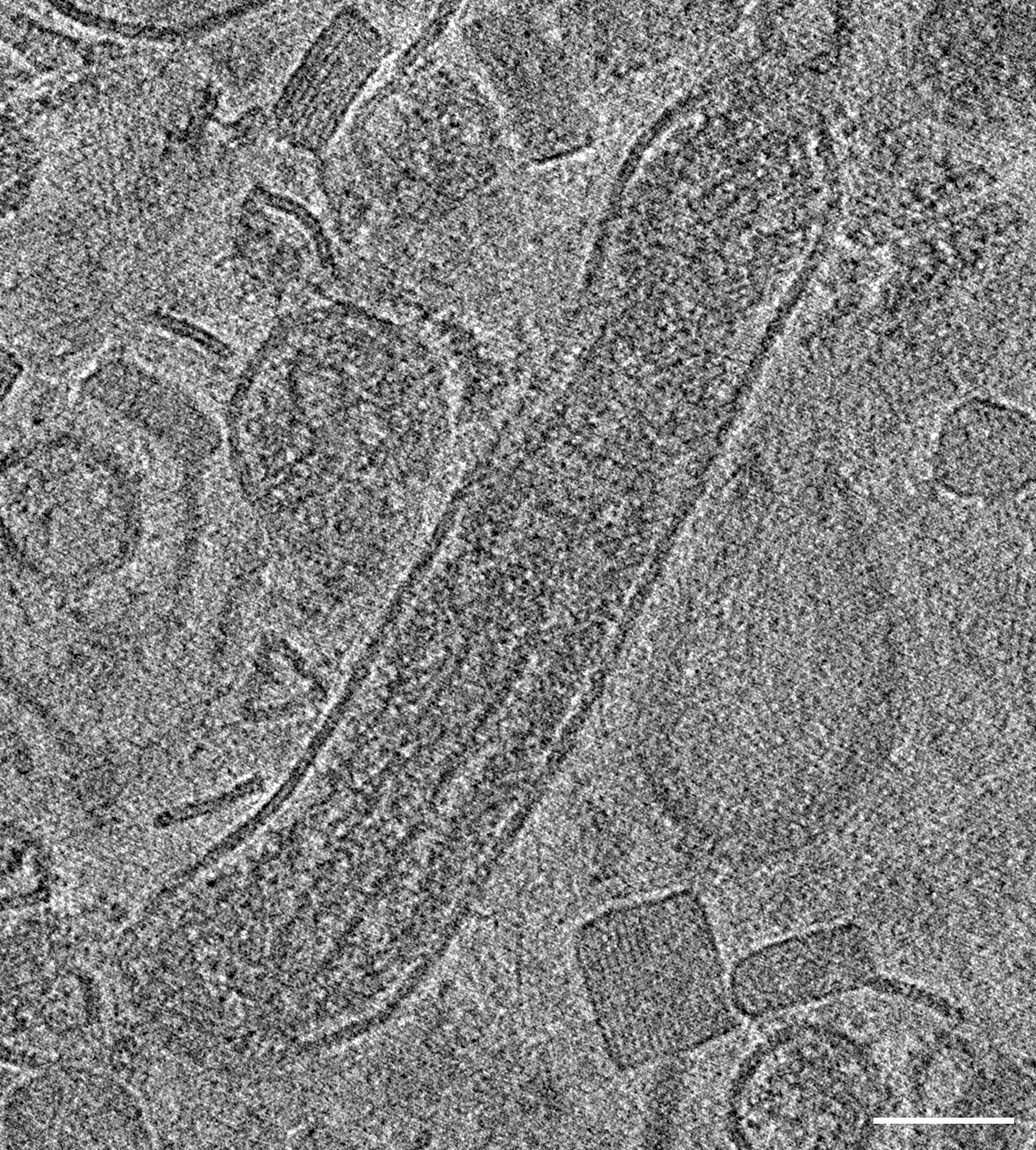

Supplement: Supplementary file 8 — Source Data for Figure 1 [file EMBJ-42-e113578-s009.zip › Figure1/PanelF/vp40-3_AVG20_bandpass1_50nm.png]

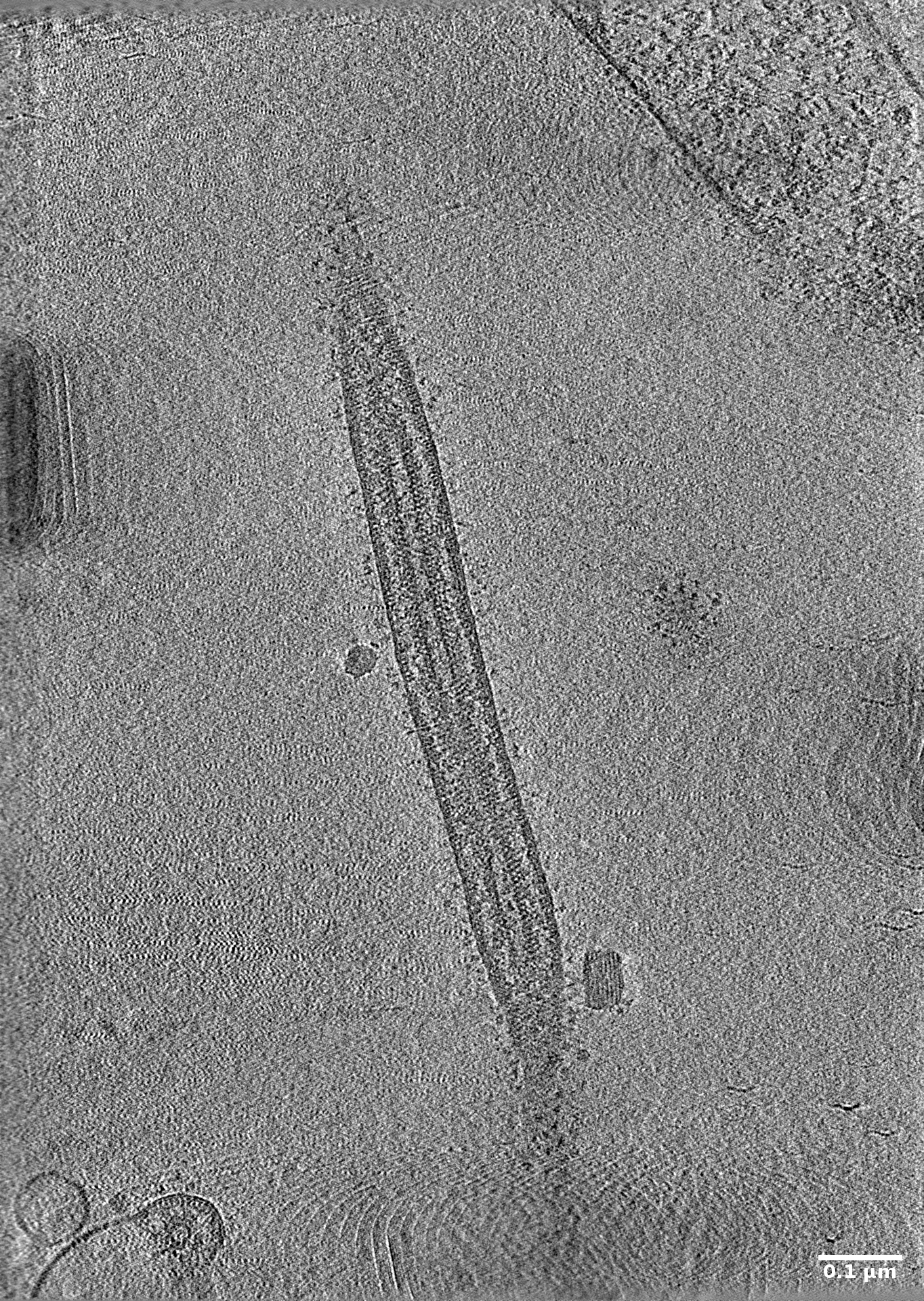

Supplement: Supplementary file 8 — Source Data for Figure 1 [file EMBJ-42-e113578-s009.zip › Figure1/PanelH/AVG10_TS03_bin3_bandpass1_100nm_2.png]

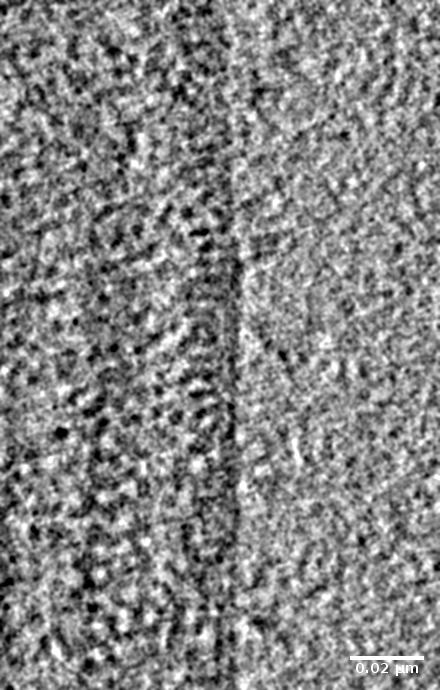

Supplement: Supplementary file 8 — Source Data for Figure 1 [file EMBJ-42-e113578-s009.zip › Figure1/PanelJ/AVG10_TS_01_bandpass3_20nm_2.png]

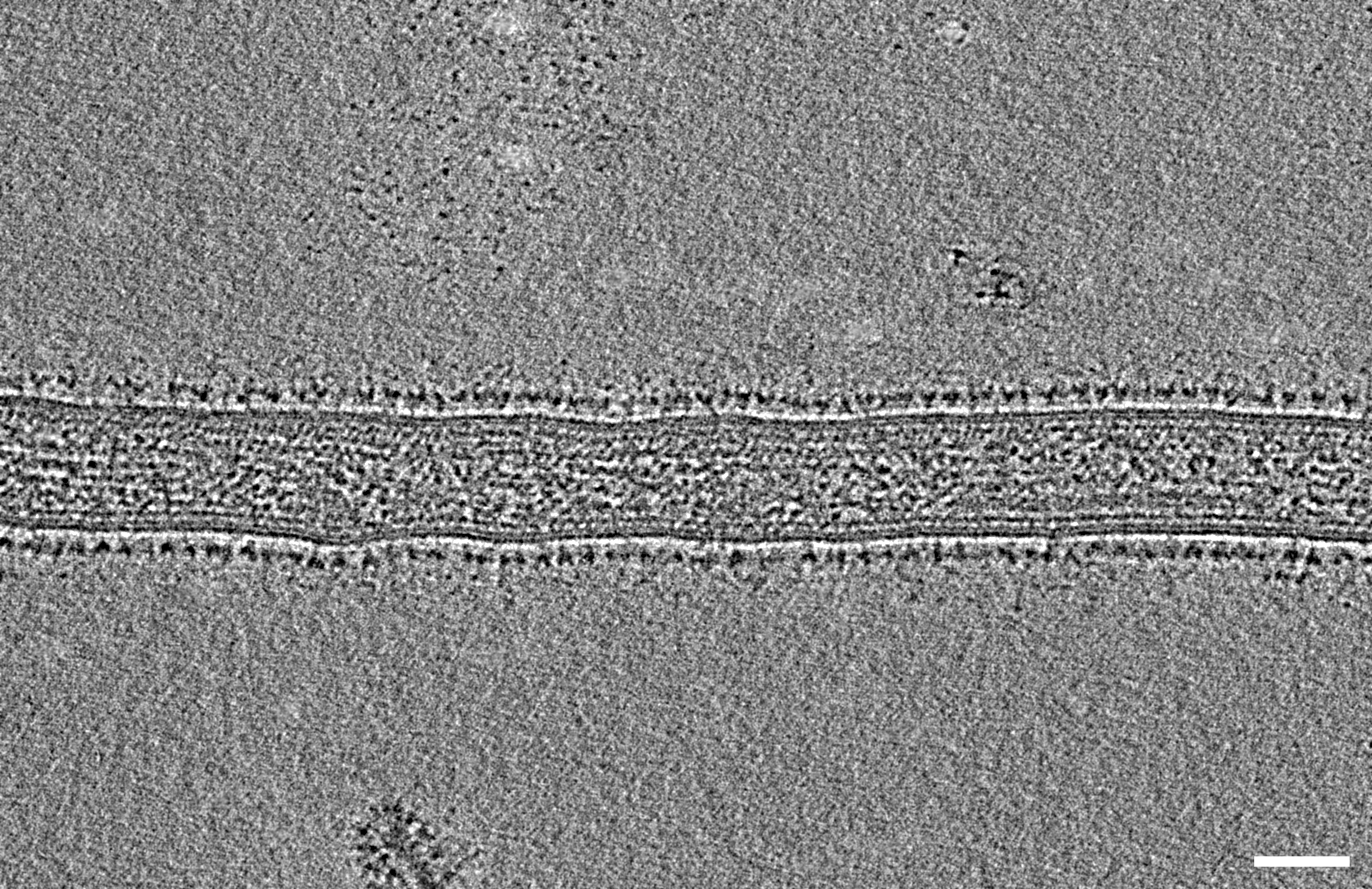

Supplement: Supplementary file 9 — Source Data for Figure 2 [file EMBJ-42-e113578-s008.zip › Figure2/PanelA/AVG10_M11_TS02_bin1_bandpass3_50m_cross.png]

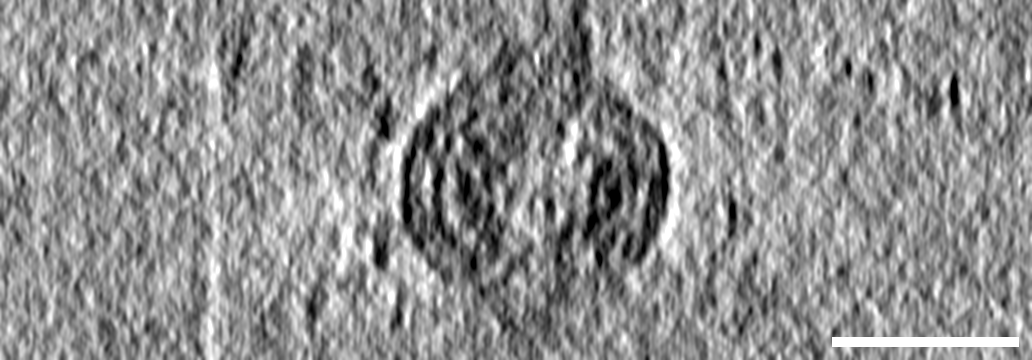

Supplement: Supplementary file 9 — Source Data for Figure 2 [file EMBJ-42-e113578-s008.zip › Figure2/PanelB/AVG20_M11_TS01_bin1_bandpass3_50nm.png]

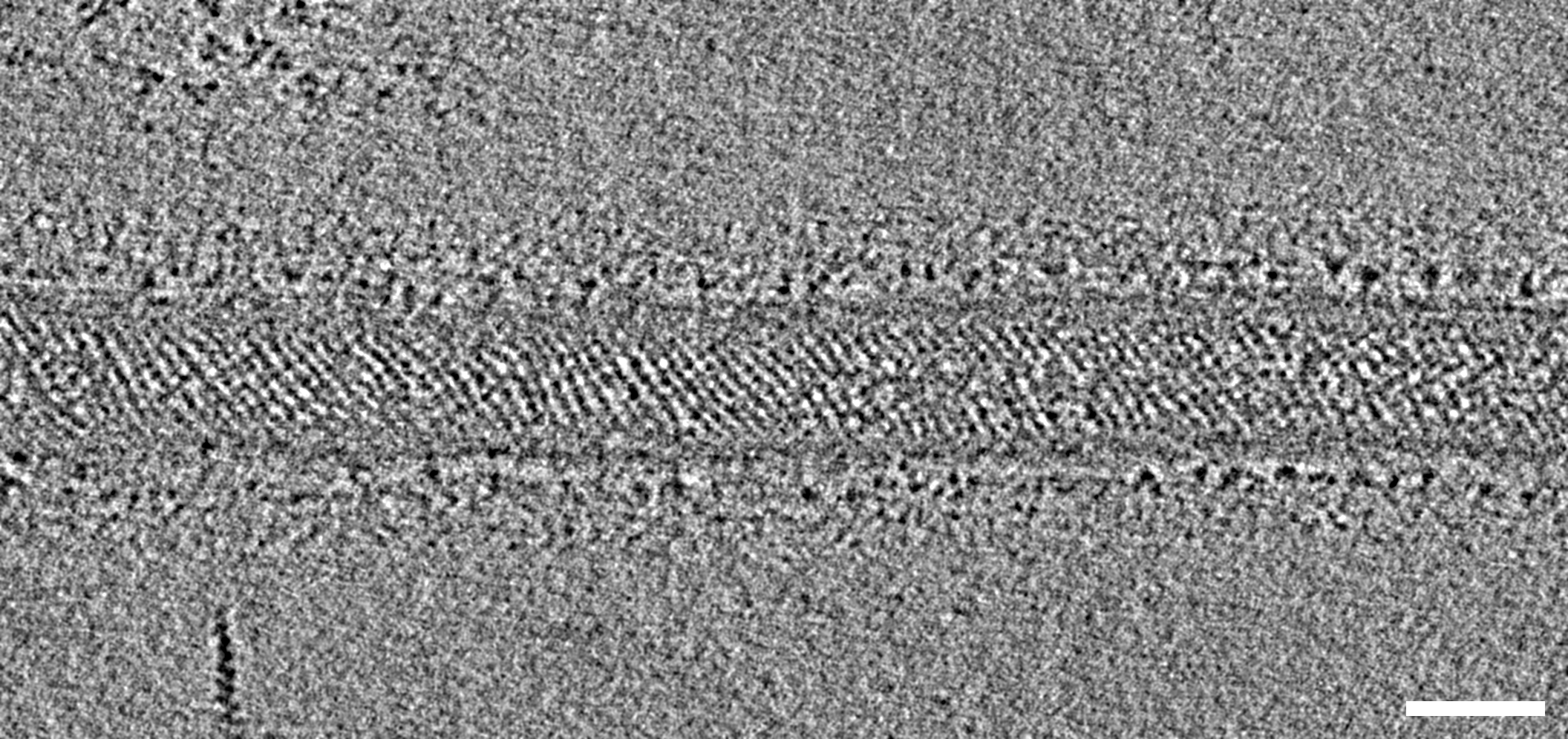

Supplement: Supplementary file 9 — Source Data for Figure 2 [file EMBJ-42-e113578-s008.zip › Figure2/PanelC/AVG10_M11_TS02_bin1_bandpass3_50m.png]

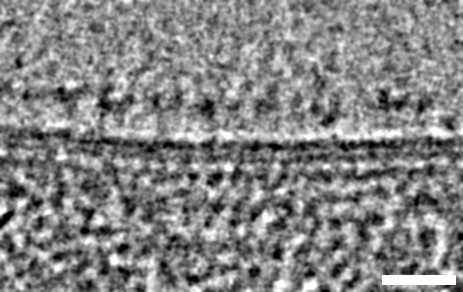

Supplement: Supplementary file 9 — Source Data for Figure 2 [file EMBJ-42-e113578-s008.zip › Figure2/PanelD/AVG10_M11-2_TS02_bin1_bandpass3_20nm.png]

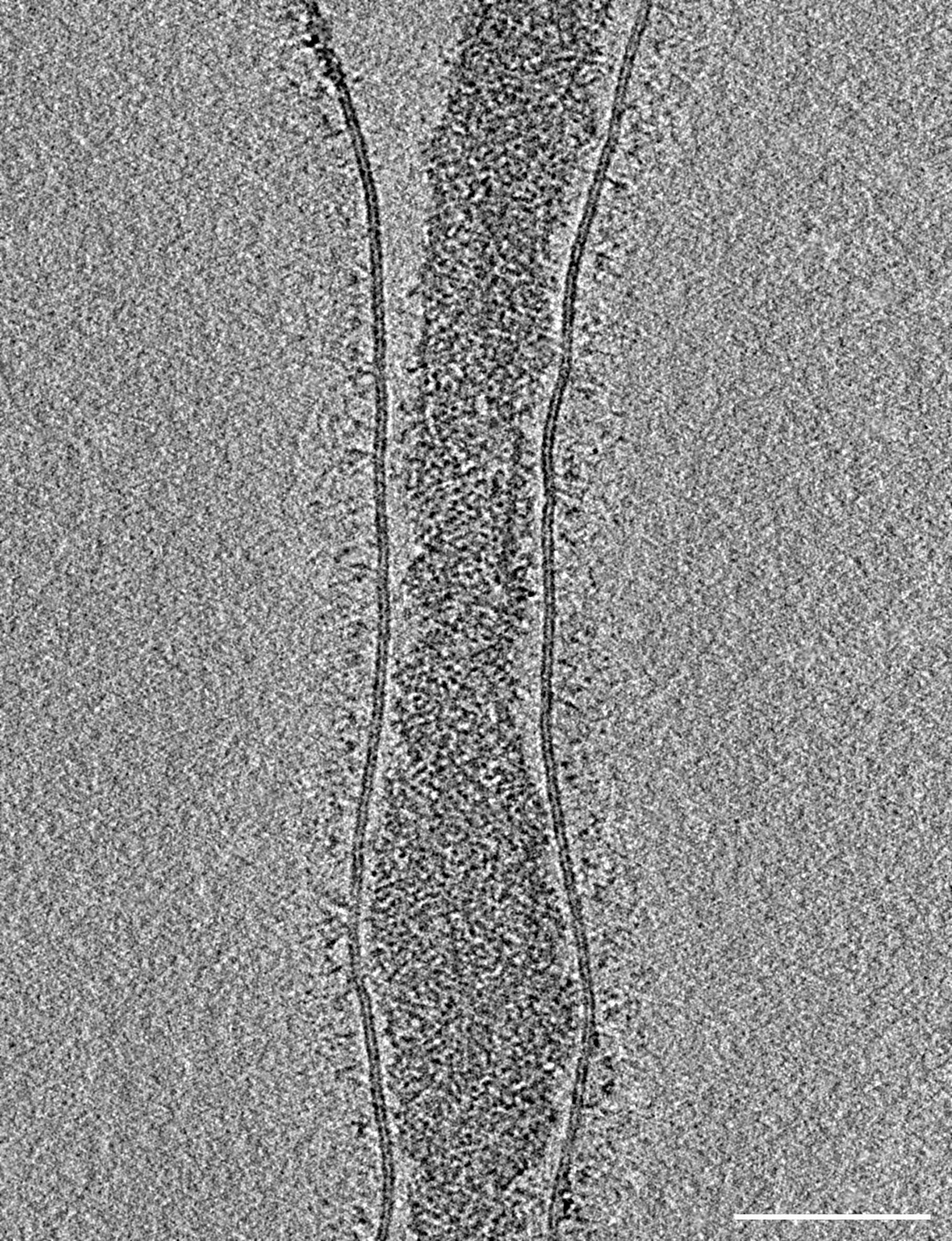

Supplement: Supplementary file 9 — Source Data for Figure 2 [file EMBJ-42-e113578-s008.zip › Figure2/PanelG/AVG10_M4_TS02_bin1_bandpass3_100nm.png]

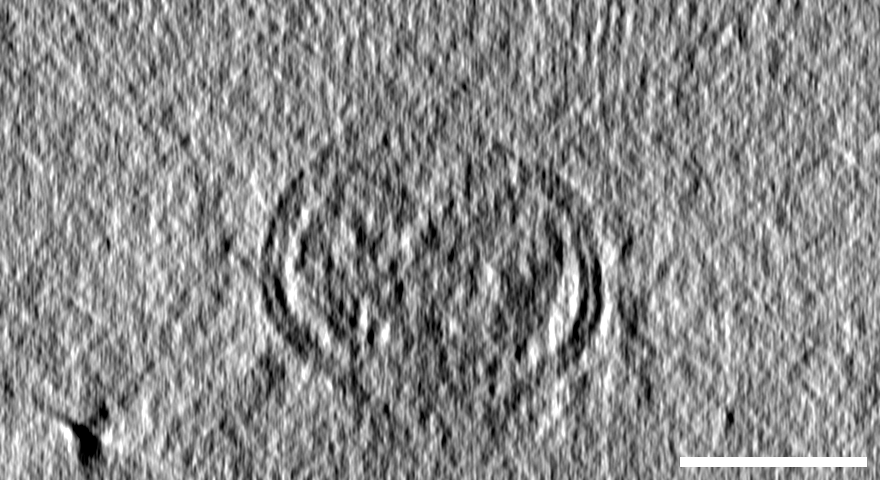

Supplement: Supplementary file 9 — Source Data for Figure 2 [file EMBJ-42-e113578-s008.zip › Figure2/PanelH/AVG20_M4_TS02_bin1_bandpass1_50nm.png]

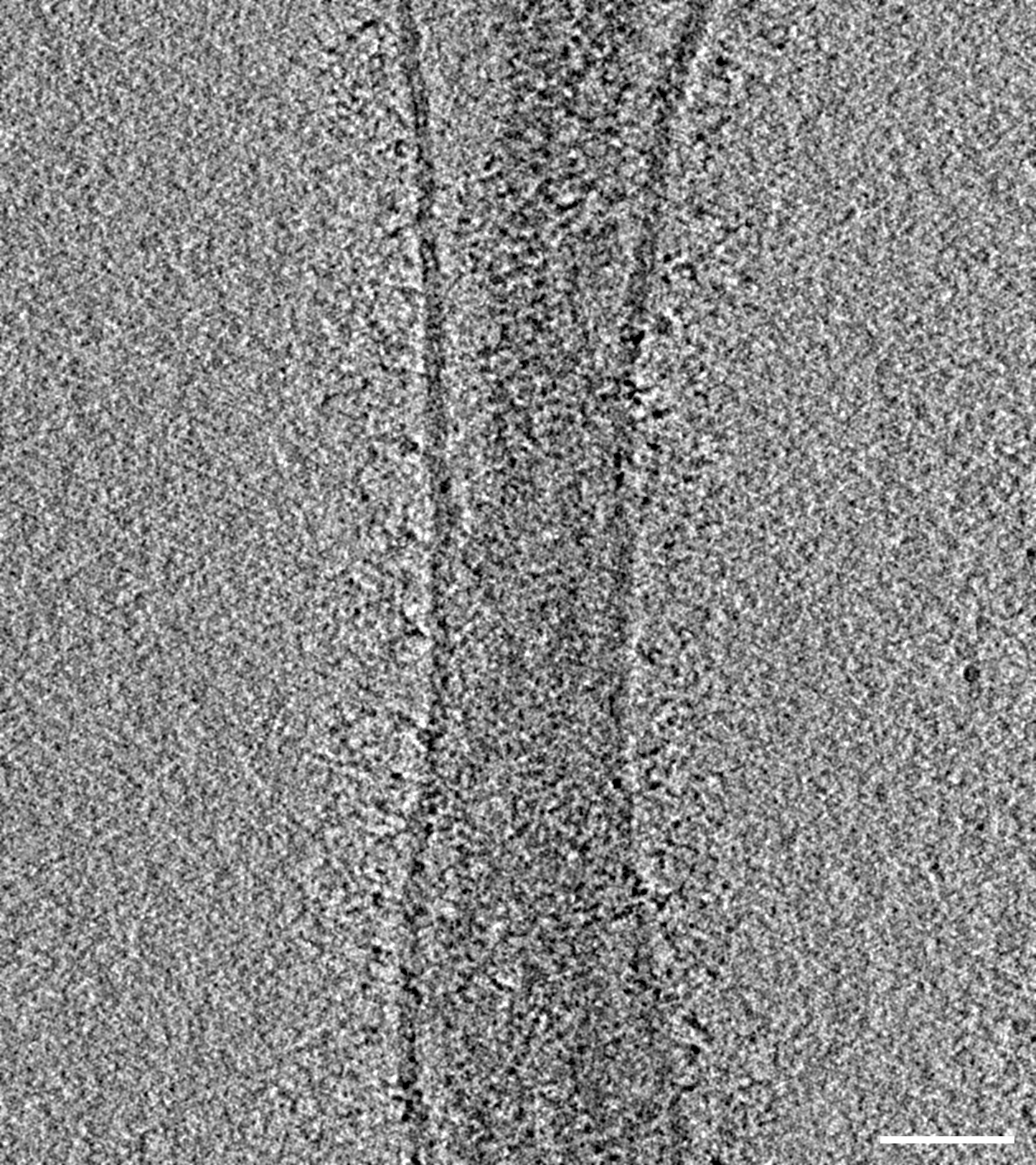

Supplement: Supplementary file 9 — Source Data for Figure 2 [file EMBJ-42-e113578-s008.zip › Figure2/PanelI/AVG10_M4_TS02_bin1_bandpass3_top_50nm.png]

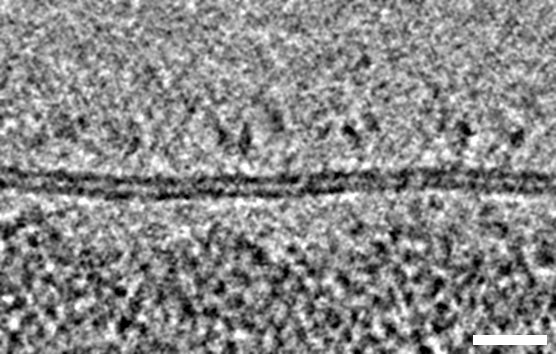

Supplement: Supplementary file 9 — Source Data for Figure 2 [file EMBJ-42-e113578-s008.zip › Figure2/PanelJ/AVG10_M4_TS02_bin1_bandpass3_zoom_20nm.png]

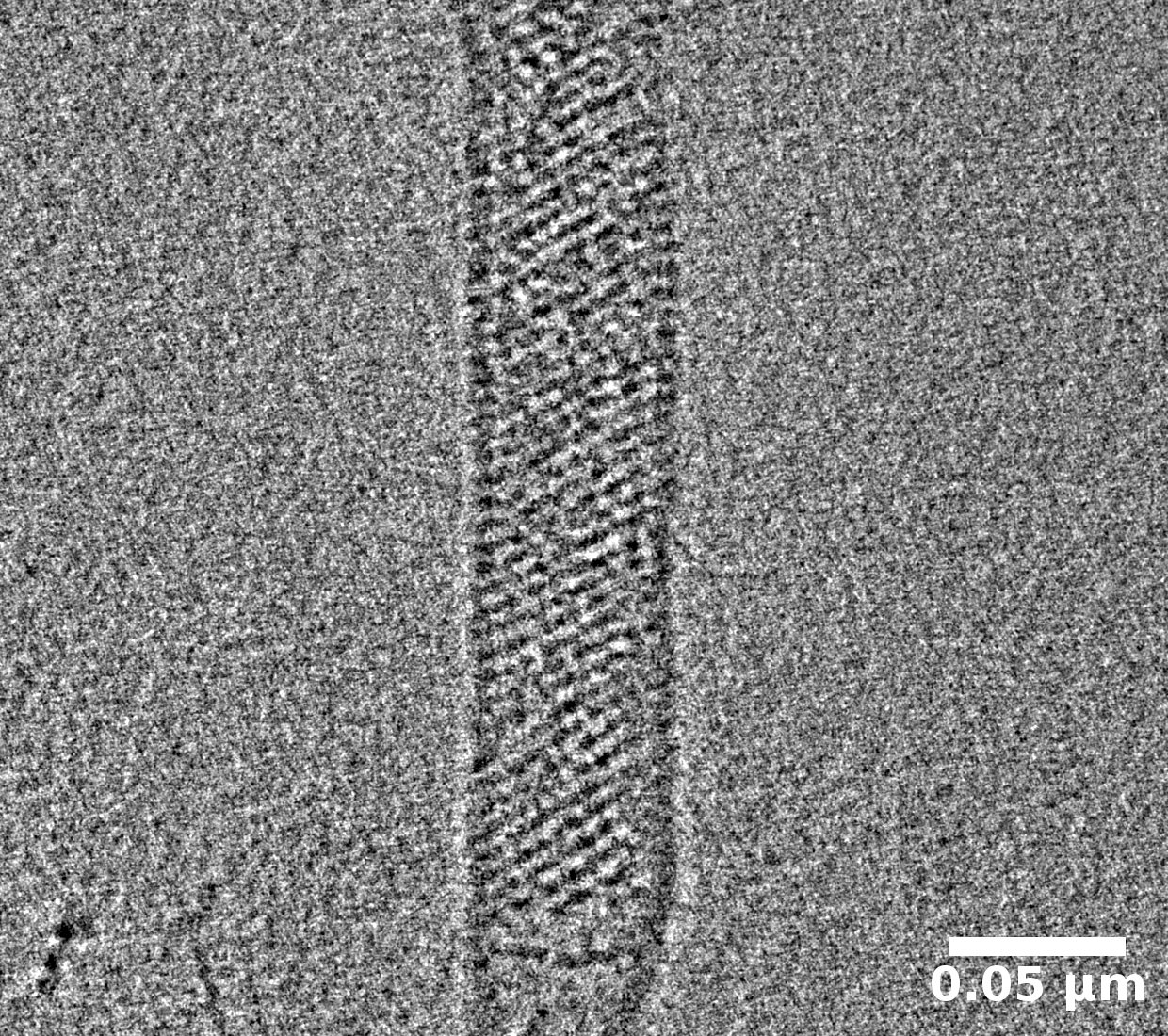

Supplement: Supplementary file 9 — Source Data for Figure 2 [file EMBJ-42-e113578-s008.zip › Figure2/PanelK/MAX10_TS_04o_bin1_bandpass1_50nm_top.png]

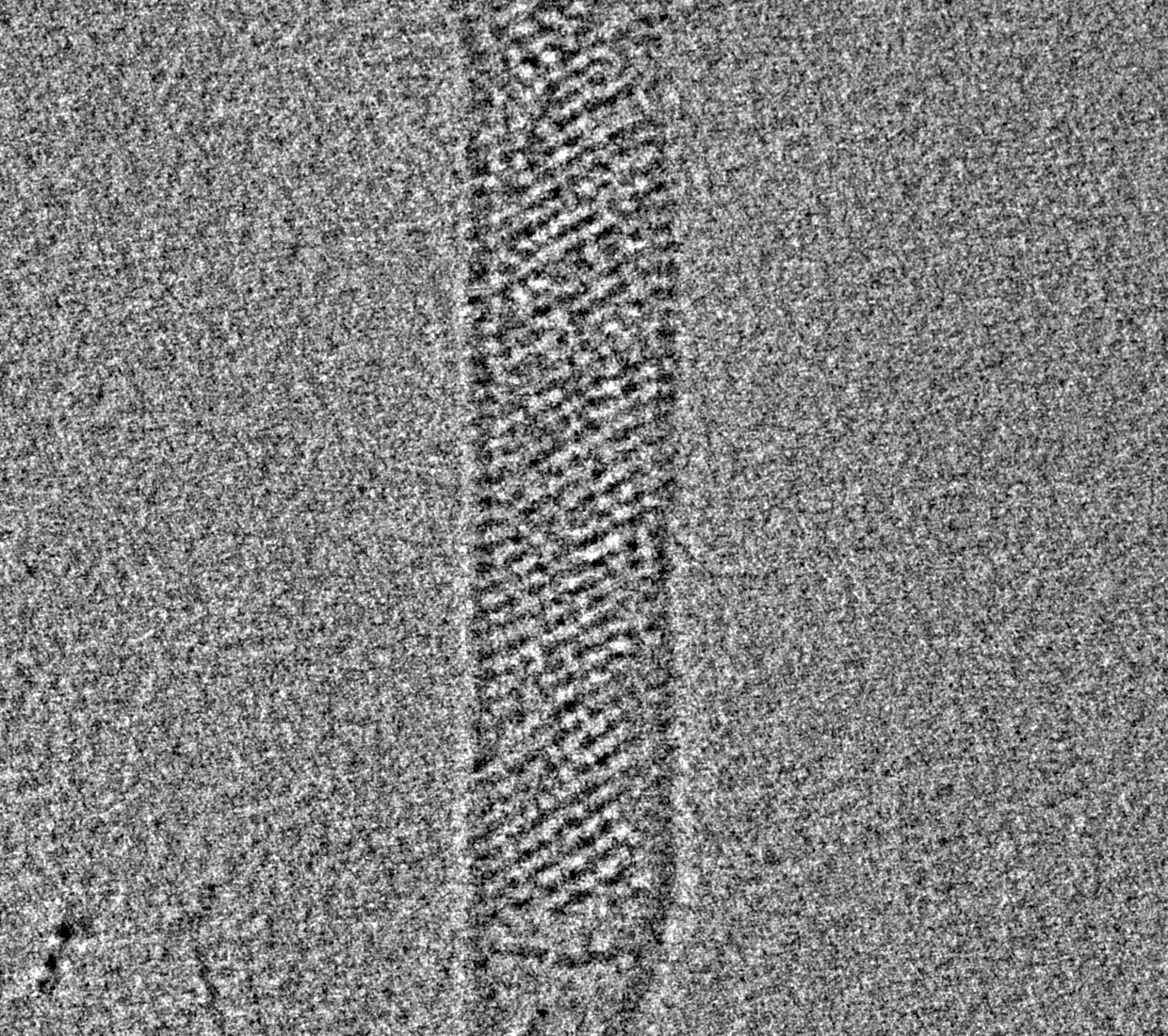

Supplement: Supplementary file 9 — Source Data for Figure 2 [file EMBJ-42-e113578-s008.zip › Figure2/PanelK/MAX10_TS_04o_bin1_bandpass1_50nm_top.tif]

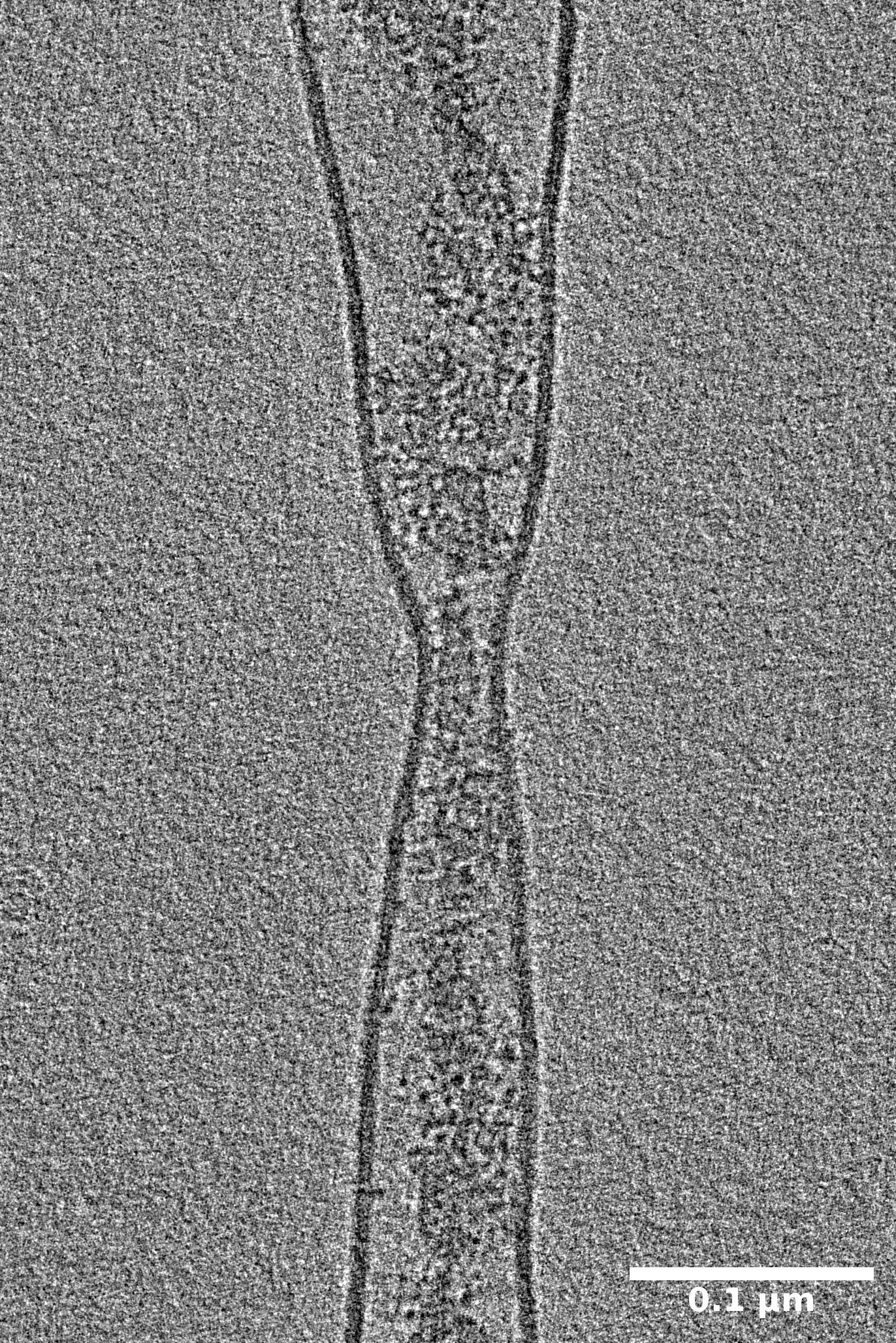

Supplement: Supplementary file 9 — Source Data for Figure 2 [file EMBJ-42-e113578-s008.zip › Figure2/PanelK/MAX10_TS_06_bin1_bandpass1_100nm.png]

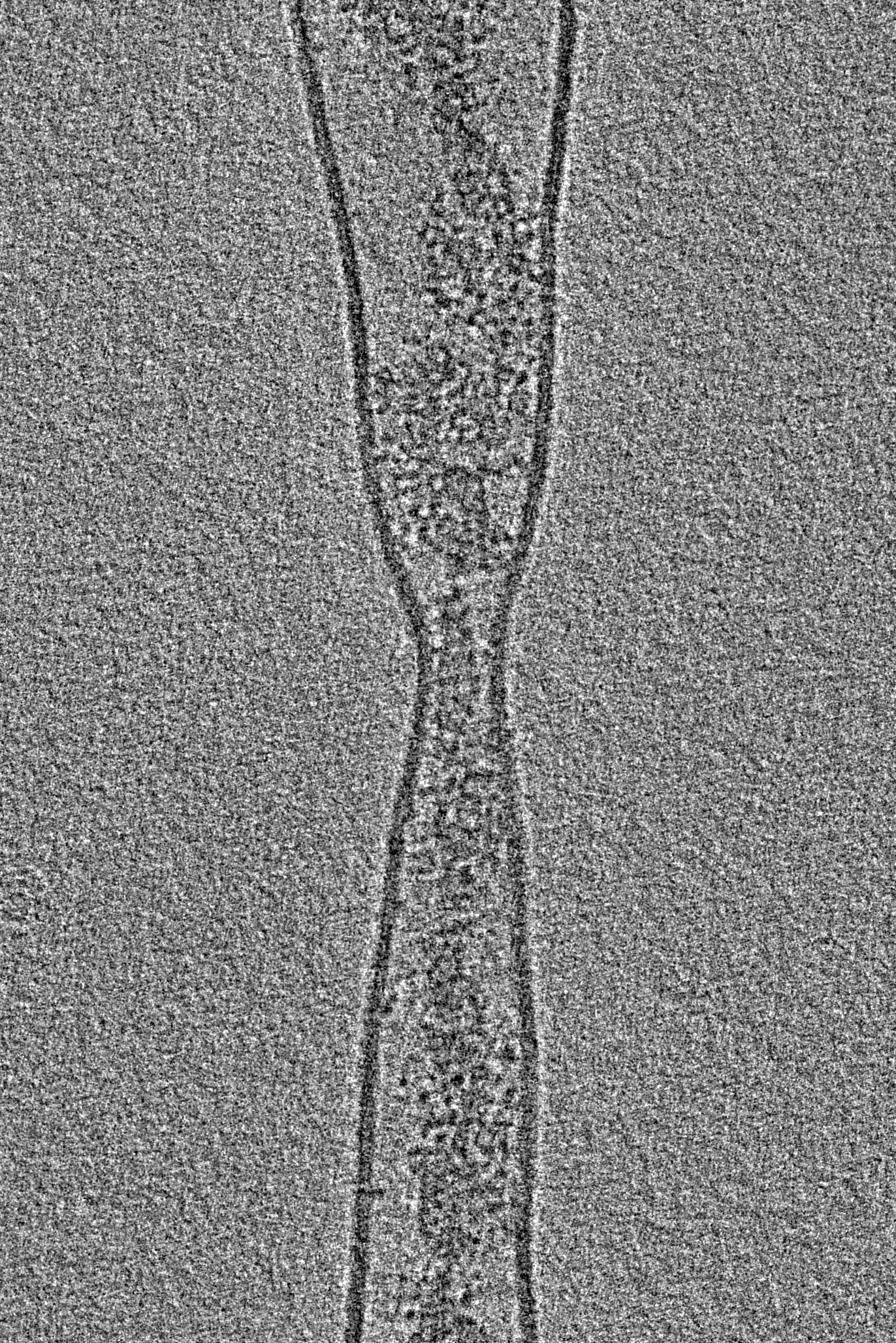

Supplement: Supplementary file 9 — Source Data for Figure 2 [file EMBJ-42-e113578-s008.zip › Figure2/PanelK/MAX10_TS_06_bin1_bandpass1_100nm.tif]

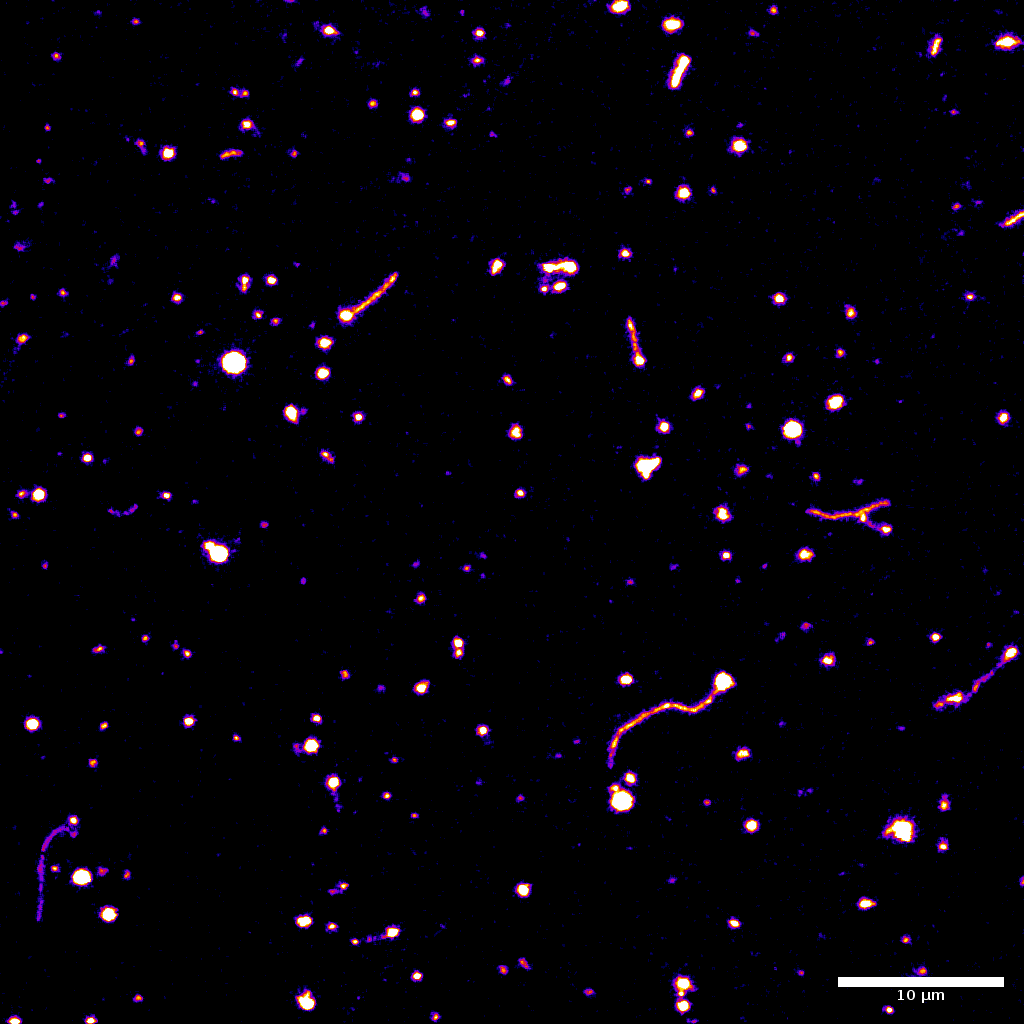

Supplement: Supplementary file 11 — Source Data for Figure 4 [file EMBJ-42-e113578-s010.zip › Figure4/PanelB/20190516_Pos002_10μm.png]

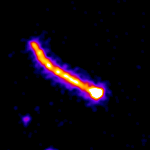

Supplement: Supplementary file 11 — Source Data for Figure 4 [file EMBJ-42-e113578-s010.zip › Figure4/PanelC/pH5/0min.png]

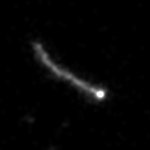

Supplement: Supplementary file 11 — Source Data for Figure 4 [file EMBJ-42-e113578-s010.zip › Figure4/PanelC/pH5/0min.tif]

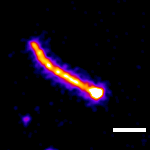

Supplement: Supplementary file 11 — Source Data for Figure 4 [file EMBJ-42-e113578-s010.zip › Figure4/PanelC/pH5/0min_2μm.png]

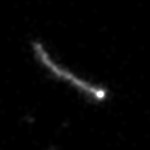

Supplement: Supplementary file 11 — Source Data for Figure 4 [file EMBJ-42-e113578-s010.zip › Figure4/PanelC/pH5/0min_2μm.tif]

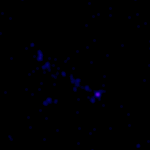

Supplement: Supplementary file 11 — Source Data for Figure 4 [file EMBJ-42-e113578-s010.zip › Figure4/PanelC/pH5/10min.png]

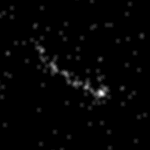

Supplement: Supplementary file 11 — Source Data for Figure 4 [file EMBJ-42-e113578-s010.zip › Figure4/PanelC/pH5/10min.tif]

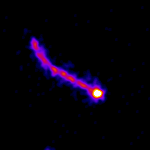

Supplement: Supplementary file 11 — Source Data for Figure 4 [file EMBJ-42-e113578-s010.zip › Figure4/PanelC/pH5/1min.png]

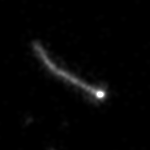

Supplement: Supplementary file 11 — Source Data for Figure 4 [file EMBJ-42-e113578-s010.zip › Figure4/PanelC/pH5/1min.tif]

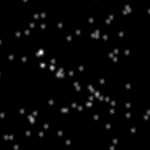

Supplement: Supplementary file 11 — Source Data for Figure 4 [file EMBJ-42-e113578-s010.zip › Figure4/PanelC/pH5/30min.tif]

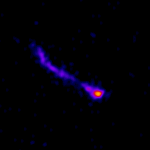

Supplement: Supplementary file 11 — Source Data for Figure 4 [file EMBJ-42-e113578-s010.zip › Figure4/PanelC/pH5/3min.png]

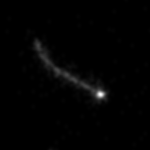

Supplement: Supplementary file 11 — Source Data for Figure 4 [file EMBJ-42-e113578-s010.zip › Figure4/PanelC/pH5/3min.tif]

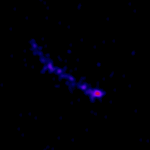

Supplement: Supplementary file 11 — Source Data for Figure 4 [file EMBJ-42-e113578-s010.zip › Figure4/PanelC/pH5/5min.png]

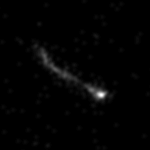

Supplement: Supplementary file 11 — Source Data for Figure 4 [file EMBJ-42-e113578-s010.zip › Figure4/PanelC/pH5/5min.tif]

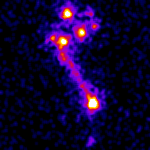

Supplement: Supplementary file 11 — Source Data for Figure 4 [file EMBJ-42-e113578-s010.zip › Figure4/PanelC/pH7p4/0min_ph7.png]
