# Supplementary material for: The Ebola virus VP40 matrix layer undergoes endosomal disassembly essential for membrane fusion
Source: EMBO J. 2023 Apr 21;42(11):e113578. doi: 10.15252/embj.2023113578 (PMC10233383; doi:10.15252/embj.2023113578)
Supplement: Supplementary file 13 [file EMBJ-42-e113578-s005.pdf]

THE EMBO JOURNAL

The Ebola virus VP40 matrix layer undergoes endosomal disassembly essential for membrane fusion

Sophie Winter, Gonen Golani, Fabio Lolicato, Melina Vallbracht, Keerthihan Thiyagarajah, Samy Ahmed, Christian Luechtenborg, Oliver Fackler, Britta Bruegger, Thomas Hoenen, Walter Nickel, Ulrich Schwarz, and Petr Chlanda
DOI: 10.15252/embj.2023113578

Corresponding author(s): Petr Chlanda (petr.chlanda@bioquant.uni-heidelberg.de)

Review Timeline:

Transfer from Review Commons:	21st Jan 23
Editorial Decision:	14th Feb 23
Revision Received:	9th Mar 23
Accepted:	22nd Mar 23

Editor: William Teale

Transaction Report: This manuscript was transferred to The EMBO Journal following peer review at Review Commons.

Review COMMONS

(Note: With the exception of the correction of typographical or spelling errors that could be a source of ambiguity, letters and reports are not edited. Depending on transfer agreements, referee reports obtained elsewhere may or may not be included in this compilation. Referee reports are anonymous unless the Referee chooses to sign their reports.)

Review #1

1. Evidence, reproducibility and clarity:

Evidence, reproducibility and clarity (Required)

Winter et al. present a study of Ebola virus fusion in the acidic environment of the late endosome. Based on cryo-ET of Ebola virions undergoing entry into cells, they note that the VP40 matrix is disassembled and dissociated from the viral membrane in virions seen in the endosome. Subsequent in vitro and computational analyses suggest that protons diffuse across the viral membrane and neutralize anionic lipids on the inner leaflet. They argue that this loss of negative charge reduces the affinity of VP40 for the viral membrane. They further suggest that VP40 dissociation from the viral membrane precedes GP-mediated membrane fusion and contributes to reduction in the energy barrier for membrane stalk formation. Whereas most studies have focused on the importance of acidic pH in triggering GP conformational changes during fusion, the present work contributes new appreciation for VP40-membrane interactions.

- In the cryo-ET experiments aimed at visualizing Ebola entry, do the authors see evidence of viral membrane fusion? There is no mention of this in the text. Knowing that the virions that show disassembly of the VP40 matrix are in fact the virions that productively enter cells would support the conclusions of the study. As is stands, one is forced to wonder whether the virions that show VP40 disassembly prior to fusion ultimately fuse.

- In the cryo-ET experiments that evaluate VP40 disassembly in vitro, why do the authors leave out NP from their VLP preparations? There is some evidence in the literature (Li et al., JVI 2016) that NP is necessary to form particles with native morphology. If the authors feel that NP is not necessary for their experiment, perhaps this could be noted.

- The authors argue that acidic pH neutralizes the charge of PS phospholipids, thereby removing the electrostatic interactions of basic residues in VP40 and PS. They also note in the Methods section that 7 amino acids in VP40 are predicted by PROPKA to be protonated at pH 4.5. If the authors feel that protonation of these 7 amino acids is not involved in the loss of affinity for PS, this could be stated explicitly and justified. Could the protonation of these 7 amino acids contribute to disassembly of the VP40 lattice, rather than dissociation from the membrane?

- *Minor:* Figure S5C is difficult to interpret. The red frame on the bars that indicates data acquired at low pH is nearly invisible. Better might be to indicate explicitly (ie, with words) the pH at which data were acquired.

2. Significance:

Significance (Required)

The significance of the study stems from the idea that the VP40 lattice and its interaction with the viral membrane plays a direct role in facilitating viral fusion. To my knowledge, this has not been previously addressed. The significance would be considerably increased if the authors were able to demonstrate by cryo-ET that the virions with disassembled VP40 were in fact the virions that productively fused. Nonetheless, this work should be of broad interest to researchers studying viral fusion as it may represent a phenomenon relevant to numerous viruses that enter cells via the endocytic route.

3. How much time do you estimate the authors will need to complete the suggested revisions:

Estimated time to Complete Revisions (Required)

(Decision Recommendation)

Between 1 and 3 months

4. *Review Commons* values the work of reviewers and encourages them to get credit for their work. Select 'Yes' below to register your reviewing activity at Web of Science Reviewer Recognition Service (formerly Publons); note that the content of your review will not be visible on Web of Science.

Web of Science Reviewer Recognition

Yes

Review #2

1. Evidence, reproducibility and clarity:

Evidence, reproducibility and clarity (Required)

The manuscript by Winter et al., entitled "The Ebola virus VP40 matrix undergoes endosomal disassembly essential for membrane fusion" describes the structural aspects of the events that precede Ebola virus (EBOV) membrane fusion in late endosome and virion uncoating in the cytosol. By combining state-of-the-art cryo-electron tomography (cryo-ET) with biophysical and computational techniques, they have elucidated the

pivotal role of the ebolaviral matrix virion protein 40 (VP40) in modulating the fusion process, in particular discovering that disassembly of the VP40 ordered lattice is low pH-driven, occurs despite the absence of a viral ion channel within the filovirus envelope and takes place through the weakening of VP40 interactions with lipids at the interface between the ebolaviral envelope and matrix.
Overall, the manuscript is well written and the research work is very well conceived, with solid orthogonal experimental approaches that mutually validate their respective results. It is opinion of this reviewer that the paper contributes to the elucidation of a key step in the EBOV infection cycle and that it will be of great interest for the readership of Review Commons and for the community of structural biologists.
Therefore, I recommend the publication of this paper, however after some minor revision to the text, the figures and the figure legends, which show inconsistencies in the terminology used, the acronyms and could be easily improved by some little graphical editing.

Comments:

- By starting their abstract and introduction sessions with the term "Ebola viruses" the authors are (on purpose?) preparing the reader to the implicit statement that their findings could be a paradigm model for the other members of the Ebolavirus genus. This is an exciting picture, especially in perspective of VP40-targeting drugs development. Therefore, although conclusions in this sense would probably require further studies, I encourage the authors to implement their figure 3 (or related supplementary figure) with a multiple-sequence alignment, and the relative text in the manuscript, by showing if and how much the basic patch at the C-terminus of VP40 is conserved within the Ebolavirus genus, especially the residues Lys224, Lys225, Lys274 and Lys275.

- It is a bit inconvenient for the reader to follow how a story unfolds while jumping back and forth between figures, and this is why I would recommend to move the period of the sentence at lines 88-91 to the session where figure 5 is discussed.

- Please, avoid the use of the slang "Ebola" without the apposition "virus", and make the text consistent throughout the manuscript by only using the acronym of each term after it was introduced for the first time.

Minor revisions:

Line 1: "matrix protein undergoes"

Line 19: "the matrix viral protein 40 (VP40)"

Line 18: considering that a virus "exists" in the form of a virion while temporarily located outside the cell, and as a "molecular entity" consisting of viral proteins and nucleic acids organised in macromolecular complexes during its life cycle inside the infected cell, this reviewer encourages the authors to rephrase as follows:
" Ebola viruses (EBOVs) virions are filamentous particles, ..."

Lines 35-36 and line 40: "that is determined by the matrix made up by the viral protein 40 (VP40), which drives ..." And then, directly use the acronym VP24 at line 40

Line 40: as VP24 and VP35 interact with NP but do not interact with the ssRNA genome, please rephrase as follows "the nucleoprotein (NP) which encapsidates the ssRNA genome, and the viral proteins VP24 and VP35 which, together with NP, form the nucleocapsid"

Lines 47-48: "...fusion glycoprotein (GP)...[...] the ebolaviral envelope"

Line 51: "...remarkably long virion of EBOVs undergoes..."

Line 63: "... in vitro, and in endo-lysosomal compartments in situ, by cryo-electron..."

Lines 70-71: " to shed light on EBOVs ... [...] with EBOV (Zaire ebolavirus species, Mayinga strain) in biosafety level 4 (BSL4) containment"

Line 72: chemically fixed by? (PFA and GA acronyms have been annotated in figure 1, but should be first mentioned in their explicit form in the text)

Line 73 (cryo-FIB)

Line 80: EBOV virions

Figure 1A and line 97: for consistency with the terminology used in the main text, should be perhaps in the second step preferred the term "vitrification" instead of cryofixation? Readers not familiar with the field could be confused by the use of the two synonyms

Lines 92-93: "...these data indicate [...] and suggest..."

Figure 1C and line 100: in the color legend EBOV is annotated as dark teal, however in the segmentation of the reconstructed tomogram there are three objects, one of which in dark teal is evidently a portion of EBOV virion inside the endosome, and other two are in different shades of green. What are those? Please, could author specify their identity in the figure legend with their corresponding color code? The same applies to supplementary figure S2 (see comment below).

Line 95: "...tomography of EBOV virions..."

Line 98: "...showing EBOV virions..." (This reviewer refers to the use of the term 'EBOVs' as for different species within the genus rather than for different EBOV particles within a dataset)

Line 105: "... a purified EBOV before..."

Line 110 and 113: "...EBOV matrix..." And "EBOV virus-like particles (VLP)"

Line 140, 141, 145 and 147: "EBOV VLPs" and "EBOV VLP"; idem at lines 188-189, 209 and anywhere else in the manuscript (including figure 4A)

Line 235: "influenza virus ion channel..."

Line 249: please, use directly the above-introduced acronym for the detergent

Figure 5F: in plot's X axis label: thermolysin (T)?

Line 342: "EBOV have remarkably long..."

Line 420 "...matrix-specific"

Line 464: "grids"

Line 465: "for cryo-FIB milling"

Line 611: "influenza virus M2 ..." (Please, from which influenza virus strain does the gene come from? Alternatively, which is the NCBI Protein and/or UniProt database code?)

Line 623: please, use the above-designated acronym for the detergent

Line 716: "...based on cryo-ET..."

Line 718: "influenza virus"

Line 734: "cryo-ET data"

Fig. S8: for consistency with the main text, "thermolysin"

Fig. S2, C and F: are these EBOV virions (as mentioned in the figure title) or EBOV VLPs (as the legends in the two panels of this figure seem to suggest)? Please, the authors should clarify

Line 1046: "malleable lipid envelope of the EBOV"; this adjective sounds confusing; the reviewer encourages the authors to rephrase for more clarity.

2. Significance:

Significance (Required)

see above.

3. How much time do you estimate the authors will need to complete the suggested revisions:

Estimated time to Complete Revisions (Required)

(Decision Recommendation)

Cannot tell / Not applicable

4. *Review Commons* values the work of reviewers and encourages them to get credit for their work. Select 'Yes' below to register your reviewing activity at Web of Science Reviewer Recognition Service (formerly Publons); note that the content of your review will not be visible on Web of Science.

Web of Science Reviewer Recognition

No

Review #3

1. Evidence, reproducibility and clarity:

Evidence, reproducibility and clarity (Required)

Winter and colleagues describe the molecular architecture of Ebola virus during entry into host cells. The main claims of the paper are that VP40 is disassembled prior to fusion. Disassembly is driven by the low pH environment in the endosomes. PH-induced uncoating works via "passive equilibration" because the Ebola virus envelope does not contain an ion channel. The authors conclude that structural remodeling of VP40 acts as a molecular switch coupling uncoating to fusion.

The main novel results of the manuscript are:

- In situ cryo-ET of endosomal compartments shows EBOV particles with intact condensed nucleocapsids and disordered protein densities that may relate to detached VP40.

- Five EBOV particles were imaged in the endosome and all had detached VP40 layers. Controls, budding virions and extracellular virions showed intact VP40 layers.

- Incubation of VP40-Gp VLPs with a pH 4.5 buffer leads to the disorder of the VP40 matrix in vitro, which is independent of Gp presence in the VLPs.

- MD simulation showed VP40 dimer binding to model membranes containing 30 % PS at pH7 and reduced binding at pH 4.5.

- Lipidomics revealed the lipid composition of VP40-Gp VLPs demonstrating 9% PS.

- VP40-PHluorin fusions were used to determine acidification of VLPs in vitro and to calculate a permeability coefficient of 1.2 Å sec-1, which is quite low compared to the permeability of the plasma membrane (345 Å sec-1).

- Next they modeled membrane fusion showing that fusion is more favorable after VP40 disassembly, especially favoring stalk formation.

- The authors propose further that fusion pore opening is more favorable in the presence of VP40.

- The authors claim that strong interactions of lipids with VP40 stabilizes the hemifusion intermediate.

- VP40 Gp VLPs can enter host cells independent of pH once Gp has been activated by thermolysin.

- Some of the results are over interpreted and require appropriate modifications.

Main points that need to be addressed:

- Imperfections of the membrane could be induced by proteins. Does acidification of the virion depend on GP and its transmembrane region? This can be tested with chimeric GP replacing its TM by unrelated trimeric TMs.

- Virus entry assays, line 292. The low pH is not only used for Gp cleavage, but induces the conformational changes leading to the post fusion conformation of Gp2. The authors need to check what happens to Gp once it is cleaved by thermolysin. Is this sufficient to induce the conformational changes in Gp? And if so how does entry of such VLPs work, because once the conformational change is triggered, GP2 will adopt the post fusion conformation which is inactive in fusion. This requires further clarification.

- In the fusion model, the authors claim that VP40 disassembly is more favorable for stalk formation, which is likely true. However, they also claim that strong VP40 interaction, which I would interpret as VP40 filaments interacting with the membrane, favor fusion pore opening. The tomograms and the in vitro experiments with VLPs indicate that the complete VP40 matrix is detached from the membrane under low pH conditions.
VLPs are purified. Can the authors exclude the possibility that the purification protocol does not damage the VLP membrane leading to in vitro acidification in a low pH environment?

- Can some of the assays be repeated with non-purified VLPs?

- Does acidification only work at pH 4.5?

Minor points

- Line 37: Ruigrok et al. 2000 J Mol Biol showed first that Ebola VP40 requires negatively charged lipids for interaction.

- Fig. 1f: Is VP40 detaching as a filament?

- References 8 and 28 are the same.

- Lipidomics: The authors find only 9% PS in the VLPs. How do these results compare to the composition of other enevloped viruses that have been reported to assemble on negatively charged lipids.

- EBO virus was suggested to assemble at lipid rafts. Is this reflected by the lipid composition?

2. Significance:

Significance (Required)

In summary, the manuscript is of high technical quality and the observation that VP40 detaches from the viral membrane prior to membrane fusion is novel and interesting to the field of virus fusion. How acidification occurs in the absence of an ion channel remains to be determined. The authors provide little insight how this might work.

The strong part of the manuscript is the EM part, which shows convincing detachement of the VP40 matrix. I cannot comment too much on the modelling part, which, however, sounds solid.

3. How much time do you estimate the authors will need to complete the suggested revisions:

Estimated time to Complete Revisions (Required)

(Decision Recommendation)

Less than 1 month

4. *Review Commons* values the work of reviewers and encourages them to get credit for their work. Select 'Yes' below to register your reviewing activity at [Web of Science Reviewer Recognition Service]() (formerly Publons); note that the content of your review will not be visible on Web of Science.

Web of Science Reviewer Recognition

Yes

Reviewer #1

Reviewer #1 (evidence, reproducibility and clarity (required)):

Winter et al. present a study of Ebola virus fusion in the acidic environment of the late endosome. Based on cryo-ET of Ebola virions undergoing entry into cells, they note that the VP40 matrix is disassembled and dissociated from the viral membrane in virions seen in the endosome. Subsequent in vitro and computational analyses suggest that protons diffuse across the viral membrane and neutralize anionic lipids on the inner leaflet. They argue that this loss of negative charge reduces the affinity of VP40 for the viral membrane. They further suggest that VP40 dissociation from the viral membrane precedes GP-mediated membrane fusion and contributes to reduction in the energy barrier for membrane stalk formation. Whereas most studies have focused on the importance of acidic pH in triggering GP conformational changes during fusion, the present work contributes new appreciation for VP40-membrane interactions.

We would like to thank the reviewer for all the insightful comments and appreciation of the novelty.

In the cryo-ET experiments aimed at visualizing Ebola entry, do the authors see evidence of viral membrane fusion? There is no mention of this in the text. Knowing that the virions that show disassembly of the VP40 matrix are in fact the virions that productively enter cells would support the conclusions of the study. As is stands, one is forced to wonder whether the virions that show VP40 disassembly prior to fusion ultimately fuse.

We first note that the EBOV virions shown in Figure 1 entering host cells were captured by cryo-ET at 48 hours post infection and resulted from 2-3 rounds of infection, thus the virions can productively enter the cells by micropinocytosis. Virions that are not able to undergo membrane fusion would be processed in the lysosomes and would not be detectable by cryo-ET at 48 hours post infection. In addition, the virions captured in late endosomes contain nucleocapsids, hence these virions are likely infectious. Together, this is good evidence that we really see events after successful membrane fusion.

We fully agree with the reviewer that capturing a fusion event would provide further proof that fusion depends on prior disassembly of the VP40 matrix layer. To address this, we acquired additional data on cells infected at different time-points post-infection (15 cells imaged); regrettably, we have not been successful in capturing a membrane fusion event, presumably due its fast kinetics. In this study we are technically limited with the amount of the virus we can use for infection in BSL4. The current dataset was generated at an MOI of 0.1 and this makes capturing entry events difficult as we would need an MOI of at least 100-1000 to increase the chances of capturing such a rare event.

Considering the technical difficulties to perform the experiment under BSL4 conditions, we have in addition performed a similar experiment using EBOV VLPs at high concentration (estimated MOI > 100) composed of VP40 and GP (Fig. S5). Despite the high VLP concentration, we could only find 2 tomograms out of 18 tomograms showing VLP entry events. These clearly show that the VP40 matrix is disassembled in VLPs residing in endosomes. The same lamellae displayed sites of viral fusion as evident from enlarged endosomal membrane surfaces studded with GPs facing endosomal lumina. Hence, this new data supports our results that VLPs that undergo VP40 disassembly are able to fuse. We have included the new supplementary figure S5 and added the following sentence to the main text:

Lines 96-102: "We were not able to capture virions residing in endosomes in the process of fusing with the endosomal membrane, presumably because virus membrane fusion is a rapid event. However, in a similar experiment using EBOV VLPs composed of VP40 and GP, we could confirm the absence of ordered VP40 matrix layers in VLPs inside endosomal compartments. Moreover, we were able to capture one fusion event and several intracellular membranes studded with luminal GPs, indicating that fusion had taken place (Fig. S5)."

In the cryo-ET experiments that evaluate VP40 disassembly in vitro, why do the authors leave out NP from their VLP preparations? There is some evidence in the literature (Li et al., JVI 2016) that NP is necessary to form

particles with native morphology. If the authors feel that NP is not necessary for their experiment, perhaps this could be noted.

Thank you very much for this important comment. Throughout this study, we mainly focused on the fate of the VP40 matrix during entry and thus reduced the complexity of the VLPs used to the minimum – VP40 and GP, so indeed NP was left out before. To address the role of the nucleocapsid in Ebola VLPs uncoating, we have now also included data on VLPs prepared by expression of nucleocapsid components (NP, VP24 and VP35) in addition to GP and VP40. Cryo-ET analysis of these VLPs showed that VLPs mainly contain loosely coiled nucleocapsid. This is consistent with a study by Bharat et al 2012, which shows that compared to virions, VLPs displayed heterogeneous nucleocapsid assembly states and reduced incorporation of nucleocapsids. It is important to note that VLPs containing nucleocapsid also displayed disassembled VP40 matrices at low pH (Fig. S7). Hence, nucleocapsid proteins do not influence the VP40 disassembly driven by low pH and GP-VP40 VLPs can be used as model to study VP40 uncoating.

We included a statement shown on lines 150-153: "We further repeated the experiment using VLPs composed of VP40, GP and the nucleocapsid proteins NP, VP24 and VP35, and observed the same low pH-phenotype described above. These results show that nucleocapsid proteins do not influence the VP40 disassembly driven by low pH."

The authors argue that acidic pH neutralizes the charge of PS phospholipids, thereby removing the electrostatic interactions of basic residues in VP40 and PS. They also note in the Methods section that 7 amino acids in VP40 are predicted by PROPKA to be protonated at pH 4.5. If the authors feel that protonation of these 7 amino acids is not involved in the loss of affinity for PS, this could be stated explicitly and justified. Could the protonation of these 7 amino acids contribute to disassembly of the VP40 lattice, rather than dissociation from the membrane?

Thank you for this interesting comment. We note that the amino acids predicted to be protonated (E76, E325, H61, H124, H210, H269, H315, ***see below****) are far away from the interaction interface with the membrane and also away from the intra-dimerization domain. Hence, they do not likely contribute to the loss of affinity for PS but may contribute to conformational changes that facilitate the disassembly of the VP40 matrix. For clarification, we have added the following statement to the methods section:*

Lines 541-544: "Importantly, these residues are located away from the interaction interface of VP40 with the membrane and their protonation accordingly does not influence membrane-binding. However, protonation of these residues may contribute to conformational changes that facilitate the VP40 matrix disassembly. "

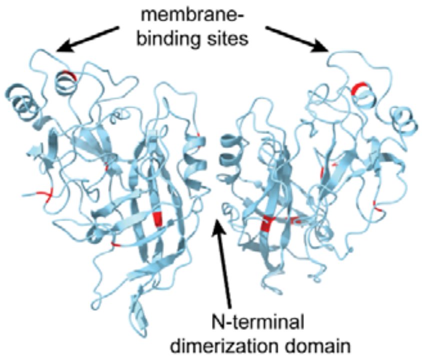

Minor: Figure S5C is difficult to interpret. The red frame on the bars that indicates data acquired at low pH is nearly invisible. Better might be to indicate explicitly (ie, with words) the pH at which data were acquired.

Thank you very much for this comment. We have changed the design of the graph accordingly. Please note that the figure numbering has changed and that Figure S5C is now Figure S6C.

Reviewer #1 (significance (required)):

The significance of the study stems from the idea that the VP40 lattice and its interaction with the viral membrane plays a direct role in facilitating viral fusion. To my knowledge, this has not been previously addressed. The significance would be considerably increased if the authors were able to demonstrate by cryo-ET that the virions with disassembled VP40 were in fact the virions that productively fused. Nonetheless, this work should be of broad interest to researchers studying viral fusion as it may represent a phenomenon relevant to numerous viruses that enter cells via the endocytic route.

Reviewer #2

Reviewer #2 (evidence, reproducibility and clarity (required)):

The manuscript by Winter et al., entitled "The Ebola virus VP40 matrix undergoes endosomal disassembly essential for membrane fusion" describes the structural aspects of the events that precede Ebola virus (EBOV) membrane fusion in late endosome and virion uncoating in the cytosol. By combining state-of-the-art cryo-electron tomography (cryo-ET) with biophysical and computational techniques, they have elucidated the pivotal role of the ebolaviral matrix virion protein 40 (VP40) in modulating the fusion process, in particular discovering that disassembly of the VP40 ordered lattice is low pH-driven, occurs despite the absence of a viral ion channel within the filovirus envelope and takes place through the weakening of VP40 interactions with lipids at the interface between the ebolaviral envelope and matrix.
Overall, the manuscript is well written and the research work is very well conceived, with solid orthogonal experimental approaches that mutually validate their respective results. It is opinion of this reviewer that the paper contributes to the elucidation of a key step in the EBOV infection cycle and that it will be of great interest for the readership of Review Commons and for the community of structural biologists.
Therefore, I recommend the publication of this paper, however after some minor revision to the text, the figures and the figure legends, which show inconsistencies in the terminology used, the acronyms and could be easily improved by some little graphical editing.

Thank you very much for your positive feedback and your comments.

Comments:

- By starting their abstract and introduction sessions with the term "Ebola viruses" the authors are (on purpose?) preparing the reader to the implicit statement that their findings could be a paradigm model for the other members of the Ebolavirus genus. This is an exciting picture, especially in perspective of VP40-targeting drugs development. Therefore, although conclusions in this sense would probably require further studies, I encourage the authors to implement their figure 3 (or related supplementary figure) with a multiple-sequence alignment, and the relative text in the manuscript, by showing if and how much the basic patch at the C-terminus of VP40 is conserved within the Ebolavirus genus, especially the residues Lys224, Lys225, Lys274 and Lys275.

Thank you very much for this comment. We have added a corresponding sequence alignment highlighting the high conservation of the basic patch of amino acids across all Ebola virus species (Suppl. Fig. S6). In the text, we refer to the sequence conservation as follows:

Lines 213-215: "These interactions are driven by basic patches of amino acids which are highly conserved across all EBOV species (Fig. S8 H), further emphasizing their importance in adaptable membrane binding."

- It is a bit inconvenient for the reader to follow how a story unfolds while jumping back and forth between figures, and this is why I would recommend to move the period of the sentence at lines 88-91 to the session where figure 5 is discussed.

We refer in fact to Figure 1 and fixed the reference accordingly (line 95).

- Please, avoid the use of the slang "Ebola" without the apposition "virus", and make the text consistent throughout the manuscript by only using the acronym of each term after it was introduced for the first time.

Thank you for this comment. We have thoroughly revised the use of technical terms.

Minor revisions:

Line 1: "matrix protein undergoes"

We refer here to the entire VP40 matrix layer composed of many VP40 proteins and not to single VP40 proteins (as the individual proteins do not disassemble, but their macromolecular assembly does). For clarification, we changed the title to "matrix layer undergoes".

Line 19: "the matrix viral protein 40 (VP40)"

We have corrected the statement.

Line 18: considering that a virus "exists" in the form of a virion while temporarily located outside the cell, and as a "molecular entity" consisting of viral proteins and nucleic acids organised in macromolecular complexes during its life cycle inside the infected cell, this reviewer encourages the authors to rephrase as follows: " Ebola viruses (EBOVs) virions are filamentous particles, ..."

Thank you for your suggestion. We have rephrased it to: „Ebola viruses (EBOVs) assemble into filamentous virions" (line 18).

Lines 35-36 and line 40: "that is determined by the matrix made up by the viral protein 40 (VP40), which drives ..." And then, directly use the acronym VP24 at line 40

We have corrected the statement.

Line 40: as VP24 and VP35 interact with NP but do not interact with the ssRNA genome, please rephrase as follows "the nucleoprotein (NP) which encapsidates the ssRNA genome, and the viral proteins VP24 and VP35 which, together with NP, form the nucleocapsid"

We have corrected the statement.

Lines 47-48: "...fusion glycoprotein (GP)...[...] the ebolaviral envelope"

We have corrected the statement.

Line 51: "...remarkably long virion of EBOVs undergoes..."

We have rephrased the statement: line 55: "…remarkably long EBOV virions undergo…"

Line 63: "... in vitro, and in endo-lysosomal compartments in situ, by cryo-electron..."

We have corrected the statement.

Lines 70-71: " to shed light on EBOVs ... [...] with EBOV (Zaire ebolavirus species, Mayinga strain) in biosafety level 4 (BSL4) containment"

We have corrected the statement.

Line 72: chemically fixed by? (PFA and GA acronyms have been annotated in figure 1, but should be first mentioned in their explicit form in the text)

We have now mentioned annotations for GA and PFA both in the main text and in the figure legend in their explicit forms.

Line 73 (cryo-FIB)

We have corrected the acronym.

Line 80: EBOV virions

We have corrected the statement.

Figure 1A and line 97: for consistency with the terminology used in the main text, should be perhaps in the second step preferred the term "vitrification" instead of cryofixation? Readers not familiar with the field could be confused by the use of the two synonyms

We have replaced the term as suggested.

Lines 92-93: "...these data indicate [...] and suggest..."
We have corrected the statement.

Figure 1C and line 100: in the color legend EBOV is annotated as dark teal, however in the segmentation of the reconstructed tomogram there are three objects, one of which in dark teal is evidently a portion of EBOV virion inside the endosome, and other two are in different shades of green. What are those? Please, could author specify their identity in the figure legend with their corresponding color code? The same applies to supplementary figure S2 (see comment below).

Thank you very much for this comment. All three green objects are EBOV virions. For clarification, we have added numbers 1-3 to the figure and legend and adjusted the text in the legend accordingly (lines 109-110).

Line 95: "...tomography of EBOV virions..."
We have corrected the statement.

Line 98: "...showing EBOV virions..." (This reviewer refers to the use of the term 'EBOVs' as for different species within the genus rather than for different EBOV particles within a dataset)
We have corrected the statement.

Line 105: "... a purified EBOV before..."
We realized a mistake in our phrasing: the virion shown in Fig. 1 H is not purified, but a virion found adjacent to the plasma membrane of an infected cell. We have changed the phrasing accordingly (lines 117-118).

Line 110 and 113: "...EBOV matrix..." And "EBOV virus-like particles (VLP)"
We have corrected the statement.

Line 140, 141, 145 and 147: "EBOV VLPs" and "EBOV VLP"; idem at lines 188-189, 209 and anywhere else in the manuscript (including figure 4A)
We have corrected the use of "EBOV VLP(s)" as suggested.

Line 235: "influenza virus ion channel..."
We have corrected the statement.

Line 249: please, use directly the above-introduced acronym for the detergent
We have revised the use of acronyms.

Figure 5F: in plot's X axis label: thermolysin (T)?
Yes, this is correct and stated in the figure legend.

Line 342: "EBOV have remarkably long..."
We have corrected the statement.

Line 420 "...matrix-specific"
We have corrected the spelling error.

Line 464: "grids"
We have corrected the spelling error.

Line 465: "for cryo-FIB milling"
We have corrected the statement.

Line 611: "influenza virus M2 ..." (Please, from which influenza virus strain does the gene come from? Alternatively, which is the NCBI Protein and/or UniProt database code?)

We have added the information to the Methods (line 648*): "….A/Udorn/307/1972 (subtype H3N2))…"*

Line 623: please, use the above-designated acronym for the detergent
We have used the acronym as suggested.

Line 716: "...based on cryo-ET..."
We have corrected the statement.

Line 718: "influenza virus"
We have corrected the term.

Line 734: "cryo-ET data"
We have corrected the term.

Fig. S8: for consistency with the main text, "thermolysin"
We have corrected the spelling of thermolysin throughout the manuscript.

Fig. S2, C and F: are these EBOV virions (as mentioned in the figure title) or EBOV VLPs (as the legends in the two panels of this figure seem to suggest)? Please, the authors should clarify
Thank you very much for spotting this mistake! These are indeed EBOV virions and we have changed the legends within the figure accordingly.

Line 1046: "malleable lipid envelope of the EBOV"; this adjective sounds confusing; the reviewer encourages the authors to rephrase for more clarity.
*We have removed the adjective „*malleable".

Reviewer #2 (significance (required)):
see above.

Reviewer #3 Reviewer #3 (evidence, reproducibility and clarity (required)):

Winter and colleagues describe the molecular architecture of Ebola virus during entry into host cells. The main claims of the paper are that VP40 is disassembled prior to fusion. Disassembly is driven by the low pH environment in the endosomes. PH-induced uncoating works via "passive equilibration" because the Ebola virus envelope does not contain an ion channel. The authors conclude that structural remodeling of VP40 acts as a molecular switch coupling uncoating to fusion.
The main novel results of the manuscript are:
In situ cryo-ET of endosomal compartments shows EBOV particles with intact condensed nucleocapsids and disordered protein densities that may relate to detached VP40.
Five EBOV particles were imaged in the endosome and all had detached VP40 layers. Controls, budding virions and extracellular virions showed intact VP40 layers.
Incubation of VP40-Gp VLPs with a pH 4.5 buffer leads to the disorder of the VP40 matrix in vitro, which is independent of Gp presence in the VLPs.
MD simulation showed VP40 dimer binding to model membranes containing 30 % PS at pH7 and reduced binding at pH 4.5.
Lipidomics revealed the lipid composition of VP40-Gp VLPs demonstrating 9% PS.

VP40-PHluorin fusions were used to determine acidification of VLPs in vitro and to calculate a permeability coefficient of 1.2 Å sec-1, which is quite low compared to the permeability of the plasma membrane (345 Å sec-1).
Next they modeled membrane fusion showing that fusion is more favorable after VP40 disassembly, especially favoring stalk formation.
The authors propose further that fusion pore opening is more favorable in the presence of VP40.
The authors claim that strong interactions of lipids with VP40 stabilizes the hemifusion intermediate.
VP40 Gp VLPs can enter host cells independent of pH once Gp has been activated by thermolysin.

We thank the reviewer for these interesting comments and valuable suggestions.

Some of the results are over interpreted and require appropriate modifications.

Main points that need to be addressed:
Imperfections of the membrane could be induced by proteins. Does acidification of the virion depend on GP and its transmembrane region? This can be tested with chimeric GP replacing its TM by unrelated trimeric TMs.

We agree that this is important to consider. We have addressed this question in Fig. 2 K using VLPs composed of VP40 alone. These VLPs lack GP and still display luminal acidification as evident from the disassembled VP40 matrix when incubated at low pH. Therefore, acidification does not depend on GP. For clarification, we have adjusted the following sentence in the discussion:

Lines 410-413: "Using VLPs of minimal protein composition (VP40 and GP, and VP40 alone), we show that VP40-disassembly, i.e. the detachment of the matrix from the viral envelope is triggered by low endosomal pH (Fig. 2). This indicates that VP40 disassembly does not depend on structural changes of other viral proteins, ***including GP****, and is driven solely by the acidic environment."*

Virus entry assays, line 292. The low pH is not only used for Gp cleavage, but induces the conformational changes leading to the post fusion conformation of Gp2. The authors need to check what happens to Gp once it is cleaved by thermolysin. Is this sufficient to induce the conformational changes in Gp? And if so how does entry of such VLPs work, because once the conformational change is triggered, GP2 will adopt the post fusion conformation which is inactive in fusion. This requires further clarification.

To our knowledge, there is only one study showing that EBOV GP2 changes conformation at low pH in the form of a re-arrangement of the fusion peptide from an extended loop to a kinked conformation (Gregory et al 2011). Importantly, low pH alone is not sufficient to trigger GP mediated membrane fusion and NPC1 is needed as a

trigger for membrane fusion process (Das et al, 2020). Hence proteolytically processed GP requires NPC1 binding to change its conformation to post-fusion state. We addressed this question by using pre-cleaved (= GP2) and low pH- treated VLPs in our entry assay (Fig. 5 F). Since low pH-treated VLPs enter host cells as efficiently as VLPs incubated at neutral pH, and low pH-treated and additionally pre-cleaved VLPs enter even more efficiently, it is highly unlikely that low pH triggers the post-fusion conformation as this should inhibit virus entry (as the reviewer pointed out). In conclusion, low pH does not induce the post-conformation in GP2 and we have included a respective sentence for clarification:

Lines 339-343: "Since thermolysin-treated EBOV VLPs efficiently enter untreated host cells at neutral and low pH, we further conclude that low pH alone does not induce the GP2 post-fusion conformation, which would inhibit virus entry. Together, this suggests a role of low endosomal pH beyond proteolytic processing of EBOV GP, likely for the disassembly of the VP40 matrix."

In the fusion model, the authors claim that VP40 disassembly is more favorable for stalk formation, which is likely true. However, they also claim that strong VP40 interaction, which I would interpret as VP40 filaments interacting with the membrane, favor fusion pore opening. The tomograms and the in vitro experiments with VLPs indicate that the complete VP40 matrix is detached from the membrane under low pH conditions.

We would like to stress that the modelling results for hemifusion formation and pore opening are independently calculated but have to be interpreted together because they occur sequentially. Hemifusion precedes formation of the pore and hence even though the model shows that the fusion pore opening is favored in the presence of VP40 interaction, membrane fusion cannot proceed to this stage because hemifusion is blocked until the VP40 matrix layer disassembles from the membrane. We apologize for lack of clarity, and we have added the sentences:

Lines 315-318: "However, it is important to note that hemifusion precedes pore formation in the membrane fusion pathway. Since the disassembly of the VP40 matrix is required for hemifusion and hence for the initiation of membrane fusion, it determines the outcome of the membrane fusion pathway."

VLPs are purified. Can the authors exclude the possibility that the purification protocol does not damage the VLP membrane leading to in vitro acidification in a low pH environment? Can some of the assays be repeated with non-purified VLPs?

Thank you very much for this important comment. To address this question, we had performed the cryo-ET experiments using purified and unpurified VLPs and found that they are virtually indistinguishable. Importantly, unpurified VLPs also undergo VP40 disassembly. We now show images from unpurified VLPs in a supplementary figure (Fig. S7). Thereby, the manuscript contains data of purified VLPs while we also provide proof that the purification protocol does not influence the disassembly of the VP40 matrix. We added the following explanatory sentence to the main text:

Lines 151-156: *"We further repeated the experiment using VLPs composed of VP40, GP and the nucleocapsid proteins NP, VP24 and VP35, and observed the same low pH-phenotype described above (Fig. S5 C). Performing the experiments on unpurified VLPs harvested from the supernatant of transfected cells confirmed that the purification protocol applied did not influence the disassembly of the VP40 matrix (Fig. S7). "*

Does acidification only work at pH 4.5?

We also attempted to verify the acidification of VLPs at higher pH (~5.5. and ~6.0) by cryo-ET, however, subtle structural differences were difficult to quantify. Considering the lower permeability of the VLP membrane compared to the plasma membrane, we think that acidification occurs indeed also at higher pH (as shown for cells), albeit at slower kinetics.

Minor points
Line 37: Ruigrok et al. 2000 J Mol Biol showed first that Ebola VP40 requires negatively charged lipids for interaction.

Thank you for pointing out this reference. We have included it in the text.

Fig. 1f: Is VP40 detaching as a filament?

We have not observed that VP40 detaches as a filament or a linear segment of multiple VP40 dimers. Since the VP40 dimer is inherently flexible (Fig. 3, Fig. S8) and can rotate along the N- and C-terminal intra- and inter-dimer interfaces, we believe disassembly occurs in a non-ordered fashion (not as filaments, see also Figure 2 G-K).

References 8 and 28 are the same.
We have corrected the reference duplication.

Lipidomics: The authors find only 9% PS in the VLPs. How do these results compare to the composition of other enevloped viruses that have been reported to assemble on negatively charged lipids.

We compared the lipid composition of the EBOV VLPs to the lipid composition of influenza viruses and HIV, which both bud from the plasma membrane and require negatively charged lipids. When grown in eggs, the envelope of influenza viruses contains 22-25 % PS (Ivanova et al 2015, Li et al 2011), and approximately 12% when produced from MDCK cells (Gerl et al 2012). The envelope of HIV virions produced from HeLa or MT4 cells contains 10-15% PS. These numbers suggest that the producing cell line strongly influences the lipid composition of the virus particles. Besides differences in the producing cell line, the lower amount of PS found in EBOV VLPs could have multiple implications: first, apart from PS, PIP2 has also been shown to interact specifically with VP40 at budding sites in the plasma membrane (Jeevan et al 2017, Johnson et al 2018) and thus also contributes to virion assembly (potentially allowing for a lower PS concentration); second, as recently shown for paramyxoviruses (Norris et al 2022), binding of PS to viral proteins is not based on charge alone but may include specific binding – in which case a high affinity of viral proteins to PS may allow for a lower PS concentration in the target membrane. Overall, the rather low PS content in Ebola VLPs might be important for VP40 interaction and low pH-driven disassembly.

EBO virus was suggested to assemble at lipid rafts. Is this reflected by the lipid composition?

Yes, that is correct. A hallmark of lipid rafts is the enrichment of cholesterol and sphingomyelin (~32 mol% cholesterol, ~ 14 mol% sphingomyelin) in the microdomains (Pike et al 2002). The lipid composition of the EBOV VLPs determined in our study (~ 39% cholesterol and ~10 mol% sphingomyelin) is consistent with the assembly at lipid rafts. Minor differences stem from the different cell lines and lipidomic approaches used to determine the lipid species.

Reviewer #3 (significance (required)):
In summary, the manuscript is of high technical quality and the observation that VP40 detaches from the viral membrane prior to membrane fusion is novel and interesting to the field of virus fusion. How acidification occurs in the absence of an ion channel remains to be determined. The authors provide little insight how this might work.
The strong part of the manuscript is the EM part, which shows convincing detachement of the VP40 matrix. I cannot comment too much on the modelling part, which, however, sounds solid.

Dear Dr Chlanda,

Please note that we have received the checks from our publisher, so please include the following requirements in your revised manuscript:

- Our publisher has done their pre-publication check on your manuscript. When you log into the manuscript submission system you will see the file "Data Edited Manuscript file". Please take a look at the word file (attached for your convenience) and the comments regarding the figure legends and respond to the issues.

- Please correct: Very little image change in figure 4C - but they are there. PH 7.4 0-10 mins.

- Table 1 and 2 should be renamed to Dataset EV1-EV2 with the appropriate callouts, and legends uploaded as separate tabs in each Excel file

We look forward to receiving your updated manuscript as soon as possible.

Kind regards,
Lela

Lela Djordjevic-Ristanovic
Editorial Assistant
EMBO Press

~~~~~~~~~~~~~~~~~~~~~~~~~

Dear Dr Chlanda,

We have now received re-review reports of your manuscript EMBOJ-2023-113578 from two of the three original Review Commons referees. As you will see, you have addressed all of their concerns satisfactorily.

However, before I can formally accept your work for publication, there are some remaining editorial points which need to be addressed. In this regard would you please:

- remove all figures from the "Data edited ms file",

- rename your "Data and materials availability section", the "Data Availability Section",

- include up to five key words,

- acknowledge grant number TRR 83 in our online submission portal,

- organize the reference section in alphabetical order, using up to ten author names, and + et al. whenever this is exceeded,

- rename the Conflict of Interest Section the "DISCLOSURE AND COMPETING INTERESTS STATEMENT",

- remove the author credit section from the manuscript,

- change the order of the figure callouts so Figure 2E comes after 2D; there is a missing callout for Figure 5E,

- upload a completed author checklist,

- upload all figures in separate files; main figures should be uploaded as individual files at high-resolution, their legends should be listed after the References in the main manuscript file,

- include a table of contents page numbers in appendix 1 using the nomenclature Appendix Figure S1-S10 with the corresponding callouts in the text, and

- rename the movie "Movie EV1", including the corresponding callout, in the text and zipping the legend with the movie file.

We require the publication of source data. Please contact Hannah Sonntag (in cc) to discuss which Source data should be provided in your case.
~~~~~~~~~~~~~~~~~~~~~~~~~

We include a synopsis of the paper (see http://emboj.embopress.org/). Please provide me with a general summary statement and 3-5 bullet points that capture the key findings of the paper.

We also need a summary figure for the synopsis. The size should be 550 wide by [200-400] high (pixels). You can also use something from the figures if that is easier.

We encourage the publication of source data, particularly for electrophoretic gels and blots, with the aim of making primary data more accessible and transparent to the reader. It would be great if you could provide me with a PDF file per figure that contains the original, uncropped and unprocessed scans of all or key gels used in the figures. The PDF files should be labeled with the appropriate figure/panel number, and should have molecular weight markers; further annotation could be useful but is not essential. The PDF files will be published online with the article as supplementary "Source Data" files. Source Data can also include Excel tables to accompany your graphs. We anticipate that their inclusion will make your work more discoverable and useable to scientists in the future.

EMBO Press is an editorially independent publishing platform for the development of EMBO scientific publications.

Best wishes,

William Teale

William Teale, PhD
Editor
The EMBO Journal
w.teale@embojournal.org

Instructions for preparing your revised manuscript:

Please check that the title and abstract of the manuscript are brief, yet explicit, even to non-specialists.

When assembling figures, please refer to our figure preparation guideline in order to ensure proper formatting and readability in print as well as on screen:
https://bit.ly/EMBOPressFigurePreparationGuideline
See also figure legend guidelines: https://www.embopress.org/page/journal/14602075/authorguide#figureformat

IMPORTANT: When you send the revision we will require
- a point-by-point response to the referees' comments, with a detailed description of the changes made (as a word file).
- a word file of the manuscript text.
- individual production quality figure files (one file per figure)
- a complete author checklist, which you can download from our author guidelines (https://www.embopress.org/page/journal/14602075/authorguide).
- Expanded View files (replacing Supplementary Information)
Please see out instructions to authors
https://www.embopress.org/page/journal/14602075/authorguide#expandedview

Please remember: Digital image enhancement is acceptable practice, as long as it accurately represents the original data and conforms to community standards. If a figure has been subjected to significant electronic manipulation, this must be noted in the figure legend or in the 'Materials and Methods' section. The editors reserve the right to request original versions of figures and the original images that were used to assemble the figure.

Further information is available in our Guide For Authors: https://www.embopress.org/page/journal/14602075/authorguide

We realize that it is difficult to revise to a specific deadline. In the interest of protecting the conceptual advance provided by the work, we recommend a revision within 3 months (15th May 2023). Please discuss the revision progress ahead of this time with the editor if you require more time to complete the revisions. Use the link below to submit your revision:

https://emboj.msubmit.net/cgi-bin/main.plex

Referee #1:

The authors have addressed all questions and I support publication.

Referee #2:

The authors have addressed all of my comments. I can appreciate that capturing fusion events of Ebola virions using cryoET is challenging, and their efforts in this regard are notable, even if not entirely successful. I support publication of the work.

Response to reviewers

Reviewers comments:

Referee #1:

The authors have addressed all questions and I support publication.

Referee #2:

The authors have addressed all of my comments. I can appreciate that capturing fusion events of Ebola virions using cryoET is challenging, and their efforts in this regard are notable, even if not entirely successful. I support publication of the work.

Response to reviewers:

We would like thank again to the reviewers for their constructive comments and suggestions that help us to improve the manuscript.

Dear Petr,

I am pleased to inform you that your manuscript has been accepted for publication in the EMBO Journal.

Congratulations on a really elegant study!

Please note that it is EMBO Journal policy for the transcript of the editorial process (containing referee reports and your response letter) to be published as an online supplement to each paper. If you do NOT want this, you will need to inform the Editorial Office via email immediately. More information is available here:
https://www.embopress.org/page/journal/14602075/authorguide#transparentprocess

Your manuscript will be processed for publication in the journal by EMBO Press. Manuscripts in the PDF and electronic editions of The EMBO Journal will be copy edited, and you will be provided with page proofs prior to publication. Please note that supplementary information is not included in the proofs.

You will be contacted by Wiley Author Services to complete licensing and payment information. The required 'Page Charges Authorization Form' is available here: https://www.embopress.org/pb-assets/embo-site/tej_apc.pdf - please download and complete the form and return to embopressproduction@wiley.com

EMBO Press participates in many Publish and Read agreements that allow authors to publish Open Access with reduced/no publication charges. Check your eligibility: https://authorservices.wiley.com/author-resources/Journal-Authors/open-access/affiliation-policies-payments/index.html

Should you be planning a Press Release on your article, please get in contact with embojournal@wiley.com as early as possible, in order to coordinate publication and release dates.

If you have any questions, please do not hesitate to call or email the Editorial Office. Thank you for your contribution to The EMBO Journal.

Best wishes,

William

William Teale, PhD
Editor
The EMBO Journal
w.teale@embojournal.org

** Click here to be directed to your login page: https://emboj.msubmit.net

EMBO Press Author Checklist

Corresponding Author Name: Petr Chlanda
Journal Submitted to: EMBO J
Manuscript Number: EMBOJ-2023-113578

USEFUL LINKS FOR COMPLETING THIS FORM
The EMBO Journal - Author Guidelines
EMBO Reports - Author Guidelines
Molecular Systems Biology - Author Guidelines
EMBO Molecular Medicine - Author Guidelines

Reporting Checklist for Life Science Articles (updated January 2022)
This checklist is adapted from Materials Design Analysis Reporting (MDAR) Checklist for Authors. MDAR establishes a minimum set of requirements in transparent reporting in the life sciences (see Statement of Task: 10.31222/osf.io/9sm4x). Please follow the journal's guidelines in preparing your manuscript.
Please note that a copy of this checklist will be published alongside your article.

Abridged guidelines for figures
1. Data
The data shown in figures should satisfy the following conditions:
- the data were obtained and processed according to the field's best practice and are presented to reflect the results of the experiments in an accurate and unbiased manner.
- ideally, figure panels should include only measurements that are directly comparable to each other and obtained with the same assay.
- plots include clearly labeled error bars for independent experiments and sample sizes. Unless justified, error bars should not be shown for technical replicates.
- if n<5, the individual data points from each experiment should be plotted. Any statistical test employed should be justified.
- Source Data should be included to report the data underlying figures according to the guidelines set out in the authorship guidelines on Data Presentation.

2. Captions
Each figure caption should contain the following information, for each panel where they are relevant:
- a specification of the experimental system investigated (eg cell line, species name).
- the assay(s) and method(s) used to carry out the reported observations and measurements.
- an explicit mention of the biological and chemical entity(ies) that are being measured.
- an explicit mention of the biological and chemical entity(ies) that are altered/varied/perturbed in a controlled manner.
- the exact sample size (n) for each experimental group/condition, given as a number, not a range;
- a description of the sample collection allowing the reader to understand whether the samples represent technical or biological replicates (including how many animals, litters, cultures, etc.).
- a statement of how many times the experiment shown was independently replicated in the laboratory.
- definitions of statistical methods and measures:
  - common tests, such as t-test (please specify whether paired vs. unpaired), simple χ2 tests, Wilcoxon and Mann-Whitney tests, can be unambiguously identified by name only, but more complex techniques should be described in the methods section;
  - are tests one-sided or two-sided?
  - are there adjustments for multiple comparisons?
  - exact statistical test results, e.g., P values = x but not P values < x;
  - definition of 'center values' as median or average;
  - definition of error bars as s.d. or s.e.m.

Please complete ALL of the questions below.
Select "Not Applicable" only when the requested information is not relevant for your study.

Materials

Newly Created Materials	Information included in the manuscript?	In which section is the information available? (Reagents and Tools Table, Materials and Methods, Figures, Data Availability Section)
New materials and reagents need to be available; do any restrictions apply?	Not Applicable	

Antibodies	Information included in the manuscript?	In which section is the information available? (Reagents and Tools Table, Materials and Methods, Figures, Data Availability Section)
For **antibodies** provide the following information:<br>- Commercial antibodies: RRID (if possible) or supplier name, catalogue number and or/clone number<br>- Non-commercial: RRID or citation	Yes	Material and Methods

DNA and RNA sequences	Information included in the manuscript?	In which section is the information available? (Reagents and Tools Table, Materials and Methods, Figures, Data Availability Section)
**Short novel DNA or RNA including primers, probes**: provide the sequences.	Not Applicable	

Cell materials	Information included in the manuscript?	In which section is the information available? (Reagents and Tools Table, Materials and Methods, Figures, Data Availability Section)
**Cell lines:** Provide species information, strain. Provide accession number in repository **OR** supplier name, catalog number, clone number, and/**OR** RRID.	Yes	Materials and Methods
**Primary cultures:** Provide species, strain, sex of origin, genetic modification status.	Not Applicable	
Report if the cell lines were recently **authenticated** (e.g., by STR profiling) and tested for mycoplasma contamination.	Yes	Materials and Methods

Experimental animals	Information included in the manuscript?	In which section is the information available? (Reagents and Tools Table, Materials and Methods, Figures, Data Availability Section)
**Laboratory animals or Model organisms:** Provide species, strain, sex, age, genetic modification status. Provide accession number in repository **OR** supplier name, catalog number, clone number, **OR** RRID.	Not Applicable	
**Animal observed in or captured from the field:** Provide species, sex, and age where possible.	Not Applicable	
Please detail **housing and husbandry conditions**.	Not Applicable	

Plants and microbes	Information included in the manuscript?	In which section is the information available? (Reagents and Tools Table, Materials and Methods, Figures, Data Availability Section)
**Plants:** provide species and strain, ecotype and cultivar where relevant, unique accession number if available, and source (including location for collected wild specimens).	Not Applicable	
**Microbes:** provide species and strain, unique accession number if available, and source.	Yes	Materials and Methods

Human research participants	Information included in the manuscript?	In which section is the information available? (Reagents and Tools Table, Materials and Methods, Figures, Data Availability Section)
If collected and within the bounds of privacy constraints report on age, sex and gender or ethnicity for all study participants.	Not Applicable	

Core facilities	Information included in the manuscript?	In which section is the information available? (Reagents and Tools Table, Materials and Methods, Figures, Data Availability Section)
If your work benefited from core facilities, was their service mentioned in the acknowledgments section?	Yes	Acknowledgements

Design

Study protocol	Information included in the manuscript?	In which section is the information available? (Reagents and Tools Table, Materials and Methods, Figures, Data Availability Section)
If study protocol has been **pre-registered, provide DOI in the manuscript**. For clinical trials, provide the trial registration number **OR** cite DOI.	Not Applicable	
Report the **clinical trial registration number** (at ClinicalTrials.gov or equivalent), where applicable.	Not Applicable	

Laboratory protocol	Information included in the manuscript?	In which section is the information available? (Reagents and Tools Table, Materials and Methods, Figures, Data Availability Section)
Provide DOI OR other citation details if **external detailed step-by-step protocols** are available.	Not Applicable	

Experimental study design and statistics	Information included in the manuscript?	In which section is the information available? (Reagents and Tools Table, Materials and Methods, Figures, Data Availability Section)
Include a statement about **sample size** estimate even if no statistical methods were used.	Not Applicable	
Were any steps taken to minimize the effects of subjective bias when allocating animals/samples to treatment (e.g. **randomization procedure**)? If yes, have they been described?	Not Applicable	
Include a statement about **blinding** even if no blinding was done.	Not Applicable	
Describe **inclusion/exclusion criteria** if samples or animals were excluded from the analysis. Were the criteria pre-established?<br><br>If sample or data points were omitted from analysis, report if this was due to attrition or intentional exclusion and provide justification.	Not Applicable	
For every figure, are **statistical tests** justified as appropriate? Do the data meet the assumptions of the tests (e.g., normal distribution)? Describe any methods used to assess it. Is there an estimate of variation within each group of data? Is the variance similar between the groups that are being statistically compared?	Not Applicable	

Sample definition and in-laboratory replication	Information included in the manuscript?	In which section is the information available? (Reagents and Tools Table, Materials and Methods, Figures, Data Availability Section)
In the figure legends: state number of times the experiment was **replicated** in laboratory.	Yes	
In the figure legends: define whether data describe **technical or biological replicates**.	Yes	

Ethics

Ethics	Information included in the manuscript?	In which section is the information available? (Reagents and Tools Table, Materials and Methods, Figures, Data Availability Section)
Studies involving **human participants**: State details of **authority granting ethics approval** (IRB or equivalent committee(s), provide reference number for approval.	Not Applicable	
Studies involving **human participants**: Include a statement confirming that **informed consent** was obtained from all subjects and that the experiments conformed to the principles set out in the WMA Declaration of Helsinki and the Department of Health and Human Services Belmont Report.	Not Applicable	
Studies involving **human participants:** For publication of **patient photos**, include a statement confirming that consent to publish was obtained.	Not Applicable	
Studies involving experimental **animals**: State details of **authority granting ethics approva**l (IRB or equivalent committee(s), provide reference number for approval. Include a statement of compliance with ethical regulations.	Not Applicable	
Studies involving **specimen and field samples:** State if relevant **permits** obtained, provide details of authority approving study; if none were required, explain why.	Not Applicable	

Dual Use Research of Concern (DURC)	Information included in the manuscript?	In which section is the information available? (Reagents and Tools Table, Materials and Methods, Figures, Data Availability Section)
Could your study fall under dual use research restrictions? Please check biosecurity documents and list of **select agents and toxins** (CDC): https://www.selectagents.gov/sat/list.htm	Not Applicable	
If you used a select agent, is the security level of the lab appropriate and reported in the manuscript?	Yes	Materials and Methods
If a study is subject to dual use research of concern regulations, is the name of the **authority granting approval and reference number** for the regulatory approval provided in the manuscript?	Not Applicable	

Reporting

The MDAR framework recommends adoption of discipline-specific guidelines, established and endorsed through community initiatives. Journals have their own policy about requiring specific guidelines and recommendations to complement MDAR.

Adherence to community standards	Information included in the manuscript?	In which section is the information available? (Reagents and Tools Table, Materials and Methods, Figures, Data Availability Section)
State if relevant guidelines or checklists (e.g., **ICMJE, MIBBI, ARRIVE, PRISMA**) have been followed or provided.	Not Applicable	
For **tumor marker prognostic studies,** we recommend that you follow the **REMARK** reporting guidelines (see link list at top right). See author guidelines, under 'Reporting Guidelines'. Please confirm you have followed these guidelines.	Not Applicable	
For **phase II and III randomized controlled trials**, please refer to the **CONSORT** flow diagram (see link list at top right) and submit the CONSORT checklist (see link list at top right) with your submission. See author guidelines, under 'Reporting Guidelines'. Please confirm you have submitted this list.	Not Applicable	

Data Availability

Data availability	Information included in the manuscript?	In which section is the information available? (Reagents and Tools Table, Materials and Methods, Figures, Data Availability Section)
Have **primary datasets** been deposited according to the journal's guidelines (see 'Data Deposition' section) and the respective accession numbers provided in the Data Availability Section?	Yes	Data Availability Section
Were **human clinical and genomic datasets** deposited in a public access-controlled repository in accordance to ethical obligations to the patients and to the applicable consent agreement?	Not Applicable	
Are **computational models** that are central and integral to a study available without restrictions in a machine-readable form? Were the relevant accession numbers or links provided?	Yes	Data Availability Section
If publicly available data were reused, provide the respective **data citations in the reference list**.	Yes	Reference list
